# Cavities and Atomic Packing in Protein Structures and Interfaces

**DOI:** 10.1371/journal.pcbi.1000188

**Published:** 2008-09-26

**Authors:** Shrihari Sonavane, Pinak Chakrabarti

**Affiliations:** Department of Biochemistry, Bose Institute, Calcutta, India; MRC Laboratory of Molecular Biology, United Kingdom

## Abstract

A comparative analysis of cavities enclosed in a tertiary structure of proteins and interfaces formed by the interaction of two protein subunits in obligate and non-obligate categories (represented by homodimeric molecules and heterocomplexes, respectively) is presented. The total volume of cavities increases with the size of the protein (or the interface), though the exact relationship may vary in different cases. Likewise, for individual cavities also there is quantitative dependence of the volume on the number of atoms (or residues) lining the cavity. The larger cavities tend to be less spherical, solvated, and the interfaces are enriched in these. On average 15 Å^3^ of cavity volume is found to accommodate single water, with another 40–45 Å^3^ needed for each additional solvent molecule. Polar atoms/residues have a higher propensity to line solvated cavities. Relative to the frequency of occurrence in the whole structure (or interface), residues in β-strands are found more often lining the cavities, and those in turn and loop the least. Any depression in one chain not complemented by a protrusion in the other results in a cavity in the protein–protein interface. Through the use of the Voronoi volume, the packing of residues involved in protein–protein interaction has been compared to that in the protein interior. For a comparable number of atoms the interface has about twice the number of cavities relative to the tertiary structure.

## Author Summary

During protein folding a polypeptide chain takes up a three-dimensional structure that is characterized by close packing of atoms. For cellular processes proteins need to interact, and the binding is also characterized by packing of complementary surfaces. Two types of binding can be envisaged—obligate and non-obligate—the former is exhibited by homodimeric molecules (in which two polypeptide chains are held together in permanent association) and the latter by protein–protein complexes (such as antigen–antibody, enzyme–inhibitor, etc.), which are more transient in nature. Cavities are observed as defects in atomic packing. We present an analysis of cavities within the structure of a protein chain, as well as interfaces formed by the association of two protein chains. For a comparable number of atoms the interface has about twice the number of cavities relative to the tertiary structure. The interfaces contain a higher percentage of larger cavities, which tend to be solvated. We have determined the relationships between the protein volume and the total volume of all the cavities in the structure, the volume of the cavity and the number of atoms (residues) lining it, and the size of the cavity and the number of waters in it.

## Introduction

Close atomic packing is an important metric for characterizing protein structures—the average packing density for the interior of proteins is similar to that for crystals of small organic molecules [1]. While the average value of packing density in the protein interior is close to 0.75, the efficiency may not be uniform over the whole structure, the density varying in the range 0.66 to 0.84 [2]–[4]. The localized defects in packing show up as cavities [5], and when present they can reduce the stability of the structure [6]. Protein binding has many similar features common to folding, such as the presence of a core in the interface region [7],[8] and complementarities of chemical characteristics of residues in contact across the interface and the nature of the specific interactions linking them [9]–[11]. Although the surfaces that form the interface in protein-protein interaction have complementary shape [12],[13], an issue that has not been addressed is whether the interface can harbor cavities, and their features relative to those present in the protein interior.

Voronoi [14] procedure has been used to assign a unique volume to individual atoms in a collection of atoms, such as in proteins. This has been used to calculate the volumes occupied by amino acid residues and their variation at individual sites [3], [15]–[17]. As to associate a volume of space to an atom the procedure relies on the location of all its neighbors, it works well when applied to atoms in the protein interior. Distinct from the surface atoms those in the interface have surrounding atoms from the interacting protein chains, and as such one can calculate the Voronoi volumes associated to interface atoms [18], and compare these to those in the protein core.

Cavities in structures have also been looked into from the perspective of protein hydration [19]–[21]. Water molecules are also located in the interfaces [22]. Buried water molecules are often conserved among members in a homologous family and are integral structural component of these proteins [23]. When located in cavities they can compensate for the destabilization of reduced hydrophobic and van der Waals interactions [24]. It is of importance to know the average volume occupied by water molecules in protein interior and interfaces and the nature of their interactions with the surrounding protein atoms.

Clefts or pockets on the surface are important for molecular recognition and protein function [25]. Distinct from them are the cavities, defined as enclosed space in the interior of the protein. However, they are sometimes considered together, for example, for defining interior and surface packing densities [26]. Internal cavities have been analyzed separately for monomeric proteins [19],[21], as well as protein interfaces [27], but no attempt has been made to generalize their features from a common perspective. In this work we use a vastly enlarged repertoire of structures to quantify the geometrical characteristics of the cavities found in protein tertiary structures and interfaces—the latter being of two types—those involving obligate homodimeric assemblies and the non-obligate protein-protein heterocomplexes. Other features studied are the occurrence of solvent molecules in the cavities and their hydrogen bonding, the participation of different secondary structural elements in the cavities, the environment of cavity water in the interface, etc. Rather than being mere “packing defects” cavities are also known to play a role in assisting conformational changes between domains or subunit interfaces and in controlling binding and catalysis [28]. Thus a comprehensive analysis of cavities would provide insight into our understanding of protein structure and function.

## Results

**Figure 1:**
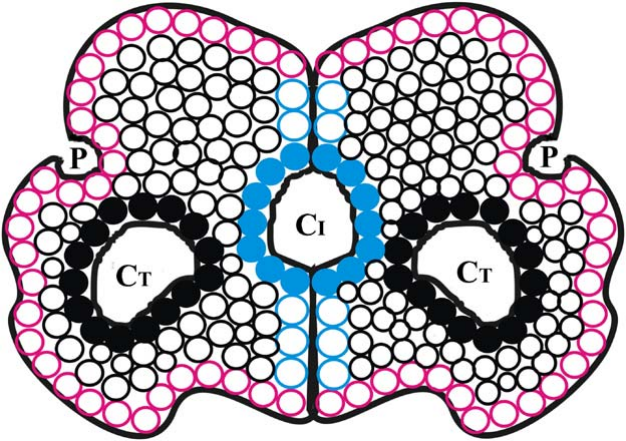
Schematic representation of cavities in a homodimeric molecule. Cavity-lining (CL) atoms are in filled circles, black for those in tertiary structures and blue in interfaces. Non-cavity-non-surface (NCNS) atoms in the tertiary structure are in open, black circles and those in the interface are in open, blue circles. Surface atoms are in red. Cavities within the tertiary structure (C_T_) and interface (C_I_) are distinct from the surface pockets (P).

Cavities and atoms considered in the analysis are defined in Figure 1. For an interface cavity at least 20% of the CL (cavity-lining) atoms ought to belong to a different subunit. If the contribution is less, the cavity is assumed to be part of the tertiary structure of a subunit. The cavities of a single subunit of homodimers and those of monomers were pulled together to constitute the Ter_str (tertiary structure) cavities. The reasons for leaving out individual subunits of protein-protein heterocomplexes are: in some complexes one of the protein components may itself be multimeric; in a few, one component may be rather small to be considered a ‘typical’ protein.

A total of 3384 Ter_str cavities were detected within 219 individual subunits (5 had no cavity) (Table S1). The homodimer dataset contained 4438 cavities, out of which 615 (14%) are Inter_H (occurring between the homodimer subunits) and the remaining 3823 (86%) occur within (contributing to Ter_str). About 8% interfaces and 2% subunits in homodimers are without any detectable cavity. Protein-Protein complexes had 3944 cavities in total, of which 431 (11%) are Inter_C (occurring in heterocomplex interface). Consideration of volume indicates that 24% and 14% of the total cavity volume in the homodimer and complex datasets, respectively, are contributed by interface cavities. ∼20% interfaces in protein-protein complexes are devoid of detectable cavities. The detailed information on cavities in individual PDB entries is provided in Table S2.

### Number of Cavities and Their Total Volume in Tertiary Structure and Interfaces

On average ∼15 cavities occur in the individual subunits in the monomer and the homodimer datasets and consequently these two categories were considered together to represent Ter_str (Table 1). Compared to the tertiary structure the interfaces have about a third and a sixth number of cavities in homodimers and complexes, respectively. However, the total cavity volume is reduced to two-third and one-fifth in the two types of interfaces, indicating that the cavities in interfaces are larger in size than those in the tertiary structure.

**Table 1:** Average values of the total number of cavities and the total cavity volume in protein tertiary structures and interfaces.

	Ter_str^b^			Interface	
	Monomer	Homodimer	Overall	Inter_H	Inter_C
No. of cavities	15 (12.6)	15.8 (14.9)	15.5 (13.9)	5.0 (4.5)	2.4 (2.1)
Normalized no.^a^	15 (6)	15 (6)	15 (6)	25 (14)	24 (17)
Total Cavity volume (Å^3^)	455 (455)	512 (561)	486 (517)	324 (498)	97 (127)
Normalized volume^a^	457 (247)	485 (244)	473 (245)	1585 (1439)	970 (1034)

The standard deviations are in parentheses.

a Number or volume (given in the previous row) per 2000 atoms. The volume of a 2,000 atom protein is about 49,000 Å^3^.

b As the values obtained from the individual subunits of the monomer and homodimer datasets gave similar values, these were merged to get the ‘Overall’ value.

Proteins are of different sizes; besides there is a lot of variation in the numbers of atoms constituting the tertiary structure and the interface. Consequently, we have also expressed the number of cavities and their total volume relative to an average-sized protein of 2000 atoms. Compared to Ter_str the interfaces have about 1.6 times the number of cavities, but the increase in total volume is 3.4 and 2.1 times in homodimers and heteocomplexes, respectively, again indicating the larger size of cavities in the interface, especially for homodimers. In general, the packing of residues in the interface leaves more cavities compared to that in the tertiary structure.

**Figure 2:**
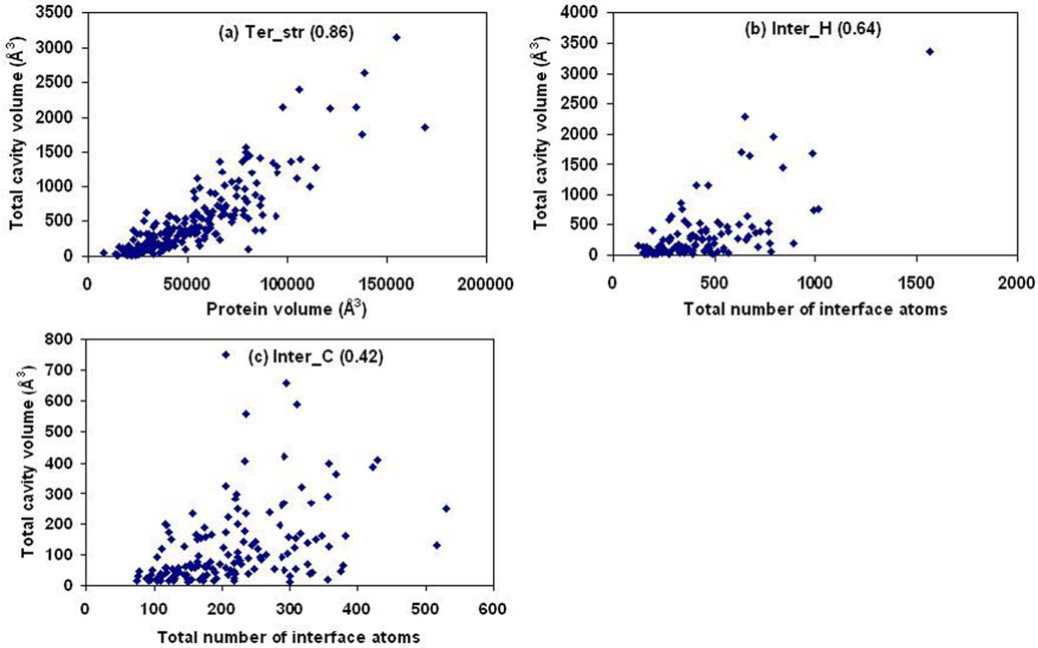
Dependence of the total volume of cavities on the volume (or the total number of atoms) of the protein (or the interface). Plot of total volume of cavities against (A) the volume of the protein tertiary structure, and (B,C) the total number of atoms in interfaces. The correlation coefficient, *r*, is given in parentheses. In (A) the equation for the least-squares line is *y* = 0.016*x*–311 (*R* ^2^ = 0.74); if approximated by a power law the corresponding equation is (A) *y* = 0.000002*x* ^1.779^ (*R* ^2^ = 0.70). If one uses the total number of atoms in the tertiary structure (in place of the volume) the distribution looks very similar to (A) and the two equations are *y* = 0.371*x*–279 (*R* ^2^ = 0.75) and *y* = 0.0009*x* ^1.687^ (*R* ^2^ = 0.71).

The total volume of Ter_str cavities in a subunit is well correlated to the size, as given by the protein volume (or the number of atoms) (Figure 2A). If the size of an interface is defined by the number of atoms belonging to it, the correlation with the total cavity volume is poor for interface cavities (Figure 2B and 2C). However, when individual cavities are considered the correlation of volume is very strong with both the numbers of cavity lining atoms and residues in all the three categories of cavities (Figure S1). Both linear relationships and equations using power law can fit the data equally well (Table 2), but the former would suggest a negative cavity volume when the number of CL atoms/residues is <4. Using the latter set of equations about 5 atoms or 4 residues are needed to enclose a volume (∼11.5 Å^3^) large enough to accommodate one water molecule.

**Table 2:** Equations describing the dependence of the volume on the number of atoms/residues lining individual cavities.

	Equation^a^ (and R^2^) Involving	
Cavity Class	Atoms	Residues
Ter_str	*y* = 1.9284*x* ^1.2836^ (0.90)	*y* = 1.402*x* ^1.7253^ (0.78)
Inter_H	*y* = 1.7544*x* ^1.3418^ (0.95)	*y* = 1.1917*x* ^1.8337^ (0.89)
Inter_C	*y* = 1.8507*x* ^1.3225^ (0.93)	*y* = 1.1907*x* ^1.8456^ (0.86)

a In the equation y corresponds to the volume (Å^3^) and *x* to the number of atoms (or residues); the *R* ^2^ value provides the measure of fit of the power law to the data points. Fitting linear equations provides, for atoms: *y* = 6.59*x*–22.0 (0.90), *y* = 9.94*x*–54.8 (0.94), and *y* = 7.31*x*–26.3 (0.93); for residues: *y* = 14.74*x*–49.5 (0.80), *y* = 27.08*x*–125.4 (0.90), and *y* = 17.57*x*–62.4 (0.86).

### Distribution of Cavity Volume

**Figure 3:**
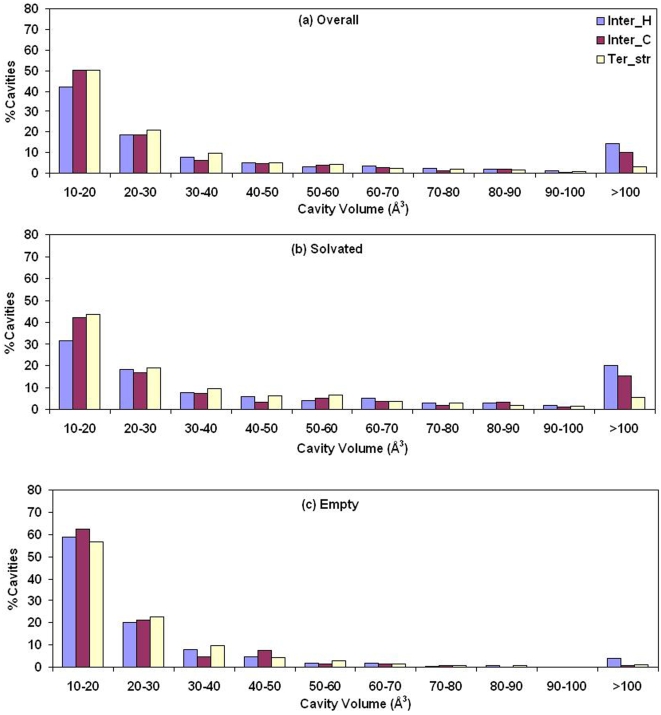
Distribution of the volume of cavities. Histogram of the volume of cavities (A) all taken together, and separated into (B) solvated and (C) empty cavities.

**Figure 4:**
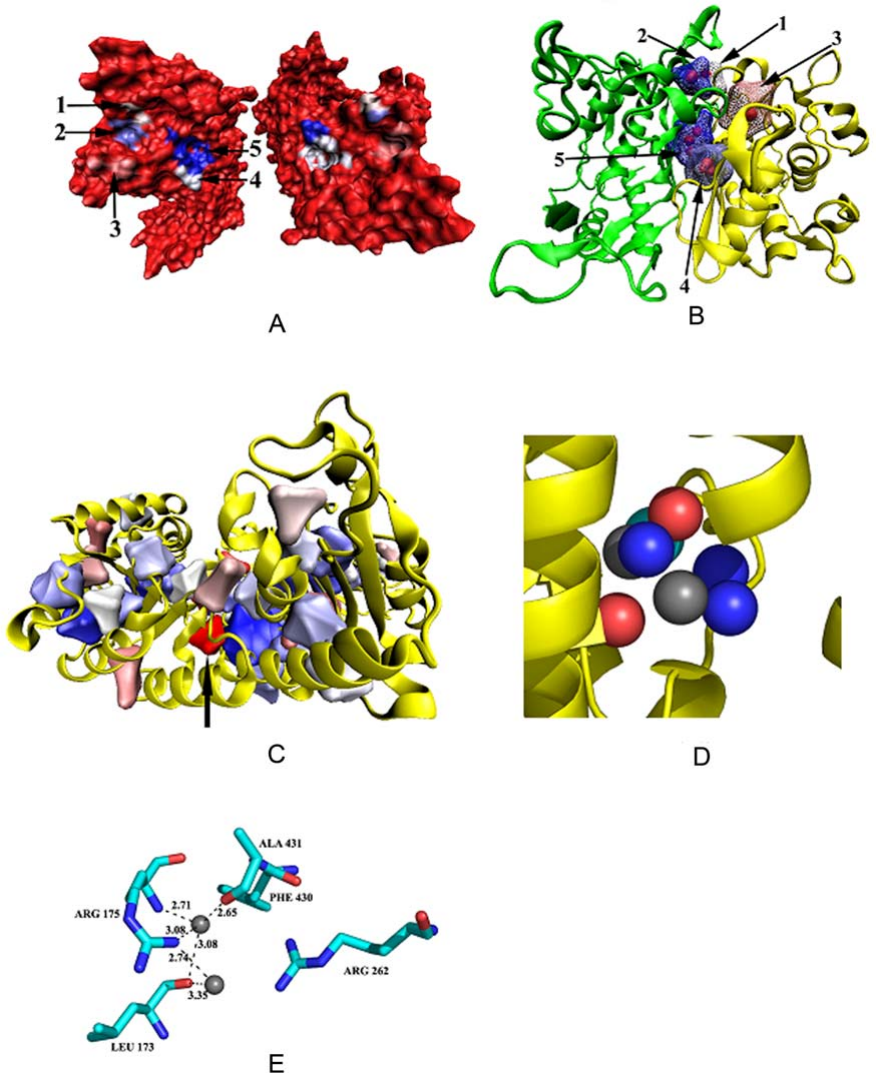
Visualization of different features in some cavities. (A) Surface representation of the structure with the PDB code, 1bkp, with five interface cavities. The two subunits are rotated about a vertical axis and taken apart to show how cavities are formed between them. (B) The cartoon representation of the secondary structures for the same protein as in (A), along with the maze representation of the cavities (with the enclosed water molecules in red). The individual cavities are labeled and their volume (Å^3^), number of water molecules and Rvs are as follows. 1: 12.3, 1, 0.99; 2: 13.3, 1, 0.99; 3: 31.7, 1, 0.75; 4: 110.5, 3, 0.84; and 5: 111.6, 3, 0.83. (C) Ter_str cavities within one subunit of the dimeric molecule, 1dpg. One of the cavities (pointed by an arrow, volume: 20.7 Å^3^ and Rvs: 0.92) with two water molecules is shown in (D) along with the CL atoms, and all the hydrogen bonds are given in (E). Diagrams (C–E) do not have the molecules in the same orientation. In (D) and (E) the water molecules are in grey.

The histogram of the distribution of volume in three different classes of cavities is shown in Figure 3A. Interfaces contain higher percentage of larger cavities (14.3% Inter_H and 10.2% of Inter_C with volume>100 Å^3^) than tertiary structure. The cavities were further divided into empty and solvated cavities and the distribution of their volume (Figure 3B and 3C) indicates that the large cavities (volume>50 Å^3^) are usually solvated. 50% of all Ter_str cavities are solvated, the corresponding values for Inter_H and Inter_C being 61% and 62%, respectively. The percentages calculated based on volume are 61, 83, and 79%, respectively for the above three categories. Examples of water molecules in cavities belonging to the tertiary structure and interface can be seen in Figure 4B–D.

### Cavity Shape

**Figure 5:**
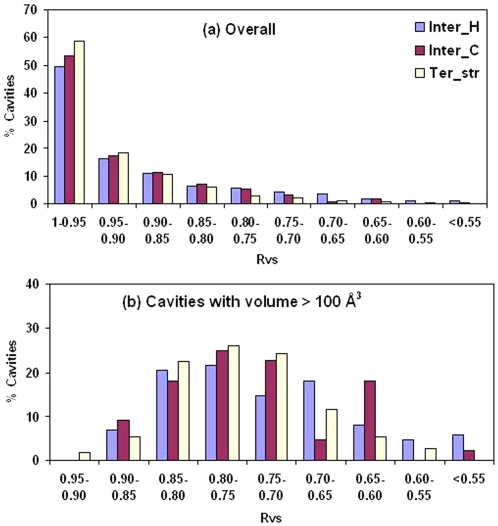
Distribution of Rvs. Histogram of Rvs for (A) all the cavities and (B) cavities with volume>100 Å^3^.

Rvs (defined in Methods) indicates how spherical a cavity is—for a perfect sphere the value would be 1.0, and would reduce in value as the cavity deviates from being spherical. The distribution of Rvs (Figure 5A) indicates that more than 50% of all cavities are nearly spherical. To investigate if the aspherical shape of the cavity can result from the size we plotted the distribution for the cavities having volume greater than 100 Å^3^ (Figure 5B). A peak near 0.75 indicates that such cavities are quite irregular in shape. Two small, spherical cavities (labels: 1 and 2) are illustrated in Figure 4A and 4B, in which the largest cavity (label, 5) deviates from the spherical shape.

### Amino Acid Preferences

**Figure 6:**
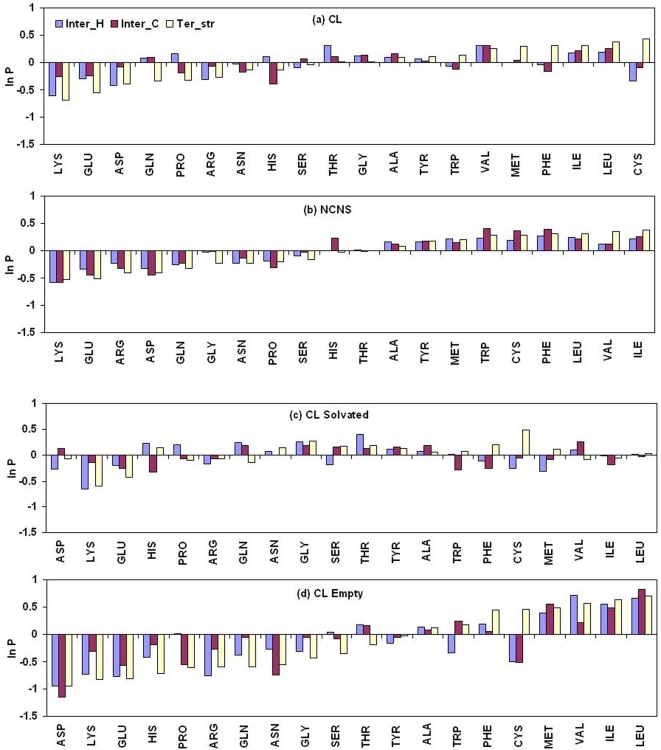
Propensities of residues to be associated with cavities. Propensities of different types of residues in (A) cavity-lining and (B) non-cavity-non-surface regions, and in the cavity-lining region of (C) solvated and (D) empty cavities in protein structures and interfaces.

As cavities are embedded within the protein structure (or interface) we compared the distribution of the CL residue types with that observed over all the proteins (or interfaces). Amino acid preferences for the CL and NCNS (non-cavity-non-surface) regions across all the three classes are shown in Figure 6A and 6B. A large, positive (or negative) value indicates preference (or avoidance), and a value close to zero suggests an occurrence close to the general population. Charged residues (Lys, Glu, Asp, and Arg) are avoided in general. Ter_str cavities prefer hydrophobic residues, such as Cys, Leu, Ile, Phe, Met, and Val. The preference for the branched aliphatic side chains seems to be the common feature for all the categories of cavities. However, in contrast to Ter_str, interface cavities avoid Cys, Phe and Trp, and prefer Thr, Gln, Gly—possibly due to a higher percentage of interface cavities being solvated. Unlike the CL region the trend in propensities is quite similar in the NCNS region in both types of interfaces and tertiary structure.

The amino acid preference for solvated and empty cavities across all three cavity classes is shown in Figure 6C and 6D. Residues which are preferred in empty cavities are Leu, Ile, Met, Phe, and Val, while Gly, Thr, and Tyr are more preferred in solvated cavities. Additionally, Ser is also found in greater number in the solvated cavities of interfaces in heterocomplexes, and His in those of homodimers.

### Atom Type Preferences

**Figure 7:**
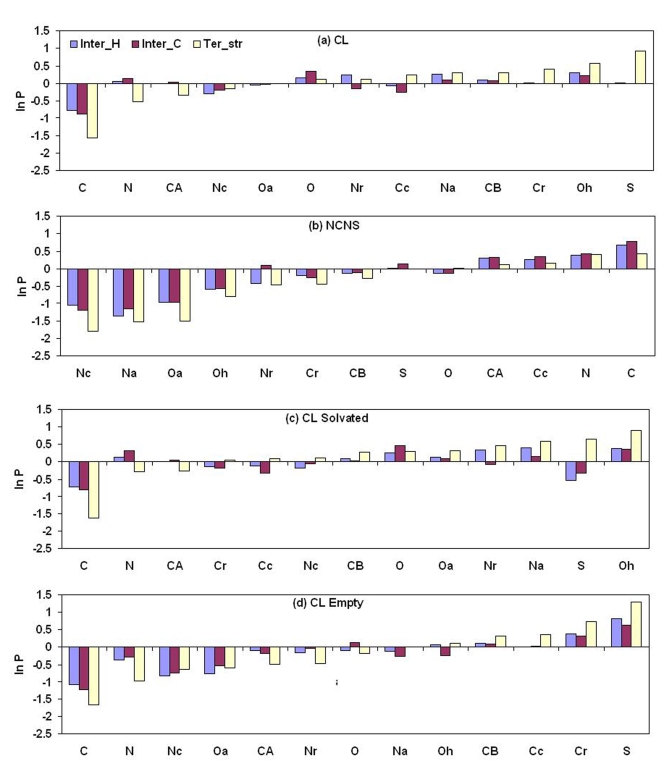
Propensities of atoms to be associated with cavities. Propensities of different types of atoms in (A) cavity-lining and (B) non-cavity-non-surface regions, and in the cavity-lining region of (C) solvated and (D) empty cavities in protein structures and interfaces. The atom types have been defined in Methods.

The propensities of different atom types to occur in the CL, NCNS regions, solvated and empty cavities in the three cavity classes are shown in Figure 7. There is a distinct pattern in the atom preference for Ter_str cavities—main-chain atoms (C, N, and CA) are disfavored and the side-chain atoms (aromatic carbon, hydroxyl oxygen and amide nitrogen) are favored in CL as compared to NCNS regions. In the NCNS region all three categories exhibit similar features, for example, polar side-chain atoms (Oa, Oh, Na and Nc) are not preferred and the preference is for C, N, CA and aliphatic carbon (Cc). Polar atoms like oxygen and nitrogen are more preferred in solvated cavities than empty cavities (Figure 7C and 7D). Figure 4D illustrates a cavity with two water molecules, and out of six CL atoms 2 are oxygen and 3 nitrogen.

### Distribution of Water Molecules in Cavities

**Figure 8:**
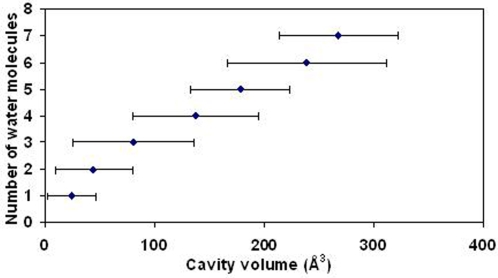
Plot of the number of water molecules present in a cavity against its volume (Å^3^). For a given value along the *y* axis the average of all the values along the *x* axis is shown (the horizontal bars representing the standard deviations). The numbers of points used for averaging are 948, 321, 83, 26, 10, 5 and 3 (six cavities have >7 water molecules). The equation for the least-squares line is *y* = 0.023*x*+0.70 (*R* ^2^ = 0.99).

There is a considerable variation in the cavity volume as a function of the number of water molecules contained in it. To discern any underlying trend we considered the Ter_str cavities, averaged the cavity volume containing a particular number of the solvent molecule (Figure 8) and based on the average numbers one can derive a linear relationship. Roughly, one water molecule can be accommodated in a volume of 15 Å^3^ (observed value), and an increment of ∼40–45 Å^3^ is needed for each additional molecule. On average a water molecule participates in 3.4 hydrogen bonds (the number includes those to other water molecules also; if hydrogen bonds to only protein atoms are considered the number is 2.6 for Ter_str cavities and 2.3 for interfaces). 15 Å^3^ is about the smallest volume that can enclose a water molecule, and such a volume would need about 5 CL atoms (based on equations in Table 2). Figure 4D and 4E, however, provides an example where a rather small cavity had six CL atoms, which could enclose two water molecules that participated in 4 and 2 hydrogen bonds, respectively.

### Change in Voronoi Volume of Atoms in Interface Relative to Tertiary Structure

**Figure 9:**
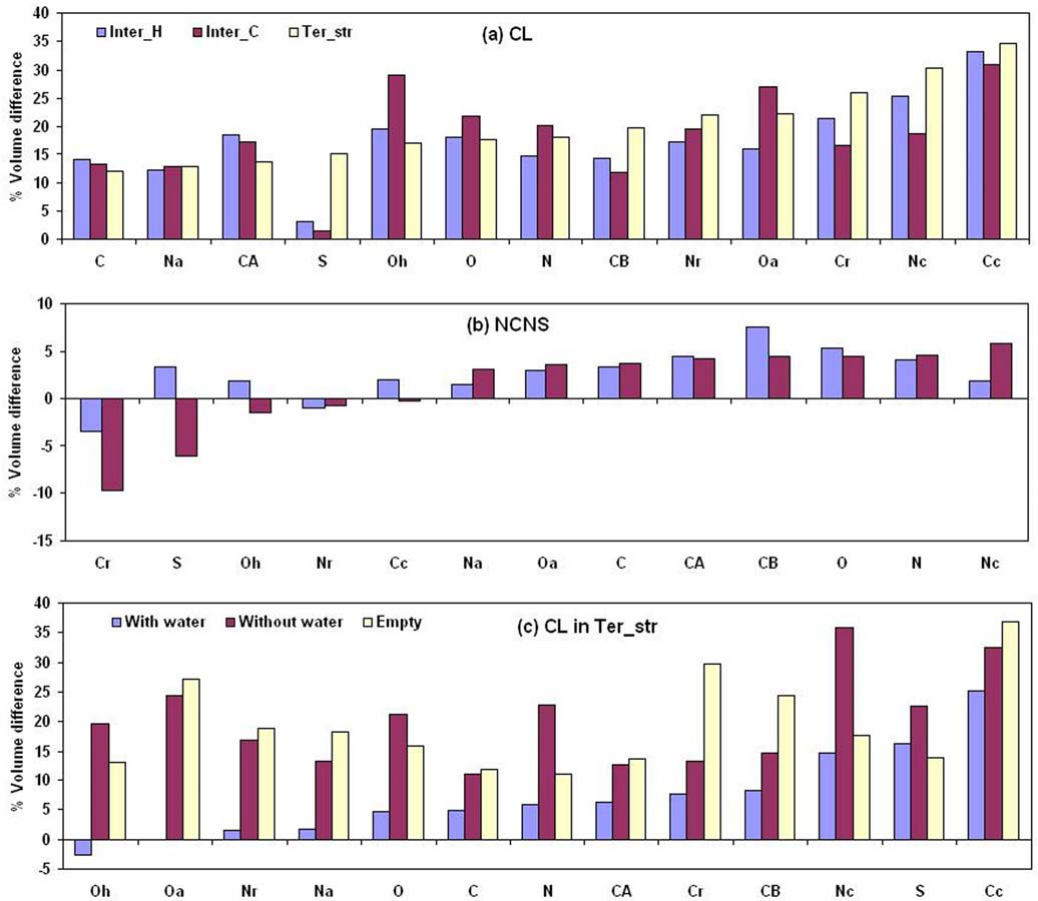
Change in the Voronoi volumes of atoms in cavities. Change in the Voronoi volumes (relative to those in the NCNS region of tertiary structure) of (A) CL atoms, (B) NCNS in interface, and (C) CL atoms in empty and solvated Ter_str cavities. For the solvated cavities in (C) the calculation has been done twice, once including the coordinates of the water molecules and the other excluding.

We first compare the Voronoi volume of the NCNS atoms in the interface to those in the protein tertiary structure (values are provided in Table S3). Most of the 13 atom types show an increase of value in the interface, though the change is usually <5% (Figure 9B). It should be mentioned here that for simplification we have grouped atoms together, for example all the aromatic atoms as Cr. However, it is known [17] that there can be some variation between the volumes of these atoms within a given aromatic residue or between any two of them. As such the result would be affected by the atom composition in the datasets. Under these limitations, cases where the difference is more are worth mentioning. In complexes, S and aromatic atoms have smaller values in the interface, indicating that these are better packed relative to the tertiary structure. Fleming and Richards [4] observed that in protein structures Cys and aromatic residues are better packed than the aliphatic ones. It appears that these residues (containing atoms types Cr and S) are still better packed in interfaces. On the other hand, N of Lys and Arg are lesser packed. For homodimers, CB atoms that link the main chain to the functional part of the side chain are also packed less efficiently.

As expected, if we compare the CL atoms instead of the NCNS atoms, there is an increase in volume (11–35%) compared to the atoms in the tertiary structure (Figure 9A). From Figure 9C one can see the difference in the Voronoi volume of CL atoms in solvated cavities, calculated including and excluding water molecules (blue and red bars, respectively). The difference in the volume of the polar atoms is to the extent of 12–25% as compared to 5–7% by the non-polar atoms. However, on including water the values of the polar atoms come to within 5%, indicating that the cavity water molecules are located closer to these CL atoms. One would have expected the bars corresponding to the empty cavities should match with the ones calculated without considering waters for the solvated cavities. But this is not quite correct, as they tend to have different sizes (solvated ones are bigger) and the propensities of atom-types (for example, compare Cr in Figure 7C and 7D) to occur in them are also different.

### Secondary Structure Preferences for Cavity Lining Residues

**Figure 10:**
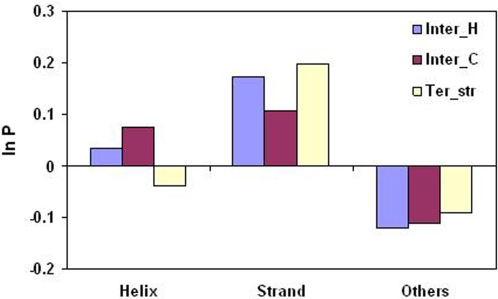
Propensity of cavity lining atoms to occur in different secondary structural elements.

**Figure 11:**
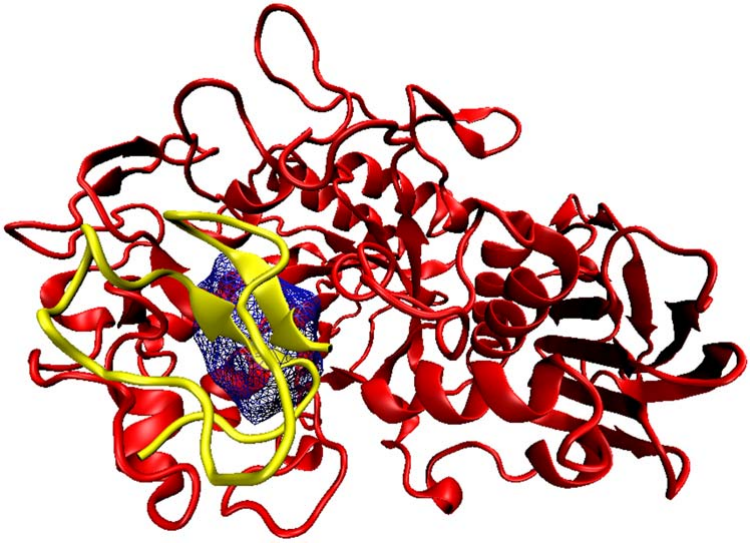
An example of a cavity involving β-sheets. The second largest cavity (with a volume of 222.4 Å^3^ and Rvs of 0.75) in the structure of 4htc, located in the interface, involving β-sheets. Of the 32 CL atoms, 4 are from helix, 14 from β-strands and 14 belong to ‘Others’.

**Figure 12:**
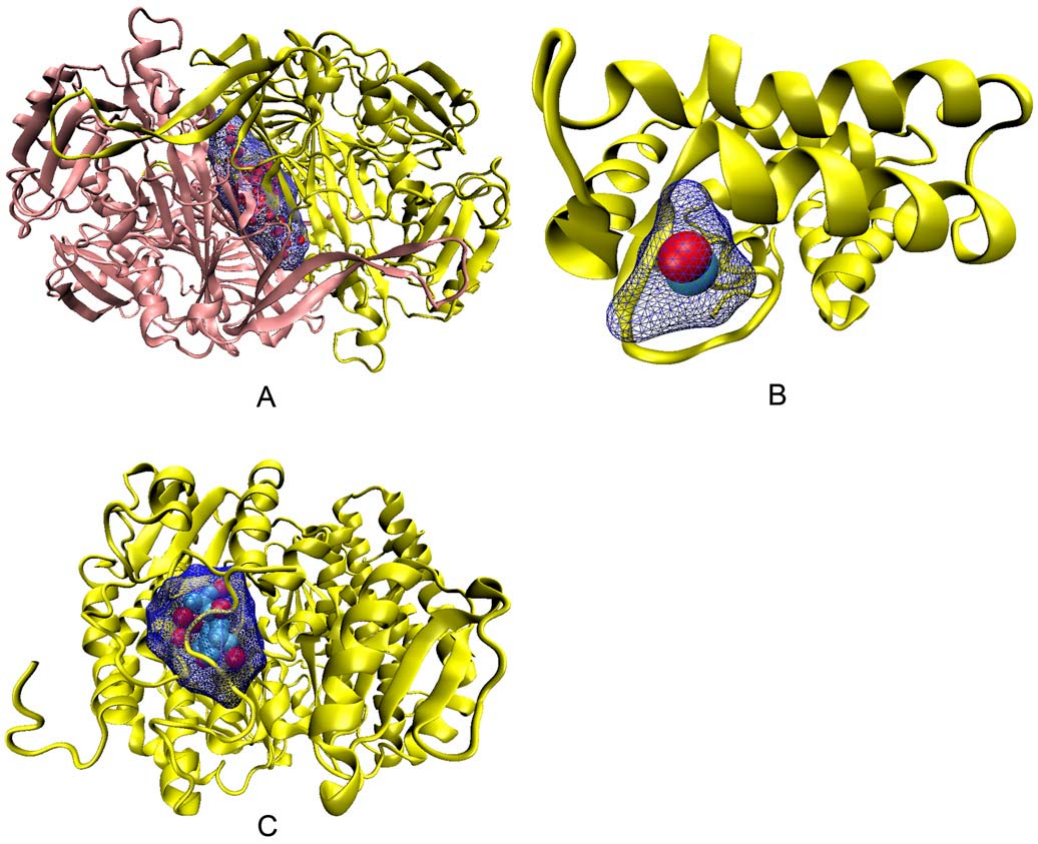
Some examples of cavities with water molecules, cation and ligand. The largest interface cavity (containing 56 water molecules, with a total volume of 2341 Å^3^ and Rvs of 0.5), in the homodimeric structure of 1oac is displayed in (A). Cavities with a large water content are also observed in dimeric interfaces: 1mor (46, 2202 Å^3^ and 0.5), 1chm (58, 1798 Å^3^, 0.3). PDB codes for cavities containing 10–20 water molecules are Ter_str (1cmb); Inter_H (1ade, 1b8j, 1bis, 1chm, 1mkb (2 cases), 1mor, 1oac, 1sox, 1utg and 5rub); Inter_C (1kz7). (B) shows a Ca^2+^ ion and a water (red) molecule in a cavity (volume: 17.2 Å^3^ and Rvs: 0.96) of 2scp. (C) shows α-d-glucose-6-phosphate and nine water molecules in a cavity (565.1 Å^3^ and 0.69) of 1mor.

The percentage composition of occurrence of CL atoms, as well as the ones in the whole data set, in three types of secondary structural elements is provided in Figure S3 and the propensities calculated from these numbers are shown in Figure 10. Strands are preferred in all three cavity classes, more so in Ter_str and Inter_H. Structures other than helices and strands are less inclined to form cavities. Two examples of cavities being located on top of β-sheets can be seen in Figures 11 and 12A. Even the structure shown in Figure 4C has 18 cavities (out of a total of 52) having more than 50% CL atoms coming from β-sheet. There is not much distinction between the cavity types based on the occurrence of the main- and side-chain atoms—Figure S4 indicates that when a helix or sheet contributes to a cavity, ∼70% of the atoms are from the side-chain; however, for ‘Others’ the value comes down to the range 56–63%.

## Discussion

### General Features of Cavities and Dependence on Protein/Interface Size

Hubbard & Argos [27] analyzed three classes of cavities: within domains, between domains and between protein subunits. Ter_str cavities considered here would include the first two classes, whereas the interfaces between subunits in obligate homodimers and protein-protein heterocomplexes substituted the last class. Interdomain and intersubunit cavities were on average found to be larger than those located within domains [27]. Our results (Figure 3A) comparing cavities in the tertiary structures and interfaces indicate that interface cavities, especially Inter_H, are indeed larger. However, if we distinguish between intradomain and interdomain cavities (of the 219 individual subunits considered by us 158 were single domain proteins and the rest multidomain) there is not much difference in the distribution (Figure S2). It can be mentioned that cavities with atomic surface components arising from more than one domain were deemed to be interdomain in the earlier study; however, we used a more stringent criterion of having at least 20% of CL atoms coming from a different domain. Nevertheless, Figure 2A indicates that the total cavity volume is a function of the protein volume, irrespective of the number of domains present.

It has been reported that the wide cavities form 0.002–1.55% of the volume of a protein (in a dataset of 75 monomeric proteins); however, no quantitative relationship could be established linking the two [21]. A linear relationship was noted between the number of voids and pockets plotted against the number of residues in each protein, although the total pocket volume did not correlate well [26],[29], possibly because no distinction was made between single and multiple subunit proteins, the latter containing tunnels or holes of large size [26]. From our analysis we could derive a linear relationship between the total volume of the cavities present and the total protein volume (Figure 2A). From this one can derive that for two proteins of volume 30,000 and 50,000 Å^3^, the cavities will constitute 0.56 and 0.99% of the volume (the two values are 0.61 and 0.91, using the power law). The observed minimum and maximum values were 0.06 and 2.26%, respectively. Cavities are usually located close to the protein surface—considering the CL atoms, most of them belong to the surface of the molecule.

Cavities were found to cover 10% of a typical interface [27]. Comparing the number of CL atoms to the total (Table S1b) we find that 5.5% atoms of the tertiary structure and 13.8 and 10.5% of homodimeric and hetercomplex interfaces form cavities. That for a given number of atoms the interfaces have about twice the number of cavities as the tertiary structure can also be seen from Table 1.

### Water in Cavities

For some structures the resolution of the data may be rather low, or the quality of the electron density too poor for the bound water molecules to be seen. Figure S5 indicates that there is an increase in the number of solvated cavities as the resolution improves from 2.5 Å till about 1.8 Å. If the water molecule is partially or completely disordered it cannot be located. Even with high resolution data detection of water molecules in cavities, especially if they are mobile due to the hydrophobic nature of the cavity, is rather tricky by conventional crystallographic analysis that neglects low resolution data [30] and as such, the average number of water molecules obtained could be an underestimate. Nevertheless, the average number of hydrogen bonds involving water in the solvated cavities—the number is 2.6 with protein atoms, and 3.4 if hydrogen bonding with other water molecules is also included—matches with the typical value of 3 hydrogen bonds made by a buried water molecule reported in literature [20],[21],[31]. The cavity volume needed to enclose one water molecule is ∼15 Å^3^, however, each additional water requires an extra volume of ∼40–45 Å^3^ (Figure 8).

### Preference of Secondary Structural Elements in Cavities

The propensity of the secondary structural elements to be associated with cavities indicates that β-strands have a high tendency and the non-regular regions (‘Others’) are disfavored (Figure 10). Interestingly, the packing densities of residues in turns, helices and strands were found to be 0.794, 0.744, and 0.723, respectively [4], indicating the β-strands to be packed least efficiently, possibly due to the greater occurrence of cavities associated with them, examples of which can be seen in Figures 11 and 12A. Loops and turns with higher flexibility can adjust the structure locally to avoid/minimize any local packing defects.

It has been suggested that C^β^ atoms do not cover an antiparallel β-sheet by a tightly packed layer, leaving holes equivalent to the size of a methyl group or water molecule [32]. These holes are possibly not included in our analysis because of the volume cut-off used in the definition of cavities. Additionally, these would have had all the CL atoms residing on the β-sheet; however, the percentage of cavities exclusively lined by β-sheet atoms is very low (<5%). The higher involvement of β-sheet residues in lining the cavities may have implications for the energetics of interaction. It has been observed that for protein-protein interactions, those having interfaces mostly made up of β-sheet have, on average lower free energy of binding compared to those having α- or αβ (mixed) classes of interfaces (Guharoy and Chakrabarti, unpublished). This observation may be understood in terms of the lowest packing efficiency of interfacial β structures, leading to lower van der Waals contacts and therefore lower binding free energies as well.

### Interface Cavities and Water

Figure 4A shows that when there is a surface groove that is not matched by a bulge on the surface of the interacting protein this would result in the formation of a cavity in the interface. Water molecules in the groove cannot be squeezed out and remains trapped inside the interface. When we analyzed if the water molecules can have direct hydrogen bond contact with both the subunits (Table 3) we observed that such molecules are just 37% and 51% in Inter_H and Inter_C, respectively, a smaller number (10% and 5%) of water molecules do not form any bond with either subunit. Indeed, one can see from Figure 12A, where the cavity can be considered as a casket of water molecules, the majority of which form hydrogen bonds between themselves. Even when the cavities contain one or two water molecules, the large size of the cavity may preclude the solvent molecules to interact with both the protein components. However, if we consider contacts (instead of hydrogen bonds) made with both the sides, a greater number (72% and 84%) of water molecules bridge the two subunits.

**Table 3:** Solvated cavities in interface and their hydrogen-bonding pattern.

	Interface Type	
	Inter_H	Inter_C
No. of solvated cavities	361	215
No. of water molecules	896	425
No. with HB (both)^a^	334	217
HB (single)	471	185
HB (none)	91	23

The labels, given in the first column, indicate the number of water molecules that are hydrogen bonded to both the subunits across the interface, or to just one of them, or none at all.

a Considering the contacts within 4.0 Å (not necessarily hydrogen bonds) from both the sides, the numbers are 645 and 358, respectively.

For the water molecules having direct hydrogen bonding with both the protein subunits we considered the involvement of main- and side-chain atoms and how important the solvents are in neutralizing the destabilizing effect of like-charges from the two subunits coming close to each other. It appears from Table 4 that water molecules sitting between like and opposite charges (in the side chain) occur to similar extent in Inter_H, but these are in 3∶4 ratio in Inter_C. A residue close to the two-fold axis in homodimeric interfaces can be in contact with the same residue from the other subunit – the so-called self contacts [33], which may explain some of the occurrences of like charges around water molecules in Inter_H.

**Table 4:** Number of water molecules mediating interaction between residues with opposite or like charges across the interface.

		Interface Type	
		Inter_H	Inter_C
(a) Residues are of the opposite charges	sc-sc	68	54
	mc-mc	71	39
	mc-sc	134	99
(b) Residues are of the same charge	sc-sc	69	41
	mc-mc	77	37
	mc-sc	82	68

sc and mc correspond to the side chain and the main chain, respectively. All the possible pairs of hydrogen bond interactions are considered (so that a given water molecule can contribute to more than one category of interactions). As such the total number of cases under Inter_H and Inter_C (501 and 338) exceeds the number (334 and 217) given in Table 3.

### Voronoi Volume and Packing of Atoms in Interface

The packing density at interfaces has been computed by comparing the Voronoi volume of the buried atoms in the interface to the reference atomic volume [34]. Such a plot is shown in Figure S6, which also includes the distribution for the atoms in the tertiary structure in individual files—as a reference for the normal distribution. When Vr is larger than unity it indicates that the packing density at interfaces is lower than that in protein interiors, and a smaller value indicates a higher density. The average values of Vr for the interfaces in homodimers and heretocomplexes are slightly higher than 1.01(±0.06) reported in [34] for heterocomplex interfaces. Overall, the volumes of the interface atoms are within 3% of those in the protein interior.

### Cavities Containing Ligand or Cofactor Molecules

The existence of any small molecule, other than water, in the cavities was found out (Table S4). In about 30% cases only 1–3 atoms of a much larger ligand are found to be inside the cavity, which are usually <20 Å^3^ in volume. These cavities cannot be considered as having a small molecule entrapped. Heterocomplex interfaces have just two cases where molecules used in the crystallization procedure found their way into the cavity. In general, biologically relevant molecules are not found in interface cavities—only two cases of cofactor molecules are found in Inter_H cavities. In the tertiary structure, there is an example of Mg ion being located in a volume of 16 Å^3^; cavities having Ca ion usually have a volume in the range of 17–18 Å^3^ (one example is shown in Figure 12B) and a K ion is observed in 20 Å^3^. Metals such as Hg, Ni, and iron-sulfur clusters occupy a much larger volume. Water molecules usually accompany the ligand in the cavity; the largest of such a cavity is displayed in Figure 12C.

In summary, in this work we have delineated the total volume expected to be occupied by cavities in a protein or a protein-protein interface of a particular size. A quantitative relationship has been derived for the volume of a cavity and the atoms/residues lining it. Of the secondary structural elements, β-strands have a higher inclination to be associated with cavities. For a comparable ensemble of atoms the interfaces contain about twice the number of cavities relative to the tertiary structure. It has been shown recently that a cavity of an appropriate size is the basis of peptidyl-prolyl-isomerase (PPIase) activity of an important class of enzymes (human FK506-binding protein 12) and that it is possible to create artificial PPIase activity by introducing such a cavity on barnase, a bacterial nuclease [35]. A comprehensive understanding of the features of cavities in protein interiors and interfaces, as presented here, would facilitate such protein design experiments.

## Methods

### Datasets

Atomic coordinates of the proteins were extracted from the Protein Data Bank (PDB) [36]. The dataset consisted of 97 monomeric proteins [13], 122 homodimers [8] and183 protein-protein complexes [37], mostly determined to a resolution of 2.5 Å or better (only16 structures are in the resolution range 2.5–3.0 Å). 219 independent subunits from the first two categories were used to identify cavities in the tertiary structure. The atoms that lose at least 0.1 Å^2^ of the accessible surface area (ASA) in the complex/dimer structure as compared to that in the isolated subunit were considered as interface atoms [7],[8]. The calculation of protein volume was done by ProGeom, based on the Alpha-Shape theory (server: http://nook.cs.ucdavis.edu/koehl/ProShape/download.html).

### Identification and Classification of Cavities

Quite a few algorithms/softwares exist for the calculation of cavities—VOIDOO [38], MS package [39],[40], VOLBL [41],[42], CAST [29] (now rechristened as CASTp), a Monte Carlo (MC) procedure [43], etc. Of these the last two performed in a more consistent way [43]. For our work the cavities for each protein are identified using the CASTp (Computed Atlas of Surface Topography of proteins) server [44] located at http://sts.bioengr.uic.edu/castp/. The basic ingredients of computational geometry applied in CASTp are: Delaunay triangulation, alpha shape, and discrete flow [45]–[48]. CASTp provides a full description of protein pockets and cavities, including volume, surface area, protein atoms that line the concavity, and features of pocket mouth(s) including identification of mouth atoms as well as measurement of mouth area and circumference. The default probe radius of 1.4 Å has been used for our calculations.

Three classes of cavities were identified: (a) Ter_str (cavities in monomeric proteins and within one subunit of homodimeric proteins); (b) Inter_H (those within homodimer interfaces); and (c) Inter_C (within protein-protein complex interfaces).

Surface pockets and cavities belonging to the subunit or interface are illustrated in Figure 1. Any residue contributing one or more atoms to the cavity-lining (CL) region is considered as a CL residue; the same is true for the NCNS (non-cavity-non-surface) region. Interface cavities should have at least 20% of the cavity-lining atoms from a different subunit. The same condition was also used to identify if any Ter_str cavity belonged to the interdomain region, after identifying the individual domain residues from SCOP [49]. As we have used the option in CASTp that defines cavities based on molecular surface (rather than ASA), a few atoms not identified by us as belonging to the interface were also found lining the interface cavities and these were counted as being associated with the cavity (as well as the interface). Only the cavities with volume>11.5 Å^3^ (the volume of a probe with radius 1.4 Å) were retained for analysis. Further the cavities were classified as solvated or empty based on the presence or the absence of crystallographically determined water molecules in them. Cavities were considered for the existence of embedded water molecules starting from the smallest one. When two cavities, one small and the other large and irregular have some common CL atoms, there could be ambiguity is assigning a water molecule that may lie close to the shared atom(s). As such the cavities were considered in the ascending order of volume. The location of water in a cavity was found out as follows. (i) It has to be within 4.5 Å of a CL atom. (ii) If such water exists, all CL atoms within 4.5 Å from the water are found. (iii) If the distance from the center of mass of the cavity to the water molecule is less than that to any of the CL atoms in contact (as obtained in ii), the water is assumed to belong to the cavity. The existence of any ligand in a cavity was found in a similar fashion. Hydrogen bonding involving a water molecule (to protein atoms, as well as to other water molecules in the cavity) was determined using HBPLUS [50]. The surface representation of the cavities was made using MSMS [51] and displayed with VMD [52].

### Atom Types

Based on chemical characteristics the atoms in the PDB files were grouped into thirteen classes. Following are the atom labels (and their definition). N, CA, C, O, CB, S (sulfur of Met and Cys), Oh (the hydroxyl group of Ser, Thr and Tyr), Oa (both the carboxylate oxygen atoms of Asp and Glu, and the amide oxygen of Asn and Gln), Na (the amide nitrogen of Asn and Gln), Nc (side-chain N atoms of Lys and Arg), Nr (ring N atoms of His and Trp), Cr (aromatic C atoms of Phe, Tyr, His and Trp), Cc (aliphatic C atoms excluding CB of Val, Ile, Leu, Met, Lys, Pro, Gln, Glu, Arg and Thr). In the first 5 cases the labels are the same as the atomic labels used in PDB.

### Identification and Comparison of Atom Volume

The Voronoi [53] procedure for the determination of volume of atomic groups was applied to proteins by Richards [2]. By constructing the minimally sized polyhedron (called a Voronoi polyhedron) around each atom, this procedure allocates the space within a structure, to its constituent atoms. The original program, as modified and extended by Harpaz et al. [54] and Voss et al. [55] (available at http://www.molmovdb.org/geometry/ [17]), has been used in this study. Two parameters need to be provided for the program – the atomic van der Waals radii and Voronoi plane positioning method (method B used).

### Propensity

The propensity of a residue to be in the CL region is given as ln *P*, where
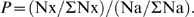
Nx is the number of atoms of amino acid residue of type X lining the cavities and ∑Nx is its total number in the dataset (consisting of all the subunits for Ter_str, and all the interfaces, for Inter_H and Inter_C); Na and ∑Na are the corresponding numbers considering all residue types together. This method is based on counting the atoms, rather than residues, as it is supposed to provide values that are independent of the size of the residue [56]. The propensity was also calculated in a similar fashion considering different types of atoms (instead of residues), as also for the occurrence of secondary structural elements (helix, strand and the rest, termed ‘Others’) lining the cavities. Secondary structure assignments were made using the DSSP program [57].

### Rvs Calculation

Rvs provides an estimate of the surface:volume ratio for a cavity relative to that for a sphere having the same volume as the cavity. The following formula is used for its calculation:
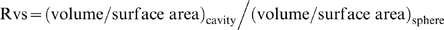



## Supporting Information

Figure S1 Plot of the individual cavity volume against the number of cavity lining atoms (and residues). The correlation coefficient, *r*, is given in parentheses.
(4.09 MB DOC)

Figure S2 Distribution of volume of cavities - intradomain and interdomain. In the 932 cavities located in 45 structures considered, 17% belong to interdomain region.
(1.31 MB DOC)

Figure S3 Percentage composition of secondary structural elements (A) for the CL atoms in three cavity classes and (B) for all atoms in the dataset.
(1.83 MB DOC)

Figure S4 Percentage distribution between main- and side-chain groups of CL atoms located in three secondary structural elements (helix, strand, and others).
(1.53 MB DOC)

Figure S5 Plot of the percentage of solvated cavities (both in terms of number and the total volume of cavities) as a function of resolution of the X-ray structure.
(3.10 MB DOC)

Figure S6 Histogram of Vr (Vr = 1/N Σ_N = 0_ V/V_o_), N is the number of atom types, usually 13, present in a structure; V is the average value of the Voronoi volume of a given atom type in the tertiary structure or the interface corresponding to a given PDB file and V_o_ is the value for the same atom in the interior of the tertiary structure calculated for the whole dataset (given in Table S3C). Only the interfaces that have more than 100 fully-buried atoms (in the two components taken together) have been included (72 and 25 cases of homodimers and heterocomplexes, respectively); all NCNS and CL atoms contribute to V. The average values of Vr are 1.03(±0.04), 1.03(±0.04) and 1.00(±0.03) for interfaces in homodimers and heterocomplexes, and in protein interior.
(1.52 MB DOC)

Table S1 Statistics on the cavities identified in different datasets (A) and statistics on the count of different types of atoms pertaining to the definition of cavities (B) (Figure 1).
(0.03 MB DOC)

Table S2 Various parameters calculated for individual PDB entries.
(1.41 MB DOC)

Table S3 The number of occurrences and the average Voronoi volume (Å^3^) of the thirteen atom types in (A) the NCNS region and (B) three types of cavities, and (C) considering NCNS and CL atoms together for the tertiary structure.
(0.07 MB DOC)

Table S4 Details of the ligands present in cavities.
(0.11 MB DOC)
