# Supplementary material for: Cavities and Atomic Packing in Protein Structures and Interfaces
Source: PLoS Comput Biol. 2008 Sep 26;4(9):e1000188. doi: 10.1371/journal.pcbi.1000188 (PMC2582456; doi:10.1371/journal.pcbi.1000188)
Supplement: Figure S1 — Plot of the individual cavity volume against the number of cavity lining atoms (and residues). The correlation coefficient, r, is given in parentheses. (4.09 MB DOC) [file pcbi.1000188.s001.doc]

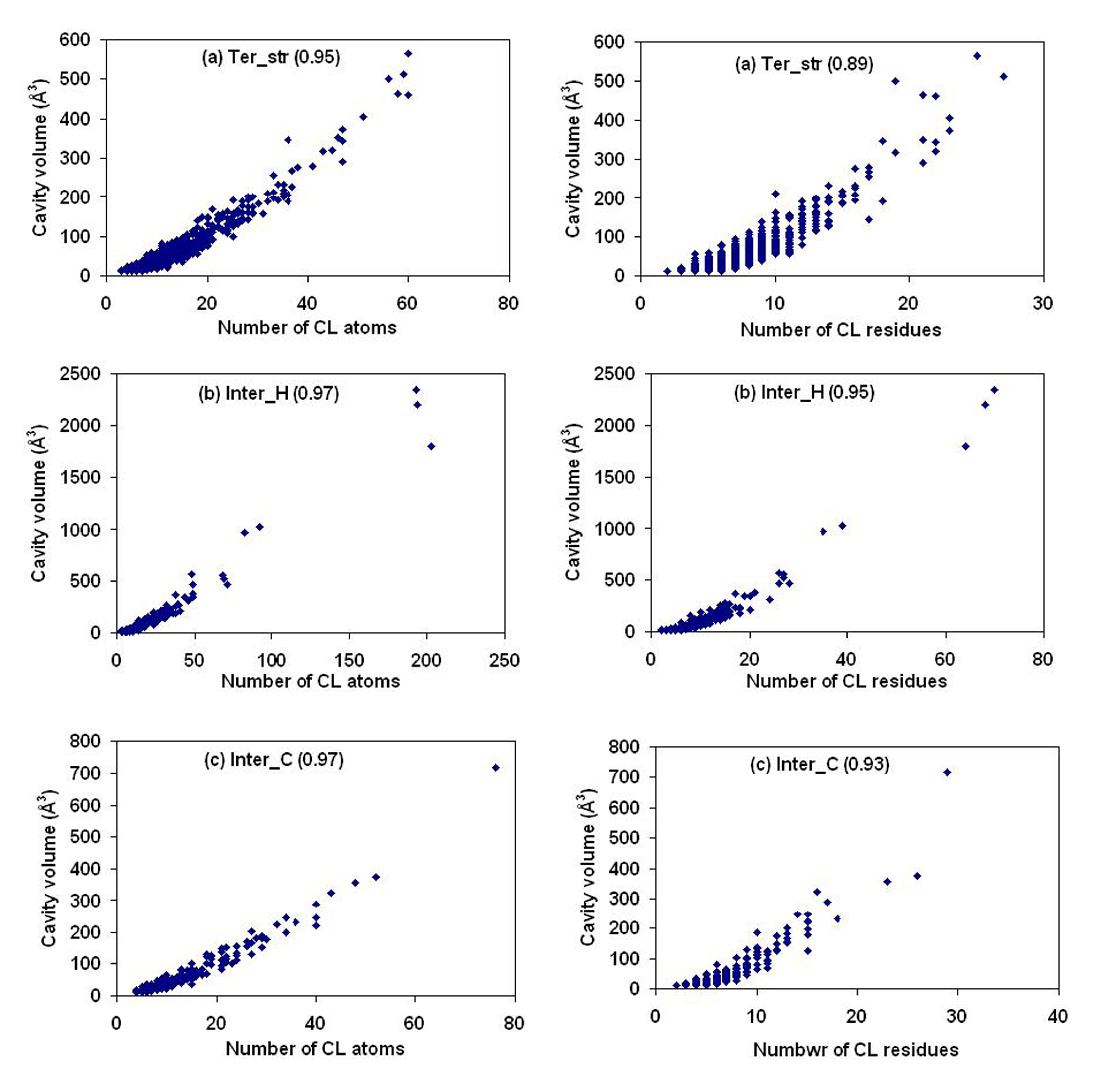


Figure S1. Plot of the individual cavity volume against the number of cavity lining atoms (and residues); the correlation coefficient, r, is given in parentheses.
