# Supplementary material for: Cavities and Atomic Packing in Protein Structures and Interfaces
Source: PLoS Comput Biol. 2008 Sep 26;4(9):e1000188. doi: 10.1371/journal.pcbi.1000188 (PMC2582456; doi:10.1371/journal.pcbi.1000188)
Supplement: Figure S2 — Distribution of volume of cavities - intradomain and interdomain. In the 932 cavities located in 45 structures considered, 17% belong to interdomain region. (1.31 MB DOC) [file pcbi.1000188.s002.doc]

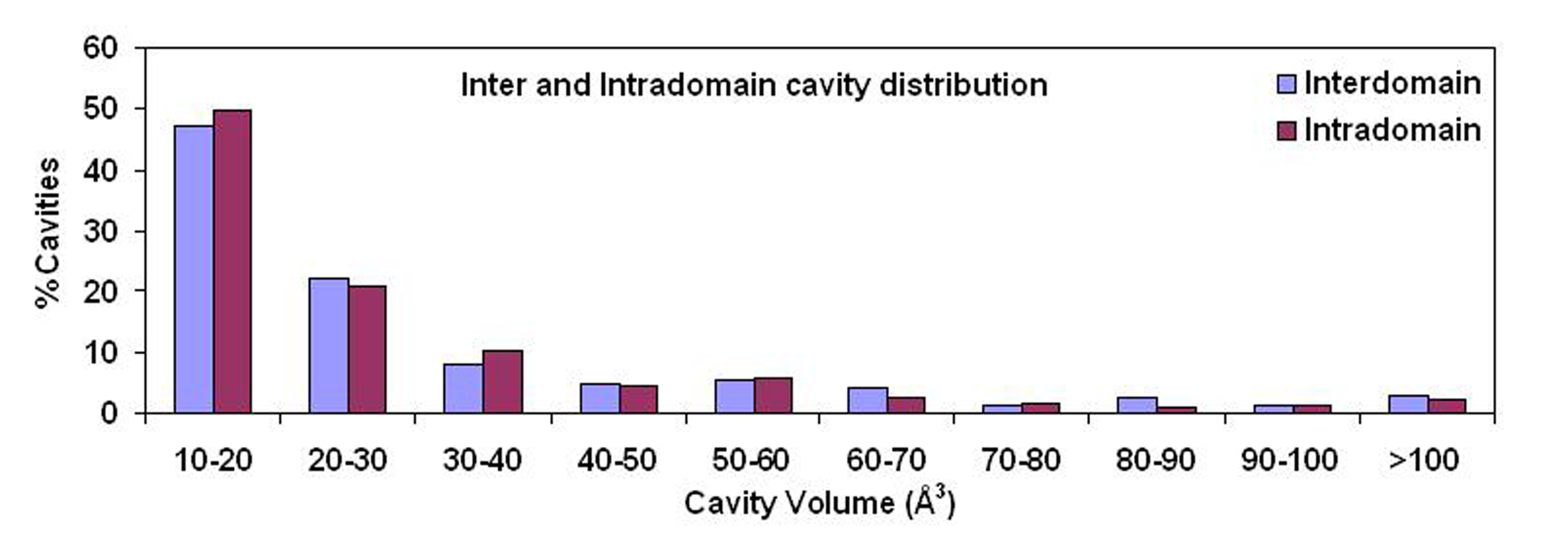


Figure S2. Distribution of volume of cavities – intradomain and interdomain. (In the 932 cavities located in 45 structures considered, 17% belong to interdomain region).
