# Supplementary material for: Cavities and Atomic Packing in Protein Structures and Interfaces
Source: PLoS Comput Biol. 2008 Sep 26;4(9):e1000188. doi: 10.1371/journal.pcbi.1000188 (PMC2582456; doi:10.1371/journal.pcbi.1000188)
Supplement: Figure S3 — Percentage composition of secondary structural elements (A) for the CL atoms in three cavity classes and (B) for all atoms in the dataset. (1.83 MB DOC) [file pcbi.1000188.s003.doc]

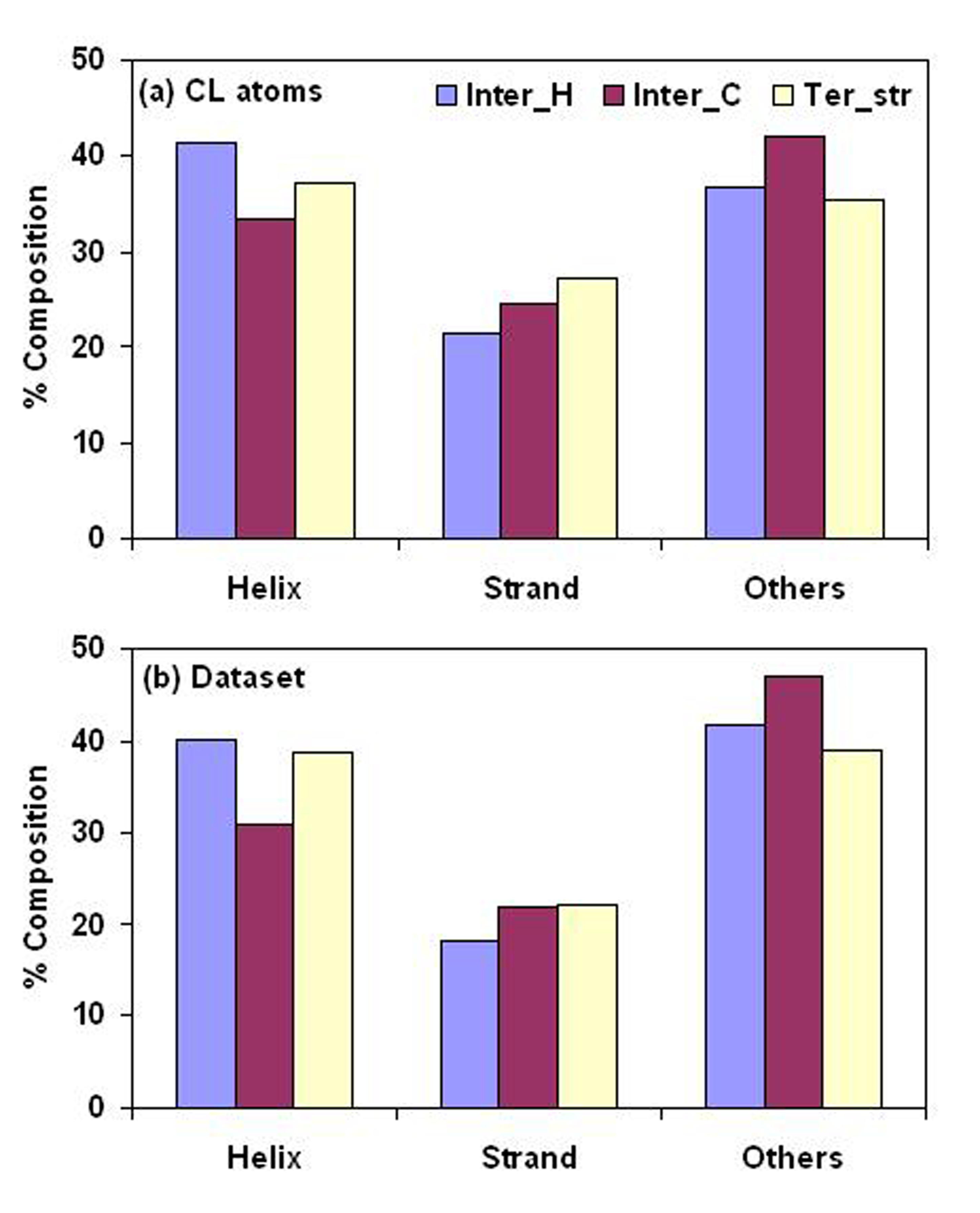


Figure S3. Percentage composition of secondary structural elements (A) for the CL atoms in three cavity classes; and (B) for all atoms in the dataset.
