# Supplementary material for: Cavities and Atomic Packing in Protein Structures and Interfaces
Source: PLoS Comput Biol. 2008 Sep 26;4(9):e1000188. doi: 10.1371/journal.pcbi.1000188 (PMC2582456; doi:10.1371/journal.pcbi.1000188)
Supplement: Figure S4 — Percentage distribution between main- and side-chain groups of CL atoms located in three secondary structural elements (helix, strand, and others). (1.53 MB DOC) [file pcbi.1000188.s004.doc]

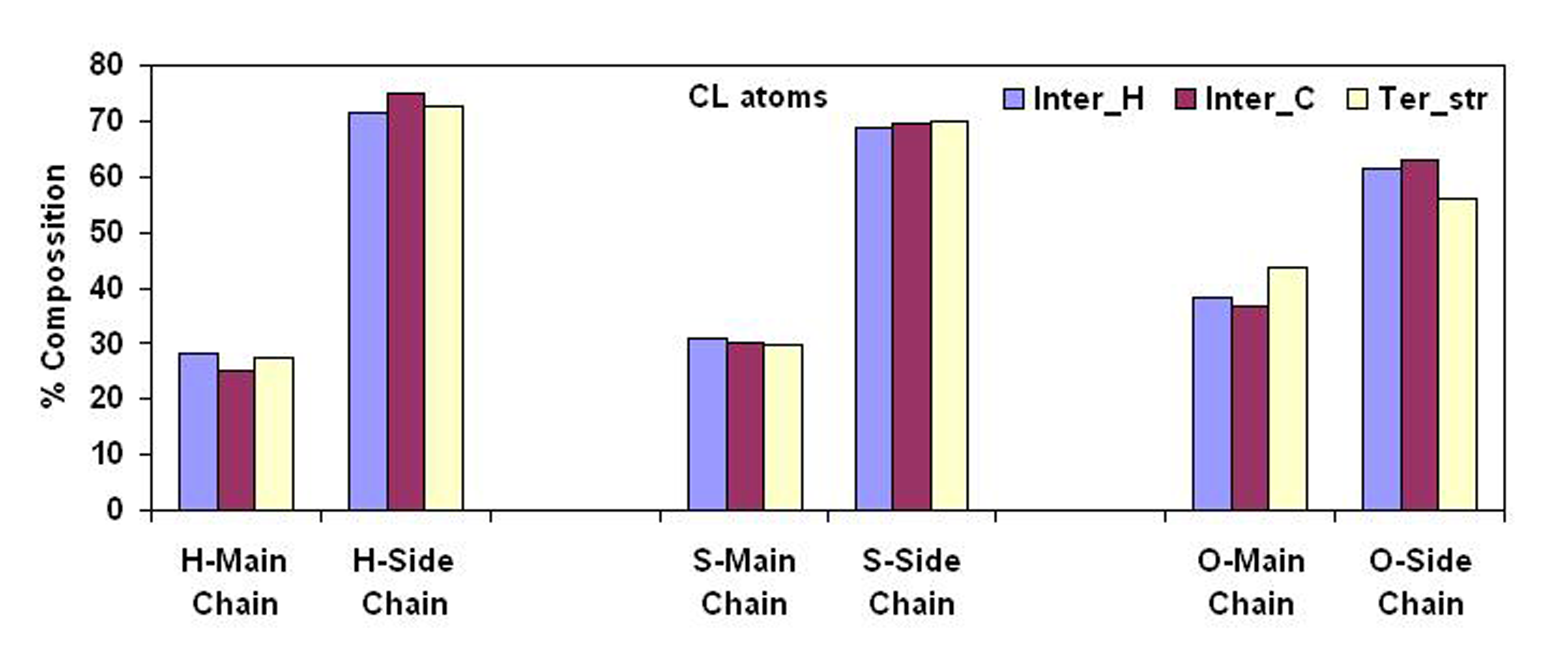


Figure S4. Percentage distribution between main- and side-chain groups of CL atoms located in three secondary structural elements (helix, strand and Others).
