# Supplementary material for: Cavities and Atomic Packing in Protein Structures and Interfaces
Source: PLoS Comput Biol. 2008 Sep 26;4(9):e1000188. doi: 10.1371/journal.pcbi.1000188 (PMC2582456; doi:10.1371/journal.pcbi.1000188)
Supplement: Figure S5 — Plot of the percentage of solvated cavities (both in terms of number and the total volume of cavities) as a function of resolution of the X-ray structure. (3.10 MB DOC) [file pcbi.1000188.s005.doc]

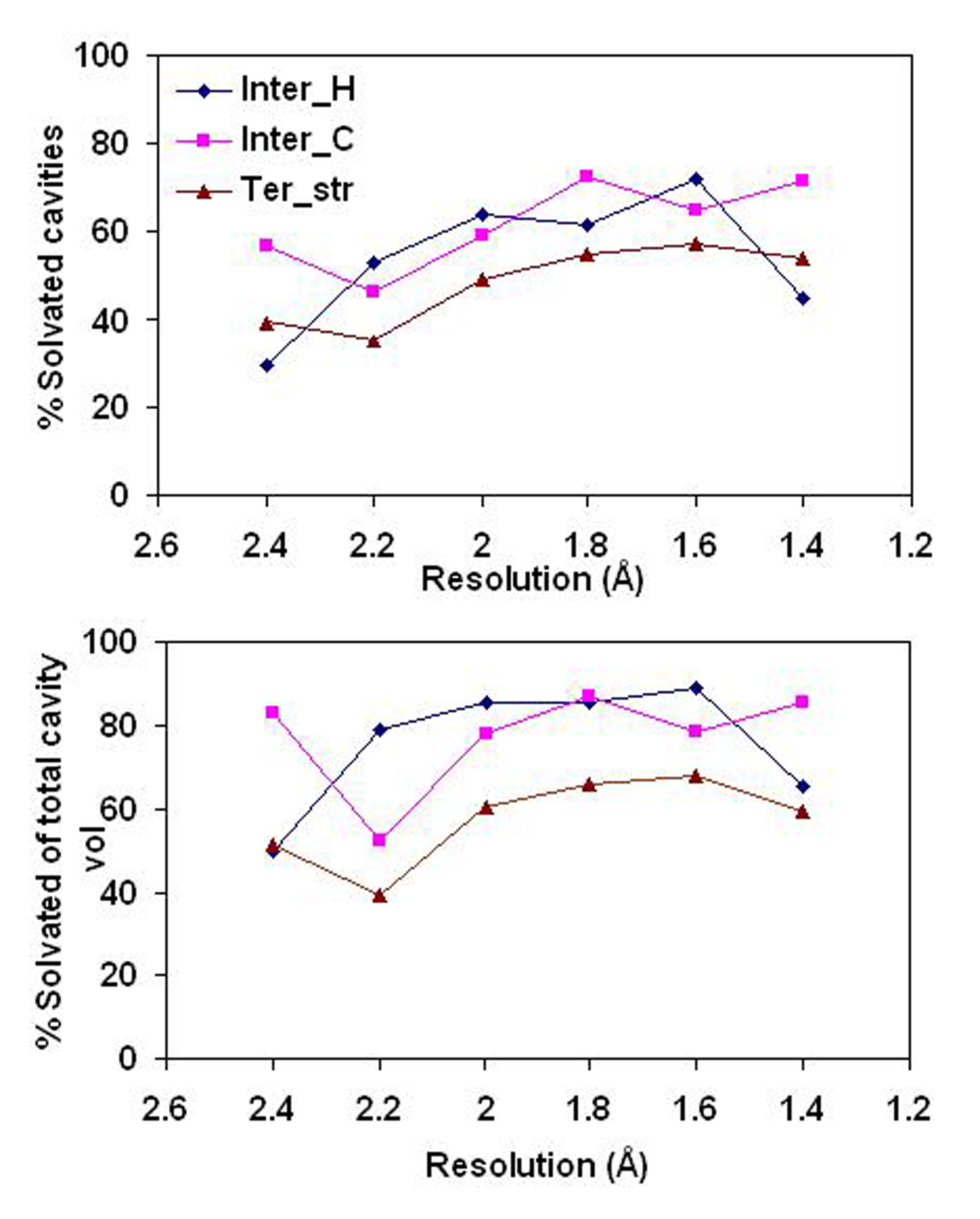


Number of cases

| Res | Inter_H | Inter_C | Ter_str |
| --- | --- | --- | --- |
| <1.5 | 10 | 11 | 15 |
| 1.5-1.7 | 13 | 19 | 21 |
| 1.7-1.9 | 38 | 42 | 67 |
| 1.9-2.1 | 32 | 34 | 50 |
| 2.1-2.3 | 14 | 31 | 22 |
| 2.3-2.5 | 6 | 15 | 10 |
| >2.5 | 1 | 1 | 4 |

Figure S5. Plot of the percentage of solvated cavities (both in terms of number and the total volume of cavities) as a function of resolution of the X-ray structure.
