# Supplementary material for: Cavities and Atomic Packing in Protein Structures and Interfaces
Source: PLoS Comput Biol. 2008 Sep 26;4(9):e1000188. doi: 10.1371/journal.pcbi.1000188 (PMC2582456; doi:10.1371/journal.pcbi.1000188)
Supplement: Figure S6 — Histogram of Vr (Vr = 1/N ΣN = 0 V/Vo), N is the number of atom types, usually 13, present in a structure; V is the average value of the Voronoi volume of a given atom type in the tertiary structure or the interface corresponding to a given PDB file and Vo is the value for the same atom in the interior of the tertiary structure calculated for the whole dataset (given in Table S3C). Only the interfaces that have more than 100 fully-buried atoms (in the two components taken together) have been included (72 and 25 cases of homodimers and heterocomplexes, respectively); all NCNS and CL atoms contribute to V. The average values of Vr are 1.03(±0.04), 1.03(±0.04) and 1.00(±0.03) for interfaces in homodimers and heterocomplexes, and in protein interior. (1.52 MB DOC) [file pcbi.1000188.s006.doc]

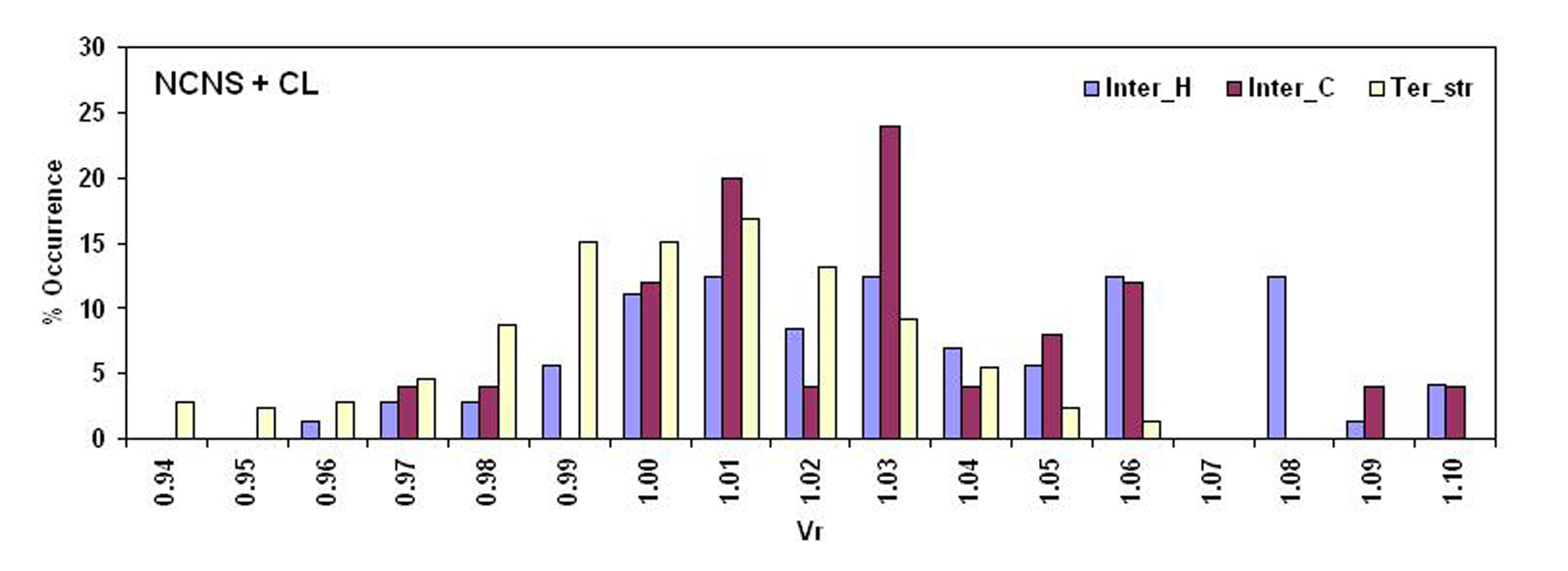


Figure S6. Histogram of Vr (Vr = 1/N ∑ V/V_o_), N is the number of atom types,

N=0

usually 13, present in a structure; V is the average value of the Voronoi volume of a given atom type in the tertiary structure or the interface corresponding to a given PDB file and V_o_ is the value for the same atom in the interior of the tertiary structure calculated for the whole dataset (given in Table S3C). Only the interfaces that have more than 100 fully-buried atoms (in the two components taken together) have been included (72 and 25 cases of homodimers and heterocomplexes, respectively); all NCNS and CL atoms contribute to V. The average values of Vr are 1.03(±0.04), 1.03(±0.04) and 1.00(±0.03) for interfaces in homodimers and heterocomplexes, and in protein interior.
