# Supplementary material for: Cavities and Atomic Packing in Protein Structures and Interfaces
Source: PLoS Comput Biol. 2008 Sep 26;4(9):e1000188. doi: 10.1371/journal.pcbi.1000188 (PMC2582456; doi:10.1371/journal.pcbi.1000188)
Supplement: Table S1 — Statistics on the cavities identified in different datasets (A) and statistics on the count of different types of atoms pertaining to the definition of cavities (B) (Figure 1). (0.03 MB DOC) [file pcbi.1000188.s007.doc]

Table S1.

(A) Statistics on the cavities identified in different datasets

| Dataset (number of files) | Number of cavities | | | | |
| --- | --- | --- | --- | --- | --- |
|  | In the whole structure | In Interface | Partially in interface^a^ | Not in interface | In one subunit & not in interface |
| Heterocomplex (183) | 3944 | 431 (215) **Inter_C** | 137 | 3513 | - |
| Homodimer (122) | 4438 | 615 (361)  **Inter_H** | 136 | 3823 | 1926^b^ (894)  **Ter_str** |
| Monomer (97) | 1458 | - | - | - | 1458 (508)  **Ter_str** |

A hyphen is used when a particular column heading is not applicable, or the feature is not used in the analysis. The names of different categories of cavities are given in bold against the box containing the data for the corresponding cavity. Both homodimers and monomers contribute to Ter_str. The numbers of solvated cavities are given in parentheses.

a When a cavity has at least 20% of the CL atoms from the other subunit it is considered to be an interface cavity. The number of cavities when the contribution is < 20% is provided in the column. These cavities are assumed to belong to a particular subunit and their number is also included in the next column.

b In some cases the two subunits are not exactly the same; as such the number is not exactly the half of the value given in the previous column.

(B) Statistics on the count of different types of atoms pertaining to the definition of cavities (Figure 1)

| Atoms | Tertiary structure | Interface | |
| --- | --- | --- | --- |
|  |  | Homodimer | Heterocomplex |
| Total^a^ | 450743 | 49908 | 36583 |
| CL | 24587 | 6872 | 3827 |
| NCNS | 184698 | 16806 | 10897 |

a The total number of atoms in the tertiary structure and the two types of interfaces.
