# Supplementary material for: Cavities and Atomic Packing in Protein Structures and Interfaces
Source: PLoS Comput Biol. 2008 Sep 26;4(9):e1000188. doi: 10.1371/journal.pcbi.1000188 (PMC2582456; doi:10.1371/journal.pcbi.1000188)
Supplement: Table S2 — Various parameters calculated for individual PDB entries. (1.41 MB DOC) [file pcbi.1000188.s008.doc]

Table S2. Various parameters calculated for individual PDB entries

| **PDB Code** | **Number of Atoms** | **Volume (Å^3^)** | | **# of cavities** | **Cavity volume (Å^3^) in the range** | | | | | | | | | | **Total number of cavity lining** | | **Packing Density** | |
| --- | --- | --- | --- | --- | --- | --- | --- | --- | --- | --- | --- | --- | --- | --- | --- | --- | --- | --- |
|  |  | **Protein** | **Total cavity** |  | **10-20** | **20-30** | **30-40** | **40-50** | **50-60** | **60-70** | **70-80** | **80-90** | **90-100** | **>100** | **Atoms** | **Residues** | **Buried atoms** | **Vr** |
| **(a) Tertiary structure** | | | | | | | | | | | | | | | | | | |
| 13PK | 3127 | 73190 | 594 | 24 | 15(4) | 3(0) | 1(0) | 1(0) | 2(2) | 2(2) | 0(0) | 0(0) | 0(0) | 0(0) | 147 | 95 | 1495 | 0.98 |
| 1A7V | 921 | 24123 | 63 | 4 | 4(1) | 0(0) | 0(0) | 0(0) | 0(0) | 0(0) | 0(0) | 0(0) | 0(0) | 0(0) | 21 | 16 | 297 | 1.01 |
| 1AG9 | 1385 | 32806 | 178 | 9 | 5(2) | 3(0) | 1(1) | 0(0) | 0(0) | 0(0) | 0(0) | 0(0) | 0(0) | 0(0) | 48 | 28 | 623 | 1 |
| 1AH0 | 2525 | 59026 | 397 | 17 | 11(6) | 3(3) | 1(1) | 0(0) | 1(1) | 1(1) | 0(0) | 0(0) | 0(0) | 0(0) | 106 | 78 | 1170 | 1.01 |
| 1AK1 | 2480 | 59334 | 357 | 17 | 12(4) | 2(0) | 1(0) | 0(0) | 1(1) | 1(1) | 0(0) | 0(0) | 0(0) | 0(0) | 96 | 61 | 1086 | 1.01 |
| 1AK2 | 1699 | 43563 | 159 | 10 | 8(1) | 2(0) | 0(0) | 0(0) | 0(0) | 0(0) | 0(0) | 0(0) | 0(0) | 0(0) | 46 | 34 | 612 | 0.99 |
| 1AMU | 3948 | 95184 | 1300 | 38 | 20(10) | 5(2) | 3(1) | 4(1) | 1(1) | 1(0) | 1(0) | 0(0) | 1(0) | 2(2) | 272 | 155 | 1694 | 1.02 |
| 1ATL | 1605 | 38704 | 252 | 9 | 5(3) | 2(0) | 1(0) | 0(0) | 0(0) | 0(0) | 0(0) | 0(0) | 0(0) | 1(0) | 56 | 35 | 738 | 1 |
| 1AW7 | 1562 | 39224 | 466 | 14 | 8(4) | 0(0) | 1(0) | 1(0) | 2(1) | 1(1) | 0(0) | 1(1) | 0(0) | 0(0) | 97 | 56 | 599 | 1.03 |
| 1AYL | 4109 | 97984 | 2152 | 57 | 27(11) | 9(8) | 5(4) | 4(4) | 2(2) | 4(3) | 1(1) | 1(0) | 0(0) | 4(4) | 441 | 223 | 1820 | 1.04 |
| 1B11 | 923 | 23656 | 64 | 3 | 2(1) | 1(0) | 0(0) | 0(0) | 0(0) | 0(0) | 0(0) | 0(0) | 0(0) | 0(0) | 18 | 11 | 318 | 1.02 |
| 1B12 | 1887 | 46608 | 211 | 9 | 5(3) | 1(1) | 2(2) | 1(0) | 0(0) | 0(0) | 0(0) | 0(0) | 0(0) | 0(0) | 60 | 36 | 781 | 0.99 |
| 1BC2 | 1672 | 40426 | 386 | 13 | 5(3) | 4(2) | 0(0) | 1(1) | 2(2) | 0(0) | 1(1) | 0(0) | 0(0) | 0(0) | 88 | 54 | 770 | 1 |
| 1BEA | 889 | 22140 | 13 | 1 | 1(0) | 0(0) | 0(0) | 0(0) | 0(0) | 0(0) | 0(0) | 0(0) | 0(0) | 0(0) | 4 | 4 | 337 | 0.99 |
| 1BIN | 1079 | 27365 | 48 | 3 | 3(0) | 0(0) | 0(0) | 0(0) | 0(0) | 0(0) | 0(0) | 0(0) | 0(0) | 0(0) | 16 | 13 | 410 | 1.03 |
| 1BKZ | 1058 | 26349 | 324 | 9 | 4(1) | 1(0) | 0(0) | 1(0) | 1(0) | 1(1) | 1(0) | 0(0) | 0(0) | 0(0) | 68 | 35 | 407 | 1.02 |
| 1BS0 | 2936 | 71554 | 487 | 21 | 8(4) | 8(5) | 4(1) | 1(0) | 0(0) | 0(0) | 0(0) | 0(0) | 0(0) | 0(0) | 134 | 82 | 1183 | 1.02 |
| 1BYO | 731 | 18213 | 37 | 3 | 3(0) | 0(0) | 0(0) | 0(0) | 0(0) | 0(0) | 0(0) | 0(0) | 0(0) | 0(0) | 9 | 7 | 320 | 1.01 |
| 1CAQ | 1339 | 33096 | 267 | 10 | 7(3) | 0(0) | 0(0) | 1(0) | 1(0) | 1(1) | 0(0) | 0(0) | 0(0) | 0(0) | 56 | 33 | 553 | 1.02 |
| 1CK1 | 1944 | 48217 | 475 | 17 | 8(2) | 5(2) | 1(1) | 1(0) | 0(0) | 1(1) | 0(0) | 1(1) | 0(0) | 0(0) | 117 | 70 | 828 | 1.05 |
| 1CQX | 3158 | 77872 | 663 | 26 | 15(4) | 4(2) | 2(1) | 1(1) | 3(1) | 1(0) | 0(0) | 0(0) | 0(0) | 0(0) | 162 | 88 | 1216 | 0.97 |
| 1DYS | 2641 | 60688 | 545 | 19 | 11(7) | 4(2) | 1(1) | 1(1) | 0(0) | 1(1) | 0(0) | 0(0) | 0(0) | 1(1) | 149 | 83 | 1210 | 0.98 |
| 1E02 | 1186 | 29357 | 621 | 6 | 3(1) | 1(1) | 0(0) | 1(1) | 0(0) | 0(0) | 0(0) | 0(0) | 0(0) | 1(0) | 88 | 36 | 426 | 1.01 |
| 1EHY | 2317 | 53646 | 821 | 18 | 9(4) | 2(0) | 2(1) | 2(0) | 0(0) | 0(0) | 1(1) | 0(0) | 0(0) | 2(2) | 163 | 78 | 1057 | 1.01 |
| 1EPA | 1202 | 27830 | 150 | 7 | 3(0) | 3(0) | 1(0) | 0(0) | 0(0) | 0(0) | 0(0) | 0(0) | 0(0) | 0(0) | 45 | 28 | 429 | 0.99 |
| 1EW0 | 1019 | 26176 | 0 | 0 | 0(0) | 0(0) | 0(0) | 0(0) | 0(0) | 0(0) | 0(0) | 0(0) | 0(0) | 0(0) | 0 | 0 | 329 | 0.99 |
| 1FEH | 4461 | 106104 | 2393 | 52 | 29(15) | 9(4) | 1(0) | 1(1) | 0(0) | 2(1) | 4(3) | 1(0) | 1(1) | 4(1) | 436 | 222 | 1972 | 1 |
| 1FGK | 2190 | 54411 | 337 | 9 | 5(2) | 1(0) | 2(1) | 0(0) | 0(0) | 0(0) | 0(0) | 0(0) | 0(0) | 1(1) | 83 | 43 | 842 | 0.96 |
| 1FJM | 2303 | 53814 | 422 | 14 | 7(4) | 1(0) | 2(0) | 2(0) | 1(1) | 1(1) | 0(0) | 0(0) | 0(0) | 0(0) | 108 | 64 | 1122 | 0.99 |
| 1FKD | 832 | 21388 | 92 | 3 | 1(1) | 1(0) | 0(0) | 0(0) | 1(1) | 0(0) | 0(0) | 0(0) | 0(0) | 0(0) | 29 | 18 | 315 | 1.06 |
| 1FM2 | 1185 | 30238 | 160 | 5 | 3(2) | 0(0) | 0(0) | 1(1) | 0(0) | 0(0) | 1(0) | 0(0) | 0(0) | 0(0) | 43 | 24 | 406 | 0.99 |
| 1G2A | 1315 | 33144 | 141 | 5 | 4(1) | 0(0) | 0(0) | 0(0) | 0(0) | 0(0) | 1(1) | 0(0) | 0(0) | 0(0) | 39 | 20 | 515 | 0.98 |
| 1GAR | 1554 | 39168 | 211 | 8 | 4(1) | 1(1) | 1(0) | 2(1) | 0(0) | 0(0) | 0(0) | 0(0) | 0(0) | 0(0) | 53 | 30 | 623 | 1.03 |
| 1HF0 | 1038 | 27902 | 61 | 3 | 1(0) | 2(0) | 0(0) | 0(0) | 0(0) | 0(0) | 0(0) | 0(0) | 0(0) | 0(0) | 19 | 11 | 322 | 0.95 |
| 1HF8 | 2114 | 52975 | 934 | 23 | 7(1) | 6(1) | 3(1) | 0(0) | 1(0) | 1(0) | 1(0) | 1(0) | 3(2) | 0(0) | 199 | 87 | 835 | 1.01 |
| 1ILR | 1144 | 29060 | 322 | 12 | 6(5) | 3(1) | 1(1) | 0(0) | 1(1) | 1(0) | 0(0) | 0(0) | 0(0) | 0(0) | 79 | 43 | 431 | 1.02 |
| 1KPT | 798 | 18786 | 68 | 4 | 3(2) | 1(1) | 0(0) | 0(0) | 0(0) | 0(0) | 0(0) | 0(0) | 0(0) | 0(0) | 19 | 13 | 303 | 1.02 |
| 1KWA | 696 | 18379 | 108 | 6 | 4(0) | 2(0) | 0(0) | 0(0) | 0(0) | 0(0) | 0(0) | 0(0) | 0(0) | 0(0) | 28 | 21 | 223 | 1.03 |
| 1MP2 | 1467 | 37312 | 78 | 5 | 4(2) | 1(1) | 0(0) | 0(0) | 0(0) | 0(0) | 0(0) | 0(0) | 0(0) | 0(0) | 29 | 21 | 559 | 0.99 |
| 1NA1 | 2170 | 57892 | 248 | 10 | 3(2) | 3(1) | 4(1) | 0(0) | 0(0) | 0(0) | 0(0) | 0(0) | 0(0) | 0(0) | 71 | 43 | 635 | 1 |
| 1NP4 | 1428 | 36010 | 135 | 7 | 5(3) | 1(1) | 0(0) | 1(0) | 0(0) | 0(0) | 0(0) | 0(0) | 0(0) | 0(0) | 43 | 30 | 538 | 1.04 |
| 1PB1 | 3213 | 77715 | 598 | 23 | 14(10) | 2(0) | 3(3) | 1(0) | 2(1) | 1(1) | 0(0) | 0(0) | 0(0) | 0(0) | 162 | 95 | 1394 | 1 |
| 1PP1 | 2531 | 65974 | 234 | 11 | 8(1) | 1(0) | 1(1) | 0(0) | 1(1) | 0(0) | 0(0) | 0(0) | 0(0) | 0(0) | 59 | 39 | 861 | 1.03 |
| 1PP2 | 946 | 24226 | 125 | 7 | 6(2) | 0(0) | 1(1) | 0(0) | 0(0) | 0(0) | 0(0) | 0(0) | 0(0) | 0(0) | 35 | 24 | 330 | 1 |
| 1PP3 | 1551 | 37846 | 130 | 6 | 4(3) | 0(0) | 2(2) | 0(0) | 0(0) | 0(0) | 0(0) | 0(0) | 0(0) | 0(0) | 42 | 27 | 652 | 1.02 |
| 1QAZ | 2793 | 66239 | 582 | 22 | 12(8) | 4(2) | 4(2) | 0(0) | 0(0) | 1(1) | 0(0) | 0(0) | 1(1) | 0(0) | 148 | 89 | 1297 | 1.04 |
| 1QCI | 2060 | 48846 | 376 | 15 | 7(2) | 5(1) | 1(0) | 0(0) | 1(0) | 1(0) | 0(0) | 0(0) | 0(0) | 0(0) | 92 | 60 | 978 | 1.01 |
| 1QJP | 1101 | 27706 | 513 | 12 | 4(2) | 4(3) | 0(0) | 0(0) | 0(0) | 3(3) | 0(0) | 0(0) | 0(0) | 1(1) | 114 | 49 | 284 | 1.01 |
| 1QPA | 2555 | 61074 | 915 | 22 | 9(6) | 3(3) | 1(1) | 2(1) | 1(1) | 2(2) | 0(0) | 1(1) | 1(1) | 2(2) | 192 | 93 | 1064 | 1 |
| 1QT1 | 3009 | 73978 | 984 | 22 | 12(5) | 4(2) | 1(1) | 2(2) | 1(1) | 0(0) | 0(0) | 0(0) | 0(0) | 2(2) | 191 | 102 | 1155 | 1.02 |
| 1RB3 | 1268 | 31415 | 60 | 4 | 4(1) | 0(0) | 0(0) | 0(0) | 0(0) | 0(0) | 0(0) | 0(0) | 0(0) | 0(0) | 21 | 16 | 499 | 0.96 |
| 1RNE | 2510 | 62185 | 649 | 20 | 11(4) | 2(1) | 3(1) | 1(0) | 1(1) | 0(0) | 0(0) | 0(0) | 0(0) | 2(1) | 133 | 73 | 1160 | 1 |
| 1SHK | 1240 | 30043 | 155 | 6 | 3(1) | 2(1) | 0(0) | 0(0) | 1(1) | 0(0) | 0(0) | 0(0) | 0(0) | 0(0) | 41 | 28 | 478 | 0.98 |
| 1THE | 1928 | 45846 | 401 | 16 | 9(5) | 4(1) | 1(1) | 0(0) | 0(0) | 1(1) | 1(1) | 0(0) | 0(0) | 0(0) | 105 | 59 | 849 | 0.99 |
| 1THZ | 4511 | 111337 | 993 | 35 | 21(8) | 5(2) | 3(1) | 0(0) | 2(1) | 1(1) | 1(1) | 0(0) | 1(1) | 1(1) | 252 | 148 | 1804 | 1.02 |
| 1TOA | 2147 | 51293 | 355 | 12 | 7(4) | 2(0) | 1(0) | 0(0) | 1(1) | 0(0) | 0(0) | 0(0) | 0(0) | 1(0) | 95 | 54 | 945 | 1.01 |
| 1TON | 1734 | 41135 | 569 | 16 | 8(6) | 3(2) | 2(1) | 0(0) | 0(0) | 0(0) | 2(2) | 0(0) | 0(0) | 1(1) | 112 | 63 | 771 | 0.99 |
| 1VBT | 1258 | 30154 | 324 | 12 | 4(2) | 3(2) | 2(1) | 2(1) | 1(1) | 0(0) | 0(0) | 0(0) | 0(0) | 0(0) | 88 | 52 | 581 | 1.01 |
| 1XGS | 2312 | 55622 | 157 | 7 | 3(2) | 3(3) | 1(1) | 0(0) | 0(0) | 0(0) | 0(0) | 0(0) | 0(0) | 0(0) | 49 | 36 | 1111 | 0.99 |
| 256B | 826 | 21324 | 25 | 2 | 2(1) | 0(0) | 0(0) | 0(0) | 0(0) | 0(0) | 0(0) | 0(0) | 0(0) | 0(0) | 10 | 9 | 262 | 1 |
| 256L | 1309 | 32380 | 308 | 11 | 6(3) | 2(1) | 1(0) | 0(0) | 1(0) | 0(0) | 1(1) | 0(0) | 0(0) | 0(0) | 72 | 38 | 527 | 1.01 |
| 2ACY | 784 | 19824 | 24 | 2 | 2(2) | 0(0) | 0(0) | 0(0) | 0(0) | 0(0) | 0(0) | 0(0) | 0(0) | 0(0) | 9 | 7 | 321 | 0.99 |
| 2ATJ | 2386 | 56708 | 548 | 18 | 10(6) | 2(0) | 2(2) | 1(1) | 0(0) | 0(0) | 2(2) | 0(0) | 0(0) | 1(1) | 130 | 73 | 1060 | 1.01 |
| 2BC2 | 1681 | 40775 | 338 | 13 | 5(2) | 4(3) | 1(0) | 2(2) | 0(0) | 1(0) | 0(0) | 0(0) | 0(0) | 0(0) | 87 | 52 | 757 | 0.99 |
| 2G3P | 1580 | 39017 | 246 | 9 | 5(3) | 2(2) | 1(1) | 0(0) | 0(0) | 0(0) | 0(0) | 0(0) | 1(1) | 0(0) | 60 | 38 | 612 | 0.96 |
| 2IHL | 1016 | 24683 | 176 | 6 | 3(2) | 2(1) | 0(0) | 0(0) | 0(0) | 0(0) | 1(1) | 0(0) | 0(0) | 0(0) | 44 | 25 | 415 | 0.99 |
| 2MBR | 2654 | 63365 | 317 | 16 | 11(7) | 4(3) | 0(0) | 1(1) | 0(0) | 0(0) | 0(0) | 0(0) | 0(0) | 0(0) | 87 | 63 | 1118 | 1 |
| 2SCP | 1368 | 33009 | 450 | 20 | 12(4) | 4(3) | 2(1) | 1(1) | 0(0) | 1(1) | 0(0) | 0(0) | 0(0) | 0(0) | 108 | 58 | 558 | 1 |
| 2SHP | 3959 | 95129 | 1207 | 37 | 19(13) | 6(2) | 3(3) | 3(2) | 3(3) | 0(0) | 2(2) | 0(0) | 0(0) | 1(1) | 273 | 143 | 1727 | 1.01 |
| 2TPS | 1713 | 41799 | 153 | 8 | 5(3) | 3(2) | 0(0) | 0(0) | 0(0) | 0(0) | 0(0) | 0(0) | 0(0) | 0(0) | 43 | 29 | 709 | 1.02 |
| 2UGI | 654 | 17367 | 111 | 7 | 6(2) | 1(0) | 0(0) | 0(0) | 0(0) | 0(0) | 0(0) | 0(0) | 0(0) | 0(0) | 25 | 17 | 223 | 0.98 |
| 3PMG | 4329 | 101915 | 1375 | 40 | 17(9) | 9(5) | 5(2) | 3(1) | 1(1) | 0(0) | 0(0) | 1(1) | 1(1) | 3(3) | 323 | 186 | 2008 | 1.03 |
| 830C | 1307 | 32412 | 42 | 3 | 3(2) | 0(0) | 0(0) | 0(0) | 0(0) | 0(0) | 0(0) | 0(0) | 0(0) | 0(0) | 17 | 12 | 536 | 0.99 |
| 8PTI | 446 | 11686 | 0 | 0 | 0(0) | 0(0) | 0(0) | 0(0) | 0(0) | 0(0) | 0(0) | 0(0) | 0(0) | 0(0) | 0 | 0 | 159 | 0.96 |
| 1AD5 | 3470 | 86575 | 835 | 34 | 20 | 4 | 4 | 3 | 1 | 2 | 0 | 0 | 0 | 0 | 215 | 119 | 1405 | 1.03 |
| 1AFK | 951 | 23738 | 15 | 1 | 1 | 0 | 0 | 0 | 0 | 0 | 0 | 0 | 0 | 0 | 5 | 4 | 377 | 0.99 |
| 1B3J | 2116 | 53259 | 313 | 11 | 7 | 1 | 1 | 0 | 1 | 0 | 0 | 0 | 1 | 0 | 79 | 48 | 749 | 1.02 |
| 1C02 | 1339 | 33535 | 466 | 11 | 2 | 5 | 0 | 1 | 0 | 1 | 0 | 1 | 0 | 1 | 104 | 52 | 494 | 1.01 |
| 1CK2 | 795 | 20334 | 141 | 5 | 2 | 1 | 1 | 1 | 0 | 0 | 0 | 0 | 0 | 0 | 47 | 31 | 272 | 1.02 |
| 1CKI | 2388 | 58345 | 687 | 20 | 8 | 6 | 3 | 1 | 0 | 1 | 0 | 0 | 0 | 1 | 141 | 69 | 994 | 1.03 |
| 1CLU | 1262 | 31228 | 274 | 10 | 3 | 5 | 1 | 0 | 0 | 1 | 0 | 0 | 0 | 0 | 72 | 45 | 517 | 1.01 |
| 1DSU | 1712 | 40597 | 571 | 18 | 10 | 4 | 1 | 1 | 0 | 0 | 1 | 0 | 0 | 1 | 119 | 67 | 758 | 0.98 |
| 1FM1 | 1254 | 32443 | 422 | 17 | 12 | 2 | 2 | 0 | 0 | 0 | 0 | 0 | 0 | 1 | 97 | 52 | 414 | 1.05 |
| 1MP1 | 901 | 22921 | 46 | 3 | 2 | 1 | 0 | 0 | 0 | 0 | 0 | 0 | 0 | 0 | 15 | 10 | 340 | 0.94 |
| 1MSS | 1821 | 44383 | 522 | 19 | 10 | 6 | 0 | 0 | 1 | 0 | 0 | 2 | 0 | 0 | 124 | 72 | 775 | 1.04 |
| 1PD0 | 5816 | 137941 | 1753 | 49 | 20 | 12 | 4 | 2 | 1 | 2 | 3 | 3 | 1 | 1 | 423 | 220 | 2621 | 1 |
| 1QDM | 3127 | 74165 | 870 | 27 | 13 | 5 | 1 | 2 | 3 | 1 | 0 | 1 | 0 | 1 | 196 | 113 | 1487 | 1 |
| 1QHA | 7065 | 169367 | 1856 | 66 | 39 | 12 | 7 | 4 | 0 | 1 | 0 | 0 | 0 | 3 | 416 | 231 | 3125 | 1 |
| 1QM0 | 877 | 23035 | 363 | 10 | 3 | 3 | 0 | 1 | 1 | 1 | 0 | 1 | 0 | 0 | 70 | 35 | 236 | 1 |
| 1RH0 | 3394 | 79586 | 776 | 26 | 12 | 7 | 2 | 1 | 1 | 0 | 0 | 3 | 0 | 0 | 193 | 104 | 1667 | 0.99 |
| 1THT | 2295 | 55642 | 985 | 26 | 13 | 6 | 2 | 0 | 0 | 1 | 2 | 1 | 0 | 1 | 207 | 103 | 945 | 1.02 |
| 1URP | 2002 | 48789 | 103 | 4 | 3 | 0 | 0 | 0 | 1 | 0 | 0 | 0 | 0 | 0 | 26 | 19 | 927 | 1.01 |
| 1URZ | 2921 | 73341 | 665 | 24 | 15 | 3 | 4 | 0 | 0 | 0 | 0 | 0 | 1 | 1 | 141 | 85 | 1086 | 0.97 |
| 2BLS | 2790 | 64534 | 731 | 24 | 13 | 4 | 2 | 2 | 2 | 0 | 0 | 0 | 0 | 1 | 190 | 103 | 1304 | 1 |
| 2ERC | 1967 | 48006 | 286 | 12 | 7 | 3 | 1 | 0 | 0 | 0 | 1 | 0 | 0 | 0 | 75 | 56 | 786 | 0.98 |
| 3MHT | 2606 | 63371 | 903 | 37 | 22 | 7 | 2 | 1 | 4 | 0 | 0 | 1 | 0 | 0 | 214 | 120 | 1139 | 1.04 |
| 5TSS | 1559 | 38160 | 332 | 14 | 9 | 1 | 2 | 0 | 1 | 1 | 0 | 0 | 0 | 0 | 81 | 47 | 621 | 1 |
| 12as | 2559 | 61430 | 589 | 23 | 12(5) | 4(0) | 3(0) | 0(0) | 3(1) | 0(0) | 1(1) | 0(0) | 0(0) | 0(0) | 136 | 76 | 1109 | 0.99 |
| 1a3c | 1378 | 32879 | 148 | 5 | 2(1) | 1(0) | 1(1) | 0(0) | 0(0) | 1(1) | 0(0) | 0(0) | 0(0) | 0(0) | 45 | 25 | 505 | 0.96 |
| 1a4i | 2159 | 53225 | 353 | 11 | 6(1) | 1(1) | 2(2) | 0(0) | 0(0) | 0(0) | 1(1) | 1(1) | 0(0) | 0(0) | 93 | 59 | 913 | 0.99 |
| 1a4u | 1963 | 48623 | 534 | 17 | 10(4) | 1(1) | 3(2) | 0(0) | 2(1) | 0(0) | 0(0) | 0(0) | 0(0) | 1(1) | 120 | 68 | 837 | 1.05 |
| 1aa7 | 1218 | 30087 | 232 | 9 | 6(1) | 1(0) | 1(1) | 0(0) | 0(0) | 0(0) | 0(0) | 0(0) | 1(1) | 0(0) | 54 | 36 | 531 | 1.01 |
| 1ad3 | 3477 | 84369 | 1044 | 33 | 14(7) | 11(2) | 3(2) | 0(0) | 1(1) | 1(1) | 1(1) | 0(0) | 0(0) | 2(2) | 235 | 134 | 1525 | 1.01 |
| 1ade | 3379 | 80948 | 1459 | 40 | 16(7) | 9(5) | 6(1) | 1(1) | 4(2) | 0(0) | 1(1) | 1(1) | 0(0) | 2(1) | 306 | 157 | 1350 | 1.03 |
| 1afw | 2923 | 68436 | 702 | 29 | 17(10) | 6(2) | 3(0) | 0(0) | 1(1) | 2(2) | 0(0) | 0(0) | 0(0) | 0(0) | 168 | 94 | 1450 | 1.02 |
| 1ajs | 3274 | 78627 | 833 | 27 | 13(5) | 5(4) | 3(1) | 2(1) | 1(0) | 1(1) | 0(0) | 1(1) | 0(0) | 1(1) | 181 | 109 | 1464 | 1.01 |
| 1alo | 6812 | 155051 | 3142 | 57 | 22(13) | 7(6) | 5(4) | 4(4) | 6(6) | 0(0) | 3(2) | 1(1) | 1(1) | 8(8) | 652 | 303 | 3346 | 1.01 |
| 1amk | 1906 | 46243 | 347 | 14 | 5(3) | 5(1) | 2(1) | 2(1) | 0(0) | 0(0) | 0(0) | 0(0) | 0(0) | 0(0) | 95 | 57 | 832 | 1.01 |
| 1aor | 4693 | 106572 | 1407 | 46 | 15(5) | 16(8) | 4(2) | 3(1) | 6(4) | 1(0) | 0(0) | 0(0) | 0(0) | 1(1) | 319 | 181 | 2496 | 1.02 |
| 1aq6 | 1886 | 46135 | 387 | 11 | 5(3) | 2(1) | 1(1) | 2(2) | 0(0) | 0(0) | 0(0) | 0(0) | 0(0) | 1(1) | 93 | 58 | 837 | 1.02 |
| 1auo | 1682 | 40418 | 299 | 10 | 6(2) | 1(1) | 1(0) | 0(0) | 0(0) | 1(1) | 0(0) | 1(1) | 0(0) | 0(0) | 76 | 41 | 778 | 1.04 |
| 1b3a | 545 | 14624 | 20 | 1 | 0(0) | 1(1) | 0(0) | 0(0) | 0(0) | 0(0) | 0(0) | 0(0) | 0(0) | 0(0) | 7 | 6 | 160 | 0.98 |
| 1b5e | 1973 | 48329 | 359 | 17 | 9(3) | 4(2) | 3(3) | 1(1) | 0(0) | 0(0) | 0(0) | 0(0) | 0(0) | 0(0) | 105 | 67 | 836 | 1.02 |
| 1b67 | 514 | 14352 | 16 | 1 | 1(0) | 0(0) | 0(0) | 0(0) | 0(0) | 0(0) | 0(0) | 0(0) | 0(0) | 0(0) | 5 | 3 | 118 | 0.95 |
| 1b8a | 3594 | 87466 | 594 | 21 | 13(7) | 2(0) | 0(0) | 2(2) | 3(3) | 0(0) | 0(0) | 0(0) | 1(1) | 0(0) | 136 | 78 | 1479 | 0.98 |
| 1b8j | 3298 | 78352 | 960 | 27 | 10(9) | 6(1) | 3(3) | 2(2) | 1(1) | 0(0) | 2(2) | 2(2) | 0(0) | 1(1) | 234 | 134 | 1508 | 1.01 |
| 1bam | 1619 | 39372 | 131 | 7 | 3(0) | 4(2) | 0(0) | 0(0) | 0(0) | 0(0) | 0(0) | 0(0) | 0(0) | 0(0) | 47 | 31 | 755 | 1.01 |
| 1bbh | 967 | 24880 | 40 | 3 | 3(1) | 0(0) | 0(0) | 0(0) | 0(0) | 0(0) | 0(0) | 0(0) | 0(0) | 0(0) | 14 | 10 | 320 | 0.99 |
| 1bd0 | 3026 | 72287 | 548 | 22 | 11(6) | 6(3) | 2(1) | 0(0) | 3(3) | 0(0) | 0(0) | 0(0) | 0(0) | 0(0) | 155 | 92 | 1342 | 1.01 |
| 1bif | 3533 | 83520 | 367 | 18 | 11(7) | 6(1) | 0(0) | 1(1) | 0(0) | 0(0) | 0(0) | 0(0) | 0(0) | 0(0) | 101 | 63 | 1487 | 0.98 |
| 1biq | 2773 | 65811 | 1378 | 29 | 13(4) | 6(3) | 2(1) | 1(1) | 1(1) | 1(0) | 0(0) | 1(1) | 1(1) | 3(1) | 260 | 128 | 1219 | 1.02 |
| 1bis | 1121 | 28888 | 200 | 8 | 3(1) | 3(1) | 1(0) | 1(0) | 0(0) | 0(0) | 0(0) | 0(0) | 0(0) | 0(0) | 57 | 33 | 396 | 1.06 |
| 1bjw | 2936 | 71781 | 1057 | 27 | 9(5) | 6(5) | 4(2) | 1(1) | 1(0) | 2(2) | 0(0) | 2(2) | 0(0) | 2(2) | 228 | 126 | 1243 | 1.05 |
| 1bkp | 2307 | 55987 | 418 | 16 | 10(8) | 3(1) | 1(0) | 1(1) | 0(0) | 0(0) | 0(0) | 1(1) | 0(0) | 0(0) | 116 | 69 | 1006 | 1.01 |
| 1bmd | 2493 | 60191 | 618 | 23 | 13(4) | 4(4) | 1(1) | 2(2) | 1(1) | 1(1) | 1(1) | 0(0) | 0(0) | 0(0) | 157 | 93 | 1093 | 1 |
| 1brw | 3217 | 78872 | 1420 | 32 | 14(1) | 5(1) | 2(2) | 3(0) | 1(1) | 2(1) | 2(1) | 1(0) | 0(0) | 2(1) | 271 | 144 | 1371 | 1.01 |
| 1bsl | 2559 | 61151 | 449 | 14 | 5(2) | 2(0) | 3(3) | 0(0) | 2(2) | 2(1) | 0(0) | 0(0) | 0(0) | 0(0) | 113 | 66 | 1058 | 1 |
| 1bsr | 946 | 25584 | 40 | 2 | 1(1) | 1(1) | 0(0) | 0(0) | 0(0) | 0(0) | 0(0) | 0(0) | 0(0) | 0(0) | 14 | 7 | 293 | 0.98 |
| 1buo | 972 | 25847 | 142 | 5 | 3(0) | 1(0) | 0(0) | 0(0) | 0(0) | 1(0) | 0(0) | 0(0) | 0(0) | 0(0) | 33 | 22 | 322 | 0.94 |
| 1bxg | 2517 | 60193 | 512 | 15 | 7(2) | 3(0) | 1(1) | 0(0) | 0(0) | 1(0) | 1(0) | 1(1) | 1(1) | 0(0) | 123 | 76 | 1098 | 1.01 |
| 1bxk | 2700 | 64257 | 322 | 9 | 2(1) | 4(3) | 1(1) | 0(0) | 0(0) | 1(1) | 0(0) | 0(0) | 1(1) | 0(0) | 79 | 48 | 1159 | 1.01 |
| 1cdc | 759 | 22821 | 43 | 2 | 1(0) | 1(1) | 0(0) | 0(0) | 0(0) | 0(0) | 0(0) | 0(0) | 0(0) | 0(0) | 16 | 9 | 134 | 0.97 |
| 1cg2 | 2790 | 68324 | 617 | 21 | 13(5) | 1(0) | 4(2) | 0(0) | 0(0) | 2(1) | 0(0) | 0(0) | 0(0) | 1(1) | 157 | 102 | 1188 | 1.02 |
| 1chm | 3187 | 75180 | 1078 | 27 | 8(7) | 8(5) | 3(2) | 2(2) | 4(4) | 0(0) | 1(1) | 0(0) | 0(0) | 1(1) | 238 | 131 | 1459 | 1.01 |
| 1cmb | 845 | 22815 | 368 | 3 | 2(2) | 0(0) | 0(0) | 0(0) | 0(0) | 0(0) | 0(0) | 0(0) | 0(0) | 1(1) | 54 | 24 | 246 | 0.98 |
| 1cnz | 2776 | 66255 | 686 | 24 | 17(7) | 4(1) | 0(0) | 1(0) | 0(0) | 0(0) | 0(0) | 1(1) | 0(0) | 1(1) | 158 | 90 | 1212 | 1 |
| 1coz | 1061 | 26818 | 137 | 3 | 2(1) | 0(0) | 0(0) | 0(0) | 0(0) | 0(0) | 0(0) | 0(0) | 0(0) | 1(1) | 29 | 19 | 422 | 1.03 |
| 1ctt | 2220 | 52343 | 582 | 19 | 7(1) | 6(4) | 1(1) | 2(1) | 1(0) | 1(1) | 1(1) | 0(0) | 0(0) | 0(0) | 143 | 88 | 1016 | 1.03 |
| 1cvu | 4469 | 104857 | 1124 | 35 | 15(7) | 9(2) | 5(3) | 2(2) | 1(1) | 0(0) | 0(0) | 0(0) | 2(2) | 1(1) | 274 | 155 | 1950 | 0.97 |
| 1czj | 862 | 23831 | 30 | 2 | 2(1) | 0(0) | 0(0) | 0(0) | 0(0) | 0(0) | 0(0) | 0(0) | 0(0) | 0(0) | 11 | 7 | 190 | 0.93 |
| 1daa | 2233 | 55482 | 569 | 16 | 5(2) | 6(3) | 1(0) | 1(1) | 0(0) | 2(2) | 0(0) | 0(0) | 0(0) | 1(1) | 130 | 75 | 899 | 1 |
| 1dor | 2409 | 58341 | 296 | 16 | 12(6) | 3(2) | 1(1) | 0(0) | 0(0) | 0(0) | 0(0) | 0(0) | 0(0) | 0(0) | 93 | 65 | 1104 | 1.04 |
| 1dpg | 3840 | 92376 | 1345 | 52 | 27(10) | 11(6) | 7(2) | 5(1) | 1(0) | 0(0) | 0(0) | 0(0) | 0(0) | 1(0) | 311 | 163 | 1619 | 1.03 |
| 1dqs | 2843 | 68681 | 735 | 27 | 16(1) | 4(0) | 2(1) | 2(2) | 0(0) | 0(0) | 2(1) | 1(0) | 0(0) | 0(0) | 178 | 105 | 1250 | 1.02 |
| 1dxg | 261 | 7423 | 56 | 2 | 1(1) | 0(0) | 0(0) | 1(1) | 0(0) | 0(0) | 0(0) | 0(0) | 0(0) | 0(0) | 12 | 7 | 66 | 0.94 |
| 1e98 | 1665 | 40638 | 111 | 7 | 5(3) | 2(1) | 0(0) | 0(0) | 0(0) | 0(0) | 0(0) | 0(0) | 0(0) | 0(0) | 37 | 27 | 688 | 1.03 |
| 1ebh | 3317 | 77183 | 1367 | 31 | 11(3) | 7(4) | 2(0) | 2(1) | 5(5) | 0(0) | 2(2) | 0(0) | 0(0) | 2(2) | 307 | 165 | 1554 | 1.02 |
| 1f13 | 5791 | 134751 | 2139 | 78 | 39(17) | 21(9) | 7(2) | 3(2) | 4(3) | 0(0) | 0(0) | 2(1) | 0(0) | 2(1) | 502 | 277 | 2560 | 1.02 |
| 1fip | 585 | 16415 | 93 | 4 | 2(1) | 1(0) | 1(0) | 0(0) | 0(0) | 0(0) | 0(0) | 0(0) | 0(0) | 0(0) | 24 | 14 | 140 | 0.96 |
| 1fro | 1400 | 36876 | 155 | 8 | 4(1) | 3(1) | 1(0) | 0(0) | 0(0) | 0(0) | 0(0) | 0(0) | 0(0) | 0(0) | 37 | 19 | 467 | 0.98 |
| 1gvp | 682 | 18948 | 83 | 2 | 1(1) | 0(0) | 0(0) | 0(0) | 0(0) | 1(1) | 0(0) | 0(0) | 0(0) | 0(0) | 18 | 10 | 186 | 0.99 |
| 1hjr | 1192 | 31229 | 144 | 8 | 6(0) | 1(0) | 1(0) | 0(0) | 0(0) | 0(0) | 0(0) | 0(0) | 0(0) | 0(0) | 36 | 23 | 442 | 1.01 |
| 1hss | 840 | 21380 | 42 | 1 | 0(0) | 0(0) | 0(0) | 1(0) | 0(0) | 0(0) | 0(0) | 0(0) | 0(0) | 0(0) | 11 | 7 | 321 | 1.04 |
| 1hxp | 2713 | 66476 | 804 | 23 | 11(3) | 7(4) | 2(1) | 0(0) | 0(0) | 0(0) | 1(1) | 1(1) | 0(0) | 1(1) | 197 | 100 | 1103 | 0.99 |
| 1icw | 567 | 15495 | 132 | 4 | 1(0) | 1(1) | 1(0) | 0(0) | 1(1) | 0(0) | 0(0) | 0(0) | 0(0) | 0(0) | 34 | 20 | 138 | 0.98 |
| 1imb | 2073 | 50794 | 317 | 16 | 11(4) | 3(1) | 1(0) | 0(0) | 0(0) | 1(1) | 0(0) | 0(0) | 0(0) | 0(0) | 94 | 61 | 909 | 1.01 |
| 1isa | 1503 | 36320 | 316 | 12 | 4(1) | 2(2) | 5(3) | 1(0) | 0(0) | 0(0) | 0(0) | 0(0) | 0(0) | 0(0) | 77 | 42 | 663 | 1.03 |
| 1ivy | 3600 | 83773 | 884 | 32 | 17(1) | 7(1) | 4(1) | 0(0) | 1(0) | 0(0) | 1(1) | 1(0) | 1(1) | 0(0) | 222 | 111 | 1659 | 0.99 |
| 1jhg | 876 | 22593 | 27 | 2 | 2(0) | 0(0) | 0(0) | 0(0) | 0(0) | 0(0) | 0(0) | 0(0) | 0(0) | 0(0) | 10 | 8 | 205 | 0.91 |
| 1jsg | 924 | 23954 | 20 | 1 | 0(0) | 1(0) | 0(0) | 0(0) | 0(0) | 0(0) | 0(0) | 0(0) | 0(0) | 0(0) | 6 | 5 | 355 | 0.99 |
| 1kba | 501 | 13020 | 0 | 0 | 0(0) | 0(0) | 0(0) | 0(0) | 0(0) | 0(0) | 0(0) | 0(0) | 0(0) | 0(0) | 0 | 0 | 184 | 0.95 |
| 1kpf | 866 | 22625 | 228 | 4 | 2(1) | 0(0) | 0(0) | 0(0) | 0(0) | 0(0) | 1(0) | 0(0) | 0(0) | 1(1) | 43 | 18 | 293 | 0.98 |
| 1m6p | 1162 | 29393 | 202 | 8 | 2(2) | 3(1) | 2(0) | 1(1) | 0(0) | 0(0) | 0(0) | 0(0) | 0(0) | 0(0) | 58 | 38 | 442 | 0.97 |
| 1mkb | 1325 | 33184 | 202 | 6 | 2(1) | 0(0) | 2(0) | 1(1) | 1(1) | 0(0) | 0(0) | 0(0) | 0(0) | 0(0) | 55 | 37 | 523 | 1.03 |
| 1mor | 2825 | 67087 | 1214 | 25 | 7(1) | 10(5) | 3(3) | 3(2) | 1(0) | 0(0) | 0(0) | 0(0) | 0(0) | 1(1) | 232 | 119 | 1310 | 0.99 |
| 1nox | 1571 | 41533 | 352 | 12 | 8(2) | 1(0) | 0(0) | 1(0) | 0(0) | 0(0) | 1(0) | 1(1) | 0(0) | 0(0) | 69 | 44 | 562 | 1.05 |
| 1nse | 3302 | 79968 | 542 | 21 | 11(9) | 4(3) | 3(1) | 1(1) | 1(0) | 0(0) | 0(0) | 1(1) | 0(0) | 0(0) | 144 | 89 | 1369 | 1.01 |
| 1nsy | 2132 | 53386 | 364 | 11 | 4(1) | 4(3) | 1(1) | 0(0) | 0(0) | 0(0) | 1(1) | 0(0) | 0(0) | 1(1) | 90 | 55 | 788 | 1.02 |
| 1oac | 5665 | 138608 | 2642 | 52 | 23(12) | 6(2) | 8(7) | 2(2) | 3(3) | 0(0) | 2(2) | 3(3) | 1(1) | 4(4) | 495 | 218 | 2292 | 1.01 |
| 1opy | 957 | 24631 | 99 | 5 | 3(3) | 2(1) | 0(0) | 0(0) | 0(0) | 0(0) | 0(0) | 0(0) | 0(0) | 0(0) | 35 | 25 | 345 | 0.97 |
| 1pgt | 1645 | 40645 | 398 | 15 | 10(6) | 2(1) | 2(0) | 0(0) | 0(0) | 0(0) | 0(0) | 0(0) | 0(0) | 1(1) | 100 | 61 | 674 | 1.03 |
| 1qfh | 1551 | 40394 | 189 | 8 | 4(1) | 3(1) | 0(0) | 1(1) | 0(0) | 0(0) | 0(0) | 0(0) | 0(0) | 0(0) | 54 | 31 | 546 | 0.99 |
| 1qhi | 2304 | 54725 | 1111 | 22 | 10(4) | 2(1) | 1(0) | 1(1) | 2(1) | 1(1) | 1(1) | 1(0) | 0(0) | 3(3) | 210 | 105 | 977 | 1 |
| 1qr2 | 1824 | 44293 | 285 | 12 | 4(1) | 5(4) | 3(1) | 0(0) | 0(0) | 0(0) | 0(0) | 0(0) | 0(0) | 0(0) | 69 | 45 | 763 | 1 |
| 1r2f | 2269 | 53893 | 621 | 20 | 10(5) | 6(2) | 0(0) | 0(0) | 1(1) | 1(0) | 1(1) | 0(0) | 0(0) | 1(1) | 143 | 75 | 1046 | 1 |
| 1reg | 1026 | 26280 | 63 | 3 | 2(1) | 0(0) | 1(0) | 0(0) | 0(0) | 0(0) | 0(0) | 0(0) | 0(0) | 0(0) | 19 | 13 | 384 | 1 |
| 1rpo | 531 | 13492 | 42 | 1 | 0(0) | 0(0) | 0(0) | 1(1) | 0(0) | 0(0) | 0(0) | 0(0) | 0(0) | 0(0) | 12 | 6 | 138 | 0.96 |
| 1ses | 3373 | 80391 | 109 | 4 | 2(2) | 1(1) | 0(0) | 0(0) | 1(1) | 0(0) | 0(0) | 0(0) | 0(0) | 0(0) | 33 | 20 | 1502 | 0.98 |
| 1slt | 1006 | 24596 | 327 | 8 | 1(0) | 1(0) | 3(1) | 1(0) | 1(0) | 0(0) | 0(0) | 1(0) | 0(0) | 0(0) | 65 | 30 | 419 | 1.04 |
| 1smn | 1847 | 43882 | 537 | 13 | 6(5) | 1(1) | 1(0) | 1(1) | 1(0) | 1(1) | 0(0) | 0(0) | 1(1) | 1(1) | 130 | 75 | 849 | 1.04 |
| 1sox | 3613 | 86048 | 1412 | 37 | 21(12) | 8(2) | 3(1) | 1(0) | 0(0) | 0(0) | 0(0) | 1(1) | 1(1) | 2(2) | 260 | 129 | 1535 | 1 |
| 1tc1 | 1407 | 34809 | 189 | 8 | 6(2) | 0(0) | 0(0) | 1(1) | 1(0) | 0(0) | 0(0) | 0(0) | 0(0) | 0(0) | 49 | 36 | 605 | 0.98 |
| 1tox | 3960 | 94312 | 574 | 24 | 14(0) | 6(0) | 1(0) | 2(0) | 0(0) | 0(0) | 0(0) | 0(0) | 0(0) | 1(0) | 136 | 90 | 1695 | 1.01 |
| 1trk | 5198 | 121894 | 2130 | 72 | 31(12) | 13(9) | 10(7) | 7(5) | 7(5) | 2(2) | 1(0) | 1(1) | 0(0) | 0(0) | 525 | 271 | 2373 | 1.03 |
| 1uby | 2800 | 68519 | 1008 | 31 | 14(1) | 10(0) | 3(0) | 2(0) | 0(0) | 0(0) | 0(0) | 0(0) | 0(0) | 2(1) | 231 | 112 | 1100 | 0.99 |
| 1utg | 548 | 15213 | 0 | 0 | 0(0) | 0(0) | 0(0) | 0(0) | 0(0) | 0(0) | 0(0) | 0(0) | 0(0) | 0(0) | 0 | 0 | 160 | 0.95 |
| 1vfr | 1734 | 44155 | 230 | 10 | 6(1) | 3(2) | 0(0) | 0(0) | 1(1) | 0(0) | 0(0) | 0(0) | 0(0) | 0(0) | 59 | 38 | 662 | 0.99 |
| 1vok | 1510 | 38451 | 180 | 9 | 5(4) | 2(1) | 2(0) | 0(0) | 0(0) | 0(0) | 0(0) | 0(0) | 0(0) | 0(0) | 57 | 36 | 596 | 1.03 |
| 1wtl | 825 | 20341 | 98 | 5 | 3(2) | 2(0) | 0(0) | 0(0) | 0(0) | 0(0) | 0(0) | 0(0) | 0(0) | 0(0) | 38 | 21 | 367 | 1.04 |
| 1xso | 1092 | 26740 | 45 | 3 | 3(3) | 0(0) | 0(0) | 0(0) | 0(0) | 0(0) | 0(0) | 0(0) | 0(0) | 0(0) | 14 | 11 | 470 | 0.99 |
| 2arc | 1345 | 31562 | 211 | 8 | 3(3) | 3(2) | 0(0) | 2(1) | 0(0) | 0(0) | 0(0) | 0(0) | 0(0) | 0(0) | 54 | 35 | 551 | 1 |
| 2ccy | 933 | 24592 | 40 | 3 | 3(0) | 0(0) | 0(0) | 0(0) | 0(0) | 0(0) | 0(0) | 0(0) | 0(0) | 0(0) | 15 | 13 | 291 | 0.98 |
| 2hdh | 2193 | 55385 | 216 | 10 | 4(2) | 4(1) | 2(1) | 0(0) | 0(0) | 0(0) | 0(0) | 0(0) | 0(0) | 0(0) | 56 | 41 | 826 | 0.99 |
| 2ilk | 1280 | 35624 | 57 | 1 | 0(0) | 0(0) | 0(0) | 0(0) | 1(0) | 0(0) | 0(0) | 0(0) | 0(0) | 0(0) | 13 | 7 | 292 | 0.96 |
| 2lig | 1224 | 31187 | 194 | 7 | 3(2) | 2(1) | 1(1) | 0(0) | 0(0) | 1(0) | 0(0) | 0(0) | 0(0) | 0(0) | 50 | 29 | 406 | 0.99 |
| 2mcg | 1606 | 39919 | 289 | 9 | 3(0) | 2(0) | 2(0) | 0(0) | 1(0) | 1(0) | 0(0) | 0(0) | 0(0) | 0(0) | 63 | 37 | 577 | 0.99 |
| 2nac | 2920 | 71106 | 577 | 19 | 10(5) | 3(2) | 2(1) | 1(1) | 1(1) | 0(0) | 0(0) | 0(0) | 1(1) | 1(1) | 148 | 92 | 1222 | 1.01 |
| 2ohx | 2785 | 67495 | 611 | 20 | 8(4) | 5(3) | 1(1) | 3(0) | 2(2) | 0(0) | 0(0) | 1(1) | 0(0) | 0(0) | 152 | 80 | 1290 | 1.03 |
| 2spc | 863 | 24321 | 45 | 1 | 0(0) | 0(0) | 0(0) | 1(1) | 0(0) | 0(0) | 0(0) | 0(0) | 0(0) | 0(0) | 10 | 6 | 166 | 0.84 |
| 2sqc | 4994 | 114423 | 1287 | 47 | 20(7) | 12(7) | 6(1) | 3(2) | 3(2) | 2(1) | 1(1) | 0(0) | 0(0) | 0(0) | 333 | 186 | 2332 | 0.98 |
| 2tct | 1574 | 40864 | 195 | 6 | 3(1) | 0(0) | 1(0) | 0(0) | 1(1) | 1(1) | 0(0) | 0(0) | 0(0) | 0(0) | 50 | 28 | 506 | 1.01 |
| 2tgi | 890 | 23058 | 56 | 2 | 1(1) | 0(0) | 1(1) | 0(0) | 0(0) | 0(0) | 0(0) | 0(0) | 0(0) | 0(0) | 15 | 13 | 319 | 0.96 |
| 3dap | 2467 | 60895 | 403 | 14 | 6(1) | 4(1) | 2(1) | 0(0) | 1(0) | 0(0) | 1(1) | 0(0) | 0(0) | 0(0) | 111 | 61 | 994 | 1.03 |
| 3grs | 3499 | 87534 | 378 | 18 | 13(5) | 3(3) | 1(1) | 0(0) | 0(0) | 0(0) | 0(0) | 1(1) | 0(0) | 0(0) | 107 | 72 | 1370 | 1.03 |
| 3sdh | 1147 | 28389 | 85 | 4 | 3(0) | 0(0) | 0(0) | 1(0) | 0(0) | 0(0) | 0(0) | 0(0) | 0(0) | 0(0) | 25 | 18 | 387 | 1.06 |
| 3ssi | 772 | 20021 | 151 | 5 | 0(0) | 3(2) | 1(0) | 1(0) | 0(0) | 0(0) | 0(0) | 0(0) | 0(0) | 0(0) | 36 | 21 | 262 | 0.98 |
| 4cha | 1756 | 42780 | 398 | 12 | 3(2) | 2(1) | 4(3) | 1(1) | 1(1) | 1(1) | 0(0) | 0(0) | 0(0) | 0(0) | 89 | 53 | 764 | 1.02 |
| 5csm | 2066 | 51760 | 408 | 11 | 4(1) | 3(0) | 1(0) | 0(0) | 1(0) | 0(0) | 1(1) | 0(0) | 0(0) | 1(1) | 98 | 55 | 823 | 1.02 |
| 5rub | 3330 | 79156 | 1509 | 35 | 18(4) | 4(3) | 4(2) | 2(2) | 2(1) | 2(2) | 0(0) | 0(0) | 0(0) | 3(3) | 283 | 147 | 1504 | 1 |
| 8prk | 2212 | 52635 | 499 | 22 | 11(10) | 8(3) | 0(0) | 2(2) | 1(0) | 0(0) | 0(0) | 0(0) | 0(0) | 0(0) | 139 | 77 | 1002 | 1.01 |
| 9wga | 1159 | 29585 | 104 | 5 | 3(2) | 1(1) | 1(1) | 0(0) | 0(0) | 0(0) | 0(0) | 0(0) | 0(0) | 0(0) | 29 | 21 | 329 | 0.91 |
| 1af5 | 1030 | 26855 | 107 | 4 | 1 | 1 | 2 | 0 | 0 | 0 | 0 | 0 | 0 | 0 | 35 | 21 | 368 | 1.01 |
| 1csh | 3391 | 82160 | 1203 | 36 | 18 | 8 | 2 | 2 | 1 | 0 | 2 | 1 | 0 | 2 | 195 | 109 | 1480 | 1 |
| 1hhp | 758 | 20548 | 0 | 0 | 0 | 0 | 0 | 0 | 0 | 0 | 0 | 0 | 0 | 0 | 0 | 0 | 243 | 1 |
| 1lyn | 1048 | 27253 | 58 | 3 | 2 | 0 | 1 | 0 | 0 | 0 | 0 | 0 | 0 | 0 | 16 | 11 | 400 | 0.97 |
| 1pre | 3523 | 85758 | 732 | 21 | 11 | 3 | 1 | 1 | 1 | 0 | 3 | 0 | 0 | 1 | 173 | 97 | 1351 | 1 |
| 1rfb | 990 | 28645 | 152 | 6 | 3 | 1 | 0 | 2 | 0 | 0 | 0 | 0 | 0 | 0 | 33 | 18 | 173 | 0.87 |
| 1smt | 779 | 21501 | 119 | 4 | 2 | 0 | 1 | 0 | 1 | 0 | 0 | 0 | 0 | 0 | 21 | 12 | 227 | 0.99 |
| 4kbh | 3494 | 79040 | 1579 | 37 | 17 | 8 | 2 | 1 | 1 | 3 | 0 | 0 | 0 | 5 | 313 | 153 | 1802 | 1 |
| **(b) Inter_H** | | | | | | | | | | | | | | | | | | |
| 12as | 379 |  | 281 | 9 | 3(1) | 2(0) | 0(0) | 3(2) | 1(1) | 0(0) | 0(0) | 0(0) | 0(0) | 0(0) | 70 | 43 | 97 | 1.07 |
| 1a3c | 192 |  | 46 | 2 | 0(0) | 2(2) | 0(0) | 0(0) | 0(0) | 0(0) | 0(0) | 0(0) | 0(0) | 0(0) | 14 | 10 | 88 | 1.04 |
| 1a4i | 279 |  | 283 | 3 | 1(1) | 0(0) | 0(0) | 1(1) | 0(0) | 0(0) | 0(0) | 0(0) | 0(0) | 1(1) | 49 | 27 | 99 | 1.06 |
| 1a4u | 472 |  | 115 | 1 | 0(0) | 0(0) | 0(0) | 0(0) | 0(0) | 0(0) | 0(0) | 0(0) | 0(0) | 1(1) | 25 | 8 | 192 | 1.05 |
| 1aa7 | 248 |  | 244 | 8 | 2(1) | 3(2) | 0(0) | 2(0) | 1(0) | 0(0) | 0(0) | 0(0) | 0(0) | 0(0) | 60 | 27 | 67 | 1.02 |
| 1ad3 | 774 |  | 192 | 9 | 4(2) | 3(3) | 2(2) | 0(0) | 0(0) | 0(0) | 0(0) | 0(0) | 0(0) | 0(0) | 55 | 42 | 334 | 1.03 |
| 1ade | 629 |  | 1700 | 11 | 4(2) | 1(0) | 0(0) | 0(0) | 0(0) | 0(0) | 1(1) | 0(0) | 1(1) | 4(4) | 193 | 82 | 127 | 1.01 |
| 1afw | 517 |  | 549 | 9 | 2(0) | 2(0) | 0(0) | 0(0) | 0(0) | 2(2) | 1(1) | 0(0) | 1(1) | 1(1) | 101 | 57 | 193 | 1.02 |
| 1ajs | 699 |  | 378 | 6 | 2(1) | 0(0) | 2(2) | 0(0) | 0(0) | 0(0) | 1(1) | 0(0) | 0(0) | 1(1) | 87 | 48 | 255 | 1.01 |
| 1alo | 278 |  | 266 | 4 | 2(2) | 0(0) | 0(0) | 0(0) | 0(0) | 0(0) | 0(0) | 0(0) | 0(0) | 2(2) | 50 | 26 | 60 | 1.02 |
| 1amk | 318 |  | 358 | 5 | 2(2) | 0(0) | 0(0) | 0(0) | 0(0) | 2(2) | 0(0) | 0(0) | 0(0) | 1(1) | 66 | 30 | 132 | 1.04 |
| 1aor | 251 |  | 89 | 4 | 2(2) | 1(0) | 1(0) | 0(0) | 0(0) | 0(0) | 0(0) | 0(0) | 0(0) | 0(0) | 25 | 16 | 77 | 0.99 |
| 1aq6 | 449 |  | 73 | 5 | 5(1) | 0(0) | 0(0) | 0(0) | 0(0) | 0(0) | 0(0) | 0(0) | 0(0) | 0(0) | 25 | 19 | 273 | 1.06 |
| 1auo | 154 |  | 15 | 1 | 1(0) | 0(0) | 0(0) | 0(0) | 0(0) | 0(0) | 0(0) | 0(0) | 0(0) | 0(0) | 5 | 5 | 57 | 0.93 |
| 1b3a | 171 |  | 13 | 1 | 1(1) | 0(0) | 0(0) | 0(0) | 0(0) | 0(0) | 0(0) | 0(0) | 0(0) | 0(0) | 5 | 5 | 62 | 1.01 |
| 1b5e | 524 |  | 508 | 12 | 5(2) | 1(1) | 4(2) | 0(0) | 0(0) | 0(0) | 0(0) | 0(0) | 0(0) | 2(2) | 94 | 48 | 183 | 0.98 |
| 1b67 | 326 |  | 32 | 2 | 1(0) | 1(1) | 0(0) | 0(0) | 0(0) | 0(0) | 0(0) | 0(0) | 0(0) | 0(0) | 10 | 8 | 140 | 1.1 |
| 1b8a | 996 |  | 730 | 13 | 7(5) | 0(0) | 1(1) | 0(0) | 0(0) | 0(0) | 0(0) | 0(0) | 1(1) | 4(4) | 155 | 72 | 445 | 1.06 |
| 1b8j | 836 |  | 1453 | 19 | 2(2) | 7(7) | 1(1) | 1(1) | 0(0) | 3(2) | 1(1) | 0(0) | 0(0) | 4(4) | 264 | 122 | 306 | 1.01 |
| 1bam | 152 |  | 0 | 0 | 0(0) | 0(0) | 0(0) | 0(0) | 0(0) | 0(0) | 0(0) | 0(0) | 0(0) | 0(0) | 0 | 0 | 60 | 1.03 |
| 1bbh | 171 |  | 19 | 1 | 1(0) | 0(0) | 0(0) | 0(0) | 0(0) | 0(0) | 0(0) | 0(0) | 0(0) | 0(0) | 7 | 6 | 79 | 1.08 |
| 1bd0 | 655 |  | 249 | 8 | 5(4) | 1(1) | 0(0) | 0(0) | 0(0) | 0(0) | 2(2) | 0(0) | 0(0) | 0(0) | 71 | 46 | 184 | 1.04 |
| 1bif | 194 |  | 20 | 1 | 0(0) | 1(0) | 0(0) | 0(0) | 0(0) | 0(0) | 0(0) | 0(0) | 0(0) | 0(0) | 6 | 4 | 42 | 0.86 |
| 1biq | 618 |  | 501 | 13 | 4(1) | 4(3) | 1(1) | 0(0) | 0(0) | 2(1) | 0(0) | 1(1) | 1(1) | 0(0) | 117 | 61 | 218 | 1 |
| 1bis | 350 |  | 557 | 7 | 6(5) | 0(0) | 0(0) | 0(0) | 0(0) | 0(0) | 0(0) | 0(0) | 0(0) | 1(1) | 98 | 44 | 92 | 0.94 |
| 1bjw | 662 |  | 643 | 12 | 4(1) | 0(0) | 1(0) | 3(2) | 1(1) | 0(0) | 0(0) | 0(0) | 1(1) | 2(2) | 138 | 62 | 263 | 1 |
| 1bkp | 472 |  | 279 | 5 | 2(2) | 0(0) | 1(1) | 0(0) | 0(0) | 0(0) | 0(0) | 0(0) | 0(0) | 2(2) | 68 | 31 | 144 | 1.03 |
| 1bmd | 323 |  | 77 | 5 | 4(3) | 1(1) | 0(0) | 0(0) | 0(0) | 0(0) | 0(0) | 0(0) | 0(0) | 0(0) | 21 | 18 | 135 | 1 |
| 1brw | 235 |  | 0 | 0 | 0(0) | 0(0) | 0(0) | 0(0) | 0(0) | 0(0) | 0(0) | 0(0) | 0(0) | 0(0) | 0 | 0 | 113 | 1.03 |
| 1bsl | 425 |  | 530 | 9 | 3(1) | 2(0) | 1(1) | 0(0) | 0(0) | 0(0) | 0(0) | 1(1) | 1(1) | 1(1) | 96 | 51 | 188 | 1.02 |
| 1bsr | 401 |  | 180 | 2 | 1(0) | 0(0) | 0(0) | 0(0) | 0(0) | 0(0) | 0(0) | 0(0) | 0(0) | 1(1) | 32 | 19 | 150 | 1.03 |
| 1buo | 390 |  | 330 | 4 | 0(0) | 3(1) | 0(0) | 0(0) | 0(0) | 0(0) | 0(0) | 0(0) | 0(0) | 1(1) | 52 | 20 | 136 | 1.03 |
| 1bxg | 276 |  | 577 | 5 | 2(0) | 1(1) | 0(0) | 0(0) | 0(0) | 0(0) | 0(0) | 0(0) | 0(0) | 2(2) | 88 | 35 | 68 | 1.1 |
| 1bxk | 273 |  | 13 | 1 | 1(0) | 0(0) | 0(0) | 0(0) | 0(0) | 0(0) | 0(0) | 0(0) | 0(0) | 0(0) | 5 | 5 | 112 | 1.12 |
| 1cdc | 777 |  | 49 | 2 | 0(0) | 2(0) | 0(0) | 0(0) | 0(0) | 0(0) | 0(0) | 0(0) | 0(0) | 0(0) | 16 | 10 | 340 | 1.06 |
| 1cg2 | 272 |  | 240 | 2 | 0(0) | 1(1) | 0(0) | 0(0) | 0(0) | 0(0) | 0(0) | 0(0) | 0(0) | 1(0) | 39 | 24 | 101 | 1.04 |
| 1chm | 790 |  | 1962 | 8 | 3(2) | 1(1) | 3(3) | 0(0) | 0(0) | 0(0) | 0(0) | 0(0) | 0(0) | 1(1) | 251 | 92 | 231 | 0.99 |
| 1cmb | 324 |  | 184 | 7 | 4(1) | 1(0) | 0(0) | 1(0) | 0(0) | 1(0) | 0(0) | 0(0) | 0(0) | 0(0) | 37 | 20 | 130 | 1.06 |
| 1cnz | 493 |  | 21 | 1 | 0(0) | 1(0) | 0(0) | 0(0) | 0(0) | 0(0) | 0(0) | 0(0) | 0(0) | 0(0) | 6 | 6 | 219 | 1.04 |
| 1coz | 216 |  | 87 | 1 | 0(0) | 0(0) | 0(0) | 0(0) | 0(0) | 0(0) | 0(0) | 1(1) | 0(0) | 0(0) | 16 | 6 | 88 | 1.04 |
| 1ctt | 470 |  | 1143 | 7 | 0(0) | 2(2) | 0(0) | 2(2) | 0(0) | 0(0) | 0(0) | 0(0) | 0(0) | 3(3) | 184 | 76 | 156 | 1 |
| 1cvu | 542 |  | 120 | 6 | 4(2) | 1(0) | 1(0) | 0(0) | 0(0) | 0(0) | 0(0) | 0(0) | 0(0) | 0(0) | 39 | 27 | 153 | 1.01 |
| 1czj | 160 |  | 24 | 2 | 2(0) | 0(0) | 0(0) | 0(0) | 0(0) | 0(0) | 0(0) | 0(0) | 0(0) | 0(0) | 8 | 6 | 42 | 0.92 |
| 1daa | 467 |  | 160 | 6 | 2(1) | 2(2) | 1(1) | 1(1) | 0(0) | 0(0) | 0(0) | 0(0) | 0(0) | 0(0) | 49 | 26 | 221 | 1.08 |
| 1dor | 441 |  | 278 | 6 | 3(2) | 0(0) | 0(0) | 2(2) | 0(0) | 0(0) | 0(0) | 0(0) | 0(0) | 1(1) | 50 | 28 | 137 | 1.08 |
| 1dpg | 458 |  | 397 | 12 | 4(2) | 3(0) | 2(2) | 0(0) | 1(1) | 0(0) | 0(0) | 2(2) | 0(0) | 0(0) | 86 | 60 | 174 | 1.11 |
| 1dqs | 346 |  | 99 | 7 | 7(5) | 0(0) | 0(0) | 0(0) | 0(0) | 0(0) | 0(0) | 0(0) | 0(0) | 0(0) | 21 | 17 | 115 | 1.08 |
| 1dxg | 146 |  | 40 | 2 | 1(0) | 1(0) | 0(0) | 0(0) | 0(0) | 0(0) | 0(0) | 0(0) | 0(0) | 0(0) | 12 | 8 | 65 | 1.02 |
| 1e98 | 172 |  | 86 | 3 | 0(0) | 1(0) | 2(2) | 0(0) | 0(0) | 0(0) | 0(0) | 0(0) | 0(0) | 0(0) | 34 | 12 | 74 | 0.95 |
| 1ebh | 402 |  | 165 | 5 | 2(1) | 1(0) | 0(0) | 0(0) | 2(2) | 0(0) | 0(0) | 0(0) | 0(0) | 0(0) | 41 | 26 | 153 | 1 |
| 1f13 | 570 |  | 29 | 2 | 2(1) | 0(0) | 0(0) | 0(0) | 0(0) | 0(0) | 0(0) | 0(0) | 0(0) | 0(0) | 6 | 5 | 141 | 1.01 |
| 1fip | 375 |  | 98 | 2 | 0(0) | 0(0) | 0(0) | 1(0) | 1(0) | 0(0) | 0(0) | 0(0) | 0(0) | 0(0) | 23 | 12 | 157 | 1.04 |
| 1fro | 727 |  | 392 | 8 | 6(2) | 0(0) | 0(0) | 0(0) | 0(0) | 0(0) | 0(0) | 0(0) | 0(0) | 2(2) | 76 | 42 | 271 | 1.05 |
| 1gvp | 180 |  | 95 | 4 | 2(0) | 0(0) | 2(0) | 0(0) | 0(0) | 0(0) | 0(0) | 0(0) | 0(0) | 0(0) | 30 | 16 | 60 | 1.1 |
| 1hjr | 203 |  | 14 | 1 | 1(0) | 0(0) | 0(0) | 0(0) | 0(0) | 0(0) | 0(0) | 0(0) | 0(0) | 0(0) | 6 | 5 | 88 | 1.02 |
| 1hss | 220 |  | 141 | 4 | 2(1) | 0(0) | 0(0) | 1(1) | 0(0) | 1(0) | 0(0) | 0(0) | 0(0) | 0(0) | 28 | 18 | 50 | 0.99 |
| 1hxp | 712 |  | 128 | 8 | 7(0) | 1(0) | 0(0) | 0(0) | 0(0) | 0(0) | 0(0) | 0(0) | 0(0) | 0(0) | 34 | 28 | 304 | 1.03 |
| 1icw | 204 |  | 14 | 1 | 1(0) | 0(0) | 0(0) | 0(0) | 0(0) | 0(0) | 0(0) | 0(0) | 0(0) | 0(0) | 5 | 5 | 74 | 1.12 |
| 1imb | 320 |  | 75 | 3 | 0(0) | 3(2) | 0(0) | 0(0) | 0(0) | 0(0) | 0(0) | 0(0) | 0(0) | 0(0) | 28 | 13 | 104 | 1.06 |
| 1isa | 190 |  | 79 | 3 | 1(1) | 1(1) | 1(0) | 0(0) | 0(0) | 0(0) | 0(0) | 0(0) | 0(0) | 0(0) | 23 | 14 | 46 | 1.02 |
| 1ivy | 370 |  | 514 | 8 | 1(1) | 1(0) | 0(0) | 1(1) | 1(1) | 0(0) | 1(0) | 1(1) | 1(1) | 1(1) | 105 | 45 | 78 | 1.09 |
| 1jhg | 438 |  | 245 | 5 | 2(2) | 0(0) | 0(0) | 2(0) | 0(0) | 0(0) | 0(0) | 0(0) | 0(0) | 1(0) | 48 | 32 | 140 | 1.06 |
| 1jsg | 160 |  | 0 | 0 | 0(0) | 0(0) | 0(0) | 0(0) | 0(0) | 0(0) | 0(0) | 0(0) | 0(0) | 0(0) | 0 | 0 | 58 | 1.06 |
| 1kba | 103 |  | 0 | 0 | 0(0) | 0(0) | 0(0) | 0(0) | 0(0) | 0(0) | 0(0) | 0(0) | 0(0) | 0(0) | 0 | 0 | 29 | 1.11 |
| 1kpf | 390 |  | 83 | 1 | 0(0) | 0(0) | 0(0) | 0(0) | 0(0) | 0(0) | 0(0) | 1(0) | 0(0) | 0(0) | 18 | 10 | 194 | 1.02 |
| 1m6p | 217 |  | 15 | 1 | 1(1) | 0(0) | 0(0) | 0(0) | 0(0) | 0(0) | 0(0) | 0(0) | 0(0) | 0(0) | 5 | 4 | 41 | 1.12 |
| 1mkb | 409 |  | 1142 | 5 | 1(0) | 1(1) | 1(1) | 0(0) | 0(0) | 0(0) | 0(0) | 0(0) | 0(0) | 2(2) | 162 | 66 | 110 | 1.08 |
| 1mor | 650 |  | 2282 | 5 | 2(0) | 2(2) | 0(0) | 0(0) | 0(0) | 0(0) | 0(0) | 0(0) | 0(0) | 1(1) | 218 | 84 | 202 | 1.01 |
| 1nox | 616 |  | 264 | 4 | 0(0) | 0(0) | 0(0) | 0(0) | 0(0) | 4(4) | 0(0) | 0(0) | 0(0) | 0(0) | 52 | 28 | 228 | 1.05 |
| 1nse | 566 |  | 387 | 4 | 1(0) | 0(0) | 1(1) | 0(0) | 0(0) | 0(0) | 0(0) | 0(0) | 0(0) | 2(1) | 66 | 31 | 154 | 0.97 |
| 1nsy | 535 |  | 355 | 10 | 5(2) | 1(0) | 1(0) | 1(1) | 1(0) | 0(0) | 0(0) | 0(0) | 0(0) | 1(0) | 69 | 41 | 181 | 0.99 |
| 1oac | 1566 |  | 3352 | 26 | 8(5) | 10(10) | 1(1) | 0(0) | 1(0) | 1(1) | 0(0) | 2(2) | 0(0) | 3(3) | 395 | 175 | 565 | 1.01 |
| 1opy | 228 |  | 0 | 0 | 0(0) | 0(0) | 0(0) | 0(0) | 0(0) | 0(0) | 0(0) | 0(0) | 0(0) | 0(0) | 0 | 0 | 126 | 1.02 |
| 1pgt | 237 |  | 34 | 2 | 2(2) | 0(0) | 0(0) | 0(0) | 0(0) | 0(0) | 0(0) | 0(0) | 0(0) | 0(0) | 12 | 8 | 102 | 1.08 |
| 1qfh | 497 |  | 266 | 7 | 3(2) | 2(1) | 0(0) | 0(0) | 0(0) | 1(1) | 0(0) | 0(0) | 0(0) | 1(1) | 57 | 32 | 163 | 1.09 |
| 1qhi | 342 |  | 143 | 8 | 6(0) | 2(0) | 0(0) | 0(0) | 0(0) | 0(0) | 0(0) | 0(0) | 0(0) | 0(0) | 51 | 29 | 131 | 1.08 |
| 1qr2 | 432 |  | 380 | 5 | 1(1) | 0(0) | 1(1) | 1(1) | 1(1) | 0(0) | 0(0) | 0(0) | 0(0) | 1(1) | 70 | 42 | 142 | 1.03 |
| 1r2f | 378 |  | 308 | 9 | 3(2) | 3(1) | 2(0) | 0(0) | 0(0) | 0(0) | 0(0) | 0(0) | 0(0) | 1(1) | 67 | 41 | 128 | 1.01 |
| 1reg | 144 |  | 126 | 3 | 1(0) | 0(0) | 0(0) | 1(1) | 0(0) | 1(1) | 0(0) | 0(0) | 0(0) | 0(0) | 28 | 14 | 39 | 0.95 |
| 1rpo | 256 |  | 59 | 2 | 1(0) | 0(0) | 1(0) | 0(0) | 0(0) | 0(0) | 0(0) | 0(0) | 0(0) | 0(0) | 18 | 10 | 94 | 1.1 |
| 1ses | 459 |  | 422 | 6 | 4(0) | 0(0) | 0(0) | 0(0) | 0(0) | 0(0) | 0(0) | 0(0) | 0(0) | 2(2) | 79 | 31 | 219 | 0.99 |
| 1slt | 121 |  | 152 | 1 | 0(0) | 0(0) | 0(0) | 0(0) | 0(0) | 0(0) | 0(0) | 0(0) | 0(0) | 1(0) | 20 | 12 | 25 | 1.1 |
| 1smn | 174 |  | 91 | 3 | 1(0) | 0(0) | 1(1) | 1(1) | 0(0) | 0(0) | 0(0) | 0(0) | 0(0) | 0(0) | 23 | 10 | 58 | 1.01 |
| 1sox | 332 |  | 863 | 6 | 1(1) | 0(0) | 0(0) | 0(0) | 1(1) | 0(0) | 2(2) | 0(0) | 0(0) | 2(2) | 114 | 45 | 85 | 1.07 |
| 1tc1 | 305 |  | 139 | 4 | 1(0) | 1(0) | 0(0) | 1(1) | 1(1) | 0(0) | 0(0) | 0(0) | 0(0) | 0(0) | 36 | 25 | 99 | 1.07 |
| 1tox | 768 |  | 527 | 12 | 10(0) | 0(0) | 0(0) | 0(0) | 0(0) | 0(0) | 0(0) | 0(0) | 0(0) | 2(0) | 122 | 66 | 164 | 1.03 |
| 1trk | 988 |  | 1693 | 23 | 11(10) | 0(0) | 0(0) | 0(0) | 2(2) | 3(3) | 0(0) | 0(0) | 0(0) | 7(7) | 301 | 133 | 301 | 1 |
| 1uby | 448 |  | 51 | 2 | 0(0) | 2(0) | 0(0) | 0(0) | 0(0) | 0(0) | 0(0) | 0(0) | 0(0) | 0(0) | 18 | 10 | 2 | 1.47 |
| 1utg | 286 |  | 640 | 5 | 2(2) | 2(0) | 0(0) | 0(0) | 0(0) | 0(0) | 0(0) | 0(0) | 0(0) | 1(1) | 72 | 38 | 108 | 1.08 |
| 1vfr | 682 |  | 458 | 8 | 2(1) | 2(2) | 0(0) | 1(1) | 0(0) | 1(1) | 0(0) | 0(0) | 0(0) | 2(2) | 95 | 47 | 237 | 1.06 |
| 1vok | 318 |  | 159 | 2 | 0(0) | 1(0) | 0(0) | 0(0) | 0(0) | 0(0) | 0(0) | 0(0) | 0(0) | 1(0) | 30 | 13 | 72 | 1 |
| 1wtl | 163 |  | 30 | 1 | 0(0) | 1(1) | 0(0) | 0(0) | 0(0) | 0(0) | 0(0) | 0(0) | 0(0) | 0(0) | 9 | 6 | 62 | 1.01 |
| 1xso | 148 |  | 0 | 0 | 0(0) | 0(0) | 0(0) | 0(0) | 0(0) | 0(0) | 0(0) | 0(0) | 0(0) | 0(0) | 0 | 0 | 66 | 0.99 |
| 2arc | 168 |  | 56 | 3 | 2(1) | 1(1) | 0(0) | 0(0) | 0(0) | 0(0) | 0(0) | 0(0) | 0(0) | 0(0) | 15 | 10 | 60 | 0.99 |
| 2ccy | 163 |  | 86 | 2 | 1(1) | 0(0) | 0(0) | 0(0) | 0(0) | 0(0) | 1(1) | 0(0) | 0(0) | 0(0) | 17 | 7 | 74 | 1.03 |
| 2hdh | 337 |  | 764 | 6 | 1(1) | 1(1) | 1(1) | 0(0) | 0(0) | 0(0) | 0(0) | 0(0) | 0(0) | 3(3) | 112 | 39 | 81 | 1.06 |
| 2ilk | 888 |  | 190 | 6 | 4(0) | 0(0) | 0(0) | 0(0) | 2(2) | 0(0) | 0(0) | 0(0) | 0(0) | 0(0) | 48 | 32 | 420 | 1.01 |
| 2lig | 349 |  | 44 | 3 | 3(1) | 0(0) | 0(0) | 0(0) | 0(0) | 0(0) | 0(0) | 0(0) | 0(0) | 0(0) | 5 | 5 | 117 | 0.96 |
| 2mcg | 354 |  | 134 | 4 | 1(1) | 0(0) | 2(1) | 1(0) | 0(0) | 0(0) | 0(0) | 0(0) | 0(0) | 0(0) | 36 | 24 | 78 | 1.1 |
| 2nac | 769 |  | 397 | 15 | 3(3) | 9(5) | 1(1) | 0(0) | 2(2) | 0(0) | 0(0) | 0(0) | 0(0) | 0(0) | 99 | 67 | 368 | 1.02 |
| 2ohx | 356 |  | 121 | 8 | 7(2) | 1(1) | 0(0) | 0(0) | 0(0) | 0(0) | 0(0) | 0(0) | 0(0) | 0(0) | 36 | 29 | 147 | 0.98 |
| 2spc | 496 |  | 185 | 3 | 0(0) | 1(1) | 0(0) | 0(0) | 0(0) | 0(0) | 1(1) | 1(0) | 0(0) | 0(0) | 43 | 20 | 184 | 1 |
| 2sqc | 162 |  | 130 | 2 | 0(0) | 1(0) | 0(0) | 0(0) | 0(0) | 0(0) | 0(0) | 0(0) | 0(0) | 1(1) | 26 | 12 | 41 | 1.09 |
| 2tct | 546 |  | 72 | 4 | 3(2) | 1(0) | 0(0) | 0(0) | 0(0) | 0(0) | 0(0) | 0(0) | 0(0) | 0(0) | 26 | 18 | 174 | 0.99 |
| 2tgi | 248 |  | 0 | 0 | 0(0) | 0(0) | 0(0) | 0(0) | 0(0) | 0(0) | 0(0) | 0(0) | 0(0) | 0(0) | 0 | 0 | 76 | 1.08 |
| 3dap | 559 |  | 461 | 8 | 5(2) | 0(0) | 1(1) | 0(0) | 0(0) | 0(0) | 1(1) | 0(0) | 0(0) | 1(1) | 87 | 38 | 225 | 1.05 |
| 3grs | 666 |  | 318 | 8 | 2(2) | 2(2) | 0(0) | 2(2) | 0(0) | 0(0) | 0(0) | 2(2) | 0(0) | 0(0) | 74 | 48 | 278 | 1.06 |
| 3sdh | 175 |  | 93 | 4 | 2(0) | 1(0) | 1(0) | 0(0) | 0(0) | 0(0) | 0(0) | 0(0) | 0(0) | 0(0) | 20 | 14 | 57 | 1.16 |
| 3ssi | 183 |  | 61 | 4 | 4(0) | 0(0) | 0(0) | 0(0) | 0(0) | 0(0) | 0(0) | 0(0) | 0(0) | 0(0) | 26 | 18 | 75 | 1.17 |
| 4cha | 212 |  | 0 | 0 | 0(0) | 0(0) | 0(0) | 0(0) | 0(0) | 0(0) | 0(0) | 0(0) | 0(0) | 0(0) | 0 | 0 | 42 | 0.96 |
| 5csm | 408 |  | 284 | 9 | 1(0) | 2(2) | 4(0) | 2(0) | 0(0) | 0(0) | 0(0) | 0(0) | 0(0) | 0(0) | 60 | 42 | 116 | 1.08 |
| 5rub | 672 |  | 1647 | 10 | 5(2) | 1(1) | 0(0) | 0(0) | 0(0) | 0(0) | 0(0) | 0(0) | 0(0) | 4(3) | 191 | 83 | 235 | 1.08 |
| 8prk | 236 |  | 166 | 2 | 0(0) | 1(1) | 0(0) | 0(0) | 0(0) | 0(0) | 0(0) | 0(0) | 0(0) | 1(1) | 27 | 16 | 73 | 0.93 |
| 9wga | 478 |  | 43 | 3 | 3(1) | 0(0) | 0(0) | 0(0) | 0(0) | 0(0) | 0(0) | 0(0) | 0(0) | 0(0) | 16 | 12 | 136 | 1 |
| 1af5 | 194 |  | 0 | 0 | 0 | 0 | 0 | 0 | 0 | 0 | 0 | 0 | 0 | 0 | 0 | 0 | 84 | 0.98 |
| 1csh | 1015 |  | 765 | 9 | 4 | 0 | 0 | 0 | 0 | 0 | 1 | 0 | 0 | 4 | 97 | 39 | 420 | 1.06 |
| 1hhp | 328 |  | 0 | 0 | 0 | 0 | 0 | 0 | 0 | 0 | 0 | 0 | 0 | 0 | 0 | 0 | 134 | 1.02 |
| 1lyn | 192 |  | 410 | 3 | 1 | 1 | 0 | 0 | 0 | 0 | 0 | 0 | 0 | 1 | 48 | 21 | 58 | 1 |
| 1pre | 516 |  | 63 | 3 | 1 | 2 | 0 | 0 | 0 | 0 | 0 | 0 | 0 | 0 | 15 | 9 | 102 | 0.97 |
| 1rfb | 565 |  | 382 | 6 | 2 | 1 | 1 | 0 | 0 | 0 | 0 | 0 | 0 | 2 | 63 | 29 | 58 | 0.98 |
| 1smt | 392 |  | 15 | 1 | 1 | 0 | 0 | 0 | 0 | 0 | 0 | 0 | 0 | 0 | 5 | 4 | 171 | 1.03 |
| 4kbh | 289 |  | 79 | 1 | 0 | 0 | 0 | 0 | 0 | 0 | 1 | 0 | 0 | 0 | 19 | 8 | 52 | 1.04 |
| **(c) Inter_C** | | | | | | | | | | | | | | | | | | |
| 1A2Y | 152 |  | 18 | 1 | 1(1) | 0(0) | 0(0) | 0(0) | 0(0) | 0(0) | 0(0) | 0(0) | 0(0) | 0(0) | 4 | 4 | 26 | 0.97 |
| 1ACB | 104 |  | 19 | 1 | 1(0) | 0(0) | 0(0) | 0(0) | 0(0) | 0(0) | 0(0) | 0(0) | 0(0) | 0(0) | 5 | 5 | 23 | 0.92 |
| 1AVA | 289 |  | 259 | 4 | 3(3) | 0(0) | 0(0) | 0(0) | 0(0) | 0(0) | 0(0) | 0(0) | 0(0) | 1(1) | 50 | 24 | 66 | 1.03 |
| 1AVW | 122 |  | 15 | 1 | 1(0) | 0(0) | 0(0) | 0(0) | 0(0) | 0(0) | 0(0) | 0(0) | 0(0) | 0(0) | 5 | 5 | 42 | 0.94 |
| 1AXI | 295 |  | 658 | 7 | 3(2) | 1(1) | 0(0) | 0(0) | 0(0) | 0(0) | 1(1) | 0(0) | 0(0) | 2(2) | 100 | 35 | 62 | 1.01 |
| 1AY7 | 134 |  | 0 | 0 | 0(0) | 0(0) | 0(0) | 0(0) | 0(0) | 0(0) | 0(0) | 0(0) | 0(0) | 0(0) | 0 | 0 | 40 | 0.94 |
| 1B0N | 310 |  | 54 | 3 | 2(1) | 1(1) | 0(0) | 0(0) | 0(0) | 0(0) | 0(0) | 0(0) | 0(0) | 0(0) | 17 | 12 | 117 | 1.01 |
| 1BJ1 | 172 |  | 14 | 1 | 1(0) | 0(0) | 0(0) | 0(0) | 0(0) | 0(0) | 0(0) | 0(0) | 0(0) | 0(0) | 7 | 5 | 104 | 1 |
| 1BLX | 201 |  | 121 | 1 | 0(0) | 0(0) | 0(0) | 0(0) | 0(0) | 0(0) | 0(0) | 0(0) | 0(0) | 1(1) | 22 | 11 | 61 | 1.05 |
| 1C1Y | 136 |  | 43 | 3 | 3(1) | 0(0) | 0(0) | 0(0) | 0(0) | 0(0) | 0(0) | 0(0) | 0(0) | 0(0) | 10 | 9 | 42 | 1.01 |
| 1CA0 | 153 |  | 69 | 2 | 1(1) | 0(0) | 0(0) | 0(0) | 1(1) | 0(0) | 0(0) | 0(0) | 0(0) | 0(0) | 14 | 13 | 74 | 0.94 |
| 1CHO | 97 |  | 49 | 2 | 1(0) | 0(0) | 1(0) | 0(0) | 0(0) | 0(0) | 0(0) | 0(0) | 0(0) | 0(0) | 9 | 7 | 25 | 0.94 |
| 1CLV | 234 |  | 557 | 8 | 3(2) | 0(0) | 0(0) | 0(0) | 2(2) | 0(0) | 0(0) | 1(1) | 0(0) | 2(2) | 87 | 42 | 72 | 1.05 |
| 1CSE | 90 |  | 0 | 0 | 0(0) | 0(0) | 0(0) | 0(0) | 0(0) | 0(0) | 0(0) | 0(0) | 0(0) | 0(0) | 0 | 0 | 33 | 1.01 |
| 1CXZ | 184 |  | 163 | 3 | 1(0) | 1(0) | 0(0) | 0(0) | 0(0) | 0(0) | 0(0) | 0(0) | 0(0) | 1(1) | 28 | 15 | 27 | 1 |
| 1CZY | 100 |  | 0 | 0 | 0(0) | 0(0) | 0(0) | 0(0) | 0(0) | 0(0) | 0(0) | 0(0) | 0(0) | 0(0) | 0 | 0 | 35 | 1 |
| 1D4V | 149 |  | 13 | 1 | 1(1) | 0(0) | 0(0) | 0(0) | 0(0) | 0(0) | 0(0) | 0(0) | 0(0) | 0(0) | 4 | 2 | 26 | 1.07 |
| 1DF9 | 274 |  | 0 | 0 | 0(0) | 0(0) | 0(0) | 0(0) | 0(0) | 0(0) | 0(0) | 0(0) | 0(0) | 0(0) | 0 | 0 | 98 | 0.99 |
| 1DHK | 233 |  | 404 | 3 | 0(0) | 0(0) | 0(0) | 0(0) | 1(1) | 0(0) | 0(0) | 0(0) | 0(0) | 2(2) | 65 | 33 | 57 | 0.94 |
| 1DS6 | 239 |  | 40 | 2 | 1(0) | 1(0) | 0(0) | 0(0) | 0(0) | 0(0) | 0(0) | 0(0) | 0(0) | 0(0) | 11 | 9 | 67 | 1.07 |
| 1DTD | 169 |  | 154 | 3 | 2(1) | 0(0) | 0(0) | 0(0) | 0(0) | 0(0) | 0(0) | 0(0) | 0(0) | 1(1) | 34 | 23 | 46 | 1.01 |
| 1DVF | 119 |  | 0 | 0 | 0(0) | 0(0) | 0(0) | 0(0) | 0(0) | 0(0) | 0(0) | 0(0) | 0(0) | 0(0) | 0 | 0 | 27 | 0.89 |
| 1DZB | 190 |  | 43 | 2 | 1(1) | 1(0) | 0(0) | 0(0) | 0(0) | 0(0) | 0(0) | 0(0) | 0(0) | 0(0) | 16 | 8 | 69 | 1.05 |
| 1E44 | 293 |  | 421 | 3 | 0(0) | 1(1) | 1(1) | 0(0) | 0(0) | 0(0) | 0(0) | 0(0) | 0(0) | 1(1) | 69 | 33 | 74 | 1.08 |
| 1EAY | 129 |  | 14 | 1 | 1(0) | 0(0) | 0(0) | 0(0) | 0(0) | 0(0) | 0(0) | 0(0) | 0(0) | 0(0) | 5 | 4 | 30 | 0.99 |
| 1EER | 359 |  | 399 | 4 | 0(0) | 1(1) | 0(0) | 0(0) | 0(0) | 1(1) | 0(0) | 0(0) | 0(0) | 2(2) | 70 | 35 | 101 | 1.04 |
| 1EFN | 95 |  | 18 | 1 | 1(1) | 0(0) | 0(0) | 0(0) | 0(0) | 0(0) | 0(0) | 0(0) | 0(0) | 0(0) | 8 | 5 | 37 | 0.99 |
| 1EFU | 239 |  | 86 | 4 | 3(2) | 0(0) | 0(0) | 1(0) | 0(0) | 0(0) | 0(0) | 0(0) | 0(0) | 0(0) | 24 | 13 | 59 | 1.03 |
| 1EMV | 161 |  | 63 | 2 | 0(0) | 1(1) | 1(1) | 0(0) | 0(0) | 0(0) | 0(0) | 0(0) | 0(0) | 0(0) | 15 | 12 | 59 | 1.03 |
| 1EUV | 241 |  | 0 | 0 | 0(0) | 0(0) | 0(0) | 0(0) | 0(0) | 0(0) | 0(0) | 0(0) | 0(0) | 0(0) | 0 | 0 | 73 | 0.96 |
| 1FIN | 233 |  | 178 | 9 | 8(3) | 0(0) | 0(0) | 0(0) | 0(0) | 1(1) | 0(0) | 0(0) | 0(0) | 0(0) | 38 | 25 | 69 | 1 |
| 1FLE | 119 |  | 196 | 2 | 1(0) | 0(0) | 0(0) | 0(0) | 0(0) | 0(0) | 0(0) | 0(0) | 0(0) | 1(1) | 33 | 16 | 29 | 1.09 |
| 1FLT | 117 |  | 59 | 2 | 0(0) | 1(0) | 1(1) | 0(0) | 0(0) | 0(0) | 0(0) | 0(0) | 0(0) | 0(0) | 19 | 9 | 23 | 1.06 |
| 1FS1 | 154 |  | 62 | 2 | 1(0) | 0(0) | 0(0) | 1(0) | 0(0) | 0(0) | 0(0) | 0(0) | 0(0) | 0(0) | 14 | 11 | 61 | 1.01 |
| 1FYH | 205 |  | 172 | 4 | 2(2) | 1(1) | 0(0) | 0(0) | 0(0) | 0(0) | 0(0) | 0(0) | 0(0) | 1(1) | 21 | 12 | 68 | 1.08 |
| 1GL1 | 175 |  | 61 | 1 | 0(0) | 0(0) | 0(0) | 0(0) | 0(0) | 1(1) | 0(0) | 0(0) | 0(0) | 0(0) | 16 | 7 | 57 | 0.96 |
| 1GL4 | 209 |  | 101 | 4 | 2(1) | 1(1) | 0(0) | 1(0) | 0(0) | 0(0) | 0(0) | 0(0) | 0(0) | 0(0) | 28 | 17 | 68 | 1.05 |
| 1GUA | 93 |  | 15 | 1 | 1(0) | 0(0) | 0(0) | 0(0) | 0(0) | 0(0) | 0(0) | 0(0) | 0(0) | 0(0) | 5 | 5 | 19 | 0.89 |
| 1H1R | 347 |  | 162 | 3 | 0(0) | 1(1) | 0(0) | 1(1) | 0(0) | 0(0) | 0(0) | 1(1) | 0(0) | 0(0) | 39 | 18 | 106 | 1.01 |
| 1H2K | 276 |  | 53 | 3 | 2(1) | 1(0) | 0(0) | 0(0) | 0(0) | 0(0) | 0(0) | 0(0) | 0(0) | 0(0) | 14 | 9 | 83 | 1.02 |
| 1H2T | 339 |  | 151 | 6 | 4(2) | 0(0) | 0(0) | 1(0) | 1(1) | 0(0) | 0(0) | 0(0) | 0(0) | 0(0) | 28 | 19 | 88 | 1 |
| 1HIA | 121 |  | 173 | 3 | 1(1) | 0(0) | 1(0) | 0(0) | 0(0) | 0(0) | 0(0) | 0(0) | 0(0) | 1(1) | 41 | 20 | 23 | 0.95 |
| 1I7W | 516 |  | 129 | 5 | 2(2) | 2(1) | 0(0) | 0(0) | 1(1) | 0(0) | 0(0) | 0(0) | 0(0) | 0(0) | 37 | 23 | 159 | 1.03 |
| 1IAR | 155 |  | 56 | 2 | 1(0) | 0(0) | 1(0) | 0(0) | 0(0) | 0(0) | 0(0) | 0(0) | 0(0) | 0(0) | 13 | 10 | 46 | 0.97 |
| 1IGC | 74 |  | 16 | 1 | 1(0) | 0(0) | 0(0) | 0(0) | 0(0) | 0(0) | 0(0) | 0(0) | 0(0) | 0(0) | 5 | 5 | 21 | 1.17 |
| 1J2J | 119 |  | 41 | 3 | 3(2) | 0(0) | 0(0) | 0(0) | 0(0) | 0(0) | 0(0) | 0(0) | 0(0) | 0(0) | 9 | 8 | 35 | 1.06 |
| 1JDH | 331 |  | 40 | 3 | 3(3) | 0(0) | 0(0) | 0(0) | 0(0) | 0(0) | 0(0) | 0(0) | 0(0) | 0(0) | 14 | 14 | 113 | 1.06 |
| 1JDP | 270 |  | 238 | 5 | 3(1) | 0(0) | 1(1) | 0(0) | 0(0) | 0(0) | 0(0) | 0(0) | 0(0) | 1(1) | 54 | 28 | 54 | 1.04 |
| 1JIW | 221 |  | 300 | 3 | 1(1) | 0(0) | 0(0) | 0(0) | 0(0) | 0(0) | 0(0) | 0(0) | 0(0) | 2(2) | 54 | 26 | 71 | 1.01 |
| 1JTG | 292 |  | 268 | 7 | 2(2) | 4(3) | 0(0) | 0(0) | 0(0) | 0(0) | 0(0) | 0(0) | 0(0) | 1(1) | 54 | 32 | 80 | 1.05 |
| 1JW9 | 217 |  | 14 | 1 | 1(1) | 0(0) | 0(0) | 0(0) | 0(0) | 0(0) | 0(0) | 0(0) | 0(0) | 0(0) | 5 | 4 | 72 | 1.02 |
| 1JYO | 531 |  | 249 | 8 | 2(0) | 3(3) | 1(0) | 0(0) | 2(2) | 0(0) | 0(0) | 0(0) | 0(0) | 0(0) | 71 | 41 | 174 | 1.09 |
| 1K9O | 192 |  | 21 | 1 | 0(0) | 1(1) | 0(0) | 0(0) | 0(0) | 0(0) | 0(0) | 0(0) | 0(0) | 0(0) | 9 | 6 | 69 | 1.03 |
| 1KB5 | 206 |  | 750 | 3 | 2(1) | 0(0) | 0(0) | 0(0) | 0(0) | 0(0) | 0(0) | 0(0) | 0(0) | 1(1) | 87 | 38 | 34 | 0.91 |
| 1KSH | 178 |  | 61 | 2 | 1(1) | 0(0) | 0(0) | 1(0) | 0(0) | 0(0) | 0(0) | 0(0) | 0(0) | 0(0) | 15 | 10 | 59 | 1.15 |
| 1KTZ | 109 |  | 15 | 1 | 1(0) | 0(0) | 0(0) | 0(0) | 0(0) | 0(0) | 0(0) | 0(0) | 0(0) | 0(0) | 6 | 4 | 32 | 1.05 |
| 1KXV | 205 |  | 324 | 3 | 1(1) | 0(0) | 0(0) | 0(0) | 0(0) | 0(0) | 0(0) | 1(1) | 0(0) | 1(1) | 51 | 24 | 44 | 1 |
| 1KZ7 | 319 |  | 321 | 4 | 1(1) | 1(0) | 1(1) | 0(0) | 0(0) | 0(0) | 0(0) | 0(0) | 0(0) | 1(1) | 56 | 24 | 75 | 1.05 |
| 1L2I | 88 |  | 22 | 1 | 0(0) | 1(0) | 0(0) | 0(0) | 0(0) | 0(0) | 0(0) | 0(0) | 0(0) | 0(0) | 10 | 4 | 30 | 1.09 |
| 1L4D | 148 |  | 0 | 0 | 0(0) | 0(0) | 0(0) | 0(0) | 0(0) | 0(0) | 0(0) | 0(0) | 0(0) | 0(0) | 0 | 0 | 39 | 1 |
| 1L6X | 153 |  | 0 | 0 | 0(0) | 0(0) | 0(0) | 0(0) | 0(0) | 0(0) | 0(0) | 0(0) | 0(0) | 0(0) | 0 | 0 | 58 | 0.99 |
| 1LFD | 157 |  | 233 | 6 | 4(0) | 1(0) | 0(0) | 0(0) | 0(0) | 0(0) | 0(0) | 0(0) | 0(0) | 1(1) | 39 | 22 | 45 | 1.03 |
| 1LPB | 165 |  | 94 | 2 | 1(1) | 0(0) | 0(0) | 0(0) | 0(0) | 0(0) | 0(0) | 1(1) | 0(0) | 0(0) | 18 | 12 | 44 | 1.04 |
| 1LQV | 76 |  | 32 | 1 | 0(0) | 0(0) | 1(1) | 0(0) | 0(0) | 0(0) | 0(0) | 0(0) | 0(0) | 0(0) | 8 | 6 | 16 | 0.9 |
| 1M9E | 119 |  | 0 | 0 | 0(0) | 0(0) | 0(0) | 0(0) | 0(0) | 0(0) | 0(0) | 0(0) | 0(0) | 0(0) | 0 | 0 | 37 | 1.04 |
| 1MBX | 178 |  | 33 | 2 | 2(1) | 0(0) | 0(0) | 0(0) | 0(0) | 0(0) | 0(0) | 0(0) | 0(0) | 0(0) | 9 | 8 | 46 | 0.97 |
| 1MCT | 111 |  | 119 | 4 | 1(1) | 1(1) | 1(1) | 1(1) | 0(0) | 0(0) | 0(0) | 0(0) | 0(0) | 0(0) | 30 | 19 | 36 | 1.02 |
| 1MCV | 183 |  | 76 | 2 | 1(1) | 0(0) | 0(0) | 0(0) | 0(0) | 1(1) | 0(0) | 0(0) | 0(0) | 0(0) | 18 | 10 | 80 | 1.01 |
| 1MZW | 102 |  | 0 | 0 | 0(0) | 0(0) | 0(0) | 0(0) | 0(0) | 0(0) | 0(0) | 0(0) | 0(0) | 0(0) | 0 | 0 | 44 | 1.05 |
| 1NF3 | 241 |  | 0 | 0 | 0(0) | 0(0) | 0(0) | 0(0) | 0(0) | 0(0) | 0(0) | 0(0) | 0(0) | 0(0) | 0 | 0 | 62 | 0.98 |
| 1NM1 | 208 |  | 36 | 2 | 1(0) | 1(0) | 0(0) | 0(0) | 0(0) | 0(0) | 0(0) | 0(0) | 0(0) | 0(0) | 12 | 10 | 90 | 1.02 |
| 1O6S | 307 |  | 124 | 4 | 1(1) | 1(1) | 1(1) | 1(1) | 0(0) | 0(0) | 0(0) | 0(0) | 0(0) | 0(0) | 33 | 19 | 80 | 1 |
| 1OC0 | 132 |  | 0 | 0 | 0(0) | 0(0) | 0(0) | 0(0) | 0(0) | 0(0) | 0(0) | 0(0) | 0(0) | 0(0) | 0 | 0 | 47 | 1.04 |
| 1OEB | 118 |  | 0 | 0 | 0(0) | 0(0) | 0(0) | 0(0) | 0(0) | 0(0) | 0(0) | 0(0) | 0(0) | 0(0) | 0 | 0 | 36 | 1.03 |
| 1OEY | 146 |  | 62 | 1 | 0(0) | 0(0) | 0(0) | 0(0) | 0(0) | 1(1) | 0(0) | 0(0) | 0(0) | 0(0) | 13 | 7 | 48 | 1 |
| 1OFU | 162 |  | 165 | 4 | 3(2) | 0(0) | 0(0) | 0(0) | 0(0) | 0(0) | 0(0) | 0(0) | 0(0) | 1(0) | 26 | 16 | 61 | 1.04 |
| 1ONQ | 297 |  | 102 | 4 | 2(0) | 1(0) | 0(0) | 1(0) | 0(0) | 0(0) | 0(0) | 0(0) | 0(0) | 0(0) | 29 | 23 | 90 | 1 |
| 1OO0 | 258 |  | 86 | 2 | 0(0) | 1(1) | 0(0) | 0(0) | 0(0) | 1(1) | 0(0) | 0(0) | 0(0) | 0(0) | 19 | 13 | 101 | 1.03 |
| 1OPH | 148 |  | 35 | 2 | 1(1) | 1(1) | 0(0) | 0(0) | 0(0) | 0(0) | 0(0) | 0(0) | 0(0) | 0(0) | 11 | 9 | 56 | 1 |
| 1ORY | 356 |  | 20 | 1 | 1(0) | 0(0) | 0(0) | 0(0) | 0(0) | 0(0) | 0(0) | 0(0) | 0(0) | 0(0) | 6 | 5 | 172 | 1.05 |
| 1OSP | 111 |  | 0 | 0 | 0(0) | 0(0) | 0(0) | 0(0) | 0(0) | 0(0) | 0(0) | 0(0) | 0(0) | 0(0) | 0 | 0 | 21 | 0.92 |
| 1OY3 | 356 |  | 290 | 8 | 5(2) | 1(1) | 0(0) | 0(0) | 0(0) | 0(0) | 0(0) | 1(1) | 0(0) | 1(1) | 67 | 39 | 106 | 1.01 |
| 1P5V | 382 |  | 159 | 7 | 6(2) | 0(0) | 0(0) | 0(0) | 0(0) | 1(0) | 0(0) | 0(0) | 0(0) | 0(0) | 32 | 23 | 128 | 1.05 |
| 1PPF | 84 |  | 0 | 0 | 0(0) | 0(0) | 0(0) | 0(0) | 0(0) | 0(0) | 0(0) | 0(0) | 0(0) | 0(0) | 0 | 0 | 25 | 0.89 |
| 1PXV | 252 |  | 118 | 5 | 3(2) | 1(0) | 0(0) | 1(1) | 0(0) | 0(0) | 0(0) | 0(0) | 0(0) | 0(0) | 31 | 16 | 99 | 0.99 |
| 1Q1S | 327 |  | 68 | 1 | 0(0) | 0(0) | 0(0) | 0(0) | 0(0) | 1(1) | 0(0) | 0(0) | 0(0) | 0(0) | 16 | 8 | 113 | 1 |
| 1Q40 | 368 |  | 364 | 8 | 6(2) | 1(1) | 0(0) | 0(0) | 0(0) | 0(0) | 0(0) | 0(0) | 0(0) | 1(1) | 66 | 32 | 154 | 0.98 |
| 1QAV | 174 |  | 0 | 0 | 0(0) | 0(0) | 0(0) | 0(0) | 0(0) | 0(0) | 0(0) | 0(0) | 0(0) | 0(0) | 0 | 0 | 58 | 0.97 |
| 1QTX | 292 |  | 48 | 3 | 2(0) | 1(0) | 0(0) | 0(0) | 0(0) | 0(0) | 0(0) | 0(0) | 0(0) | 0(0) | 14 | 10 | 130 | 1.03 |
| 1R0R | 154 |  | 20 | 1 | 0(0) | 1(1) | 0(0) | 0(0) | 0(0) | 0(0) | 0(0) | 0(0) | 0(0) | 0(0) | 5 | 5 | 67 | 0.97 |
| 1R17 | 222 |  | 109 | 4 | 2(1) | 0(0) | 1(0) | 1(1) | 0(0) | 0(0) | 0(0) | 0(0) | 0(0) | 0(0) | 27 | 20 | 39 | 1.09 |
| 1REW | 220 |  | 284 | 7 | 2(0) | 3(2) | 0(0) | 0(0) | 0(0) | 0(0) | 0(0) | 0(0) | 2(2) | 0(0) | 69 | 33 | 65 | 1.02 |
| 1RJ9 | 312 |  | 588 | 5 | 2(2) | 0(0) | 1(1) | 0(0) | 0(0) | 0(0) | 0(0) | 0(0) | 0(0) | 2(2) | 91 | 44 | 52 | 1.01 |
| 1RJC | 174 |  | 188 | 5 | 2(1) | 1(1) | 0(0) | 0(0) | 1(1) | 0(0) | 0(0) | 1(1) | 0(0) | 0(0) | 31 | 17 | 48 | 0.97 |
| 1RKE | 264 |  | 100 | 1 | 0(0) | 0(0) | 0(0) | 0(0) | 0(0) | 0(0) | 0(0) | 0(0) | 0(0) | 1(1) | 18 | 9 | 96 | 1 |
| 1S1Q | 129 |  | 0 | 0 | 0(0) | 0(0) | 0(0) | 0(0) | 0(0) | 0(0) | 0(0) | 0(0) | 0(0) | 0(0) | 0 | 0 | 31 | 1 |
| 1S6C | 173 |  | 29 | 2 | 2(0) | 0(0) | 0(0) | 0(0) | 0(0) | 0(0) | 0(0) | 0(0) | 0(0) | 0(0) | 11 | 9 | 43 | 1.16 |
| 1SBW | 164 |  | 68 | 3 | 1(0) | 2(2) | 0(0) | 0(0) | 0(0) | 0(0) | 0(0) | 0(0) | 0(0) | 0(0) | 23 | 15 | 62 | 1 |
| 1SG1 | 223 |  | 248 | 5 | 1(0) | 1(0) | 1(1) | 0(0) | 1(1) | 0(0) | 0(0) | 0(0) | 0(0) | 1(1) | 46 | 24 | 35 | 1.03 |
| 1SGP | 136 |  | 0 | 0 | 0(0) | 0(0) | 0(0) | 0(0) | 0(0) | 0(0) | 0(0) | 0(0) | 0(0) | 0(0) | 0 | 0 | 46 | 1 |
| 1SHW | 133 |  | 0 | 0 | 0(0) | 0(0) | 0(0) | 0(0) | 0(0) | 0(0) | 0(0) | 0(0) | 0(0) | 0(0) | 0 | 0 | 32 | 1.03 |
| 1SKO | 247 |  | 53 | 2 | 1(1) | 0(0) | 0(0) | 1(0) | 0(0) | 0(0) | 0(0) | 0(0) | 0(0) | 0(0) | 14 | 11 | 103 | 1.06 |
| 1SQ2 | 176 |  | 155 | 3 | 0(0) | 1(0) | 0(0) | 1(1) | 0(0) | 0(0) | 0(0) | 1(1) | 0(0) | 0(0) | 38 | 19 | 44 | 0.95 |
| 1STF | 105 |  | 93 | 2 | 0(0) | 0(0) | 0(0) | 2(1) | 0(0) | 0(0) | 0(0) | 0(0) | 0(0) | 0(0) | 20 | 12 | 32 | 1.08 |
| 1SV0 | 105 |  | 17 | 1 | 1(0) | 0(0) | 0(0) | 0(0) | 0(0) | 0(0) | 0(0) | 0(0) | 0(0) | 0(0) | 5 | 4 | 31 | 0.95 |
| 1SVX | 135 |  | 48 | 3 | 3(1) | 0(0) | 0(0) | 0(0) | 0(0) | 0(0) | 0(0) | 0(0) | 0(0) | 0(0) | 15 | 10 | 47 | 1.04 |
| 1T0F | 262 |  | 0 | 0 | 0(0) | 0(0) | 0(0) | 0(0) | 0(0) | 0(0) | 0(0) | 0(0) | 0(0) | 0(0) | 0 | 0 | 136 | 1.01 |
| 1T0P | 126 |  | 23 | 1 | 0(0) | 1(0) | 0(0) | 0(0) | 0(0) | 0(0) | 0(0) | 0(0) | 0(0) | 0(0) | 8 | 6 | 32 | 0.99 |
| 1T8O | 167 |  | 18 | 1 | 1(0) | 0(0) | 0(0) | 0(0) | 0(0) | 0(0) | 0(0) | 0(0) | 0(0) | 0(0) | 8 | 5 | 86 | 1.01 |
| 1TA3 | 232 |  | 143 | 2 | 1(1) | 0(0) | 0(0) | 0(0) | 0(0) | 0(0) | 0(0) | 0(0) | 0(0) | 1(1) | 24 | 15 | 58 | 1 |
| 1TAB | 150 |  | 18 | 1 | 1(1) | 0(0) | 0(0) | 0(0) | 0(0) | 0(0) | 0(0) | 0(0) | 0(0) | 0(0) | 5 | 4 | 57 | 1.02 |
| 1TAW | 144 |  | 36 | 2 | 1(1) | 1(1) | 0(0) | 0(0) | 0(0) | 0(0) | 0(0) | 0(0) | 0(0) | 0(0) | 14 | 10 | 71 | 1.01 |
| 1TGS | 111 |  | 29 | 2 | 2(1) | 0(0) | 0(0) | 0(0) | 0(0) | 0(0) | 0(0) | 0(0) | 0(0) | 0(0) | 7 | 5 | 31 | 0.87 |
| 1TH8 | 172 |  | 24 | 1 | 0(0) | 1(0) | 0(0) | 0(0) | 0(0) | 0(0) | 0(0) | 0(0) | 0(0) | 0(0) | 7 | 5 | 43 | 0.99 |
| 1TO2 | 188 |  | 15 | 1 | 1(0) | 0(0) | 0(0) | 0(0) | 0(0) | 0(0) | 0(0) | 0(0) | 0(0) | 0(0) | 6 | 4 | 72 | 0.96 |
| 1TX4 | 164 |  | 150 | 2 | 0(0) | 0(0) | 0(0) | 0(0) | 0(0) | 0(0) | 2(2) | 0(0) | 0(0) | 0(0) | 30 | 15 | 39 | 0.97 |
| 1U0S | 156 |  | 0 | 0 | 0(0) | 0(0) | 0(0) | 0(0) | 0(0) | 0(0) | 0(0) | 0(0) | 0(0) | 0(0) | 0 | 0 | 45 | 1.02 |
| 1U8T | 96 |  | 0 | 0 | 0(0) | 0(0) | 0(0) | 0(0) | 0(0) | 0(0) | 0(0) | 0(0) | 0(0) | 0(0) | 0 | 0 | 39 | 0.98 |
| 1UAD | 111 |  | 0 | 0 | 0(0) | 0(0) | 0(0) | 0(0) | 0(0) | 0(0) | 0(0) | 0(0) | 0(0) | 0(0) | 0 | 0 | 37 | 1.03 |
| 1UJZ | 165 |  | 65 | 2 | 0(0) | 0(0) | 2(2) | 0(0) | 0(0) | 0(0) | 0(0) | 0(0) | 0(0) | 0(0) | 19 | 10 | 47 | 1 |
| 1UKV | 334 |  | 42 | 3 | 3(2) | 0(0) | 0(0) | 0(0) | 0(0) | 0(0) | 0(0) | 0(0) | 0(0) | 0(0) | 13 | 11 | 73 | 0.96 |
| 1UNL | 286 |  | 194 | 7 | 2(1) | 3(1) | 1(1) | 0(0) | 1(0) | 0(0) | 0(0) | 0(0) | 0(0) | 0(0) | 55 | 28 | 91 | 1.13 |
| 1US7 | 123 |  | 0 | 0 | 0(0) | 0(0) | 0(0) | 0(0) | 0(0) | 0(0) | 0(0) | 0(0) | 0(0) | 0(0) | 0 | 0 | 42 | 1.01 |
| 1USU | 132 |  | 0 | 0 | 0(0) | 0(0) | 0(0) | 0(0) | 0(0) | 0(0) | 0(0) | 0(0) | 0(0) | 0(0) | 0 | 0 | 13 | 0.96 |
| 1UZX | 129 |  | 30 | 2 | 2(1) | 0(0) | 0(0) | 0(0) | 0(0) | 0(0) | 0(0) | 0(0) | 0(0) | 0(0) | 9 | 7 | 27 | 0.98 |
| 1V18 | 385 |  | 0 | 0 | 0(0) | 0(0) | 0(0) | 0(0) | 0(0) | 0(0) | 0(0) | 0(0) | 0(0) | 0(0) | 0 | 0 | 136 | 1 |
| 1V74 | 197 |  | 68 | 2 | 1(1) | 0(0) | 0(0) | 0(0) | 1(1) | 0(0) | 0(0) | 0(0) | 0(0) | 0(0) | 18 | 12 | 43 | 0.94 |
| 1VF6 | 223 |  | 198 | 8 | 2(0) | 4(1) | 1(0) | 1(0) | 0(0) | 0(0) | 0(0) | 0(0) | 0(0) | 0(0) | 48 | 31 | 80 | 1.07 |
| 1VG0 | 300 |  | 13 | 1 | 1(1) | 0(0) | 0(0) | 0(0) | 0(0) | 0(0) | 0(0) | 0(0) | 0(0) | 0(0) | 5 | 4 | 76 | 1 |
| 1VPP | 129 |  | 0 | 0 | 0(0) | 0(0) | 0(0) | 0(0) | 0(0) | 0(0) | 0(0) | 0(0) | 0(0) | 0(0) | 0 | 0 | 47 | 0.95 |
| 1W98 | 376 |  | 45 | 3 | 3(0) | 0(0) | 0(0) | 0(0) | 0(0) | 0(0) | 0(0) | 0(0) | 0(0) | 0(0) | 12 | 9 | 120 | 1.02 |
| 1WEJ | 130 |  | 25 | 2 | 2(2) | 0(0) | 0(0) | 0(0) | 0(0) | 0(0) | 0(0) | 0(0) | 0(0) | 0(0) | 8 | 7 | 41 | 0.96 |
| 1WMH | 119 |  | 57 | 2 | 0(0) | 1(1) | 1(1) | 0(0) | 0(0) | 0(0) | 0(0) | 0(0) | 0(0) | 0(0) | 12 | 9 | 40 | 1.07 |
| 1WQJ | 163 |  | 77 | 2 | 1(1) | 0(0) | 0(0) | 0(0) | 0(0) | 1(0) | 0(0) | 0(0) | 0(0) | 0(0) | 20 | 12 | 28 | 0.97 |
| 1WWW | 186 |  | 52 | 3 | 2(2) | 1(0) | 0(0) | 0(0) | 0(0) | 0(0) | 0(0) | 0(0) | 0(0) | 0(0) | 18 | 11 | 44 | 1.03 |
| 1XB2 | 301 |  | 31 | 2 | 2(2) | 0(0) | 0(0) | 0(0) | 0(0) | 0(0) | 0(0) | 0(0) | 0(0) | 0(0) | 12 | 9 | 69 | 0.99 |
| 1XD3 | 235 |  | 235 | 5 | 3(2) | 1(0) | 0(0) | 0(0) | 0(0) | 0(0) | 0(0) | 0(0) | 0(0) | 1(1) | 45 | 27 | 88 | 1.03 |
| 1XG2 | 228 |  | 67 | 1 | 0(0) | 0(0) | 0(0) | 0(0) | 0(0) | 1(1) | 0(0) | 0(0) | 0(0) | 0(0) | 18 | 9 | 69 | 0.97 |
| 1XL3 | 200 |  | 0 | 0 | 0(0) | 0(0) | 0(0) | 0(0) | 0(0) | 0(0) | 0(0) | 0(0) | 0(0) | 0(0) | 0 | 0 | 84 | 1.08 |
| 1XT9 | 310 |  | 155 | 2 | 0(0) | 0(0) | 0(0) | 0(0) | 0(0) | 0(0) | 2(1) | 0(0) | 0(0) | 0(0) | 31 | 17 | 113 | 0.97 |
| 1XX9 | 233 |  | 0 | 0 | 0(0) | 0(0) | 0(0) | 0(0) | 0(0) | 0(0) | 0(0) | 0(0) | 0(0) | 0(0) | 0 | 0 | 95 | 1.02 |
| 1YCS | 117 |  | 0 | 0 | 0(0) | 0(0) | 0(0) | 0(0) | 0(0) | 0(0) | 0(0) | 0(0) | 0(0) | 0(0) | 0 | 0 | 24 | 0.96 |
| 1YRO | 127 |  | 53 | 3 | 2(2) | 1(0) | 0(0) | 0(0) | 0(0) | 0(0) | 0(0) | 0(0) | 0(0) | 0(0) | 11 | 9 | 26 | 1.04 |
| 2BF8 | 46 |  | 0 | 0 | 0(0) | 0(0) | 0(0) | 0(0) | 0(0) | 0(0) | 0(0) | 0(0) | 0(0) | 0(0) | 0 | 0 | 8 | 1.02 |
| 2BO9 | 248 |  | 143 | 3 | 1(1) | 1(1) | 0(0) | 0(0) | 0(0) | 0(0) | 0(0) | 0(0) | 0(0) | 1(1) | 27 | 17 | 63 | 1.04 |
| 2PRG | 135 |  | 0 | 0 | 0(0) | 0(0) | 0(0) | 0(0) | 0(0) | 0(0) | 0(0) | 0(0) | 0(0) | 0(0) | 0 | 0 | 49 | 1.05 |
| 2PTC | 92 |  | 25 | 1 | 0(0) | 1(1) | 0(0) | 0(0) | 0(0) | 0(0) | 0(0) | 0(0) | 0(0) | 0(0) | 9 | 7 | 29 | 1 |
| 2SIC | 109 |  | 38 | 2 | 1(1) | 1(1) | 0(0) | 0(0) | 0(0) | 0(0) | 0(0) | 0(0) | 0(0) | 0(0) | 8 | 7 | 42 | 0.97 |
| 2TEC | 174 |  | 63 | 4 | 4(3) | 0(0) | 0(0) | 0(0) | 0(0) | 0(0) | 0(0) | 0(0) | 0(0) | 0(0) | 17 | 13 | 74 | 1.05 |
| 2TRC | 299 |  | 157 | 4 | 0(0) | 1(0) | 1(1) | 1(0) | 1(0) | 0(0) | 0(0) | 0(0) | 0(0) | 0(0) | 32 | 21 | 69 | 1.02 |
| 3FAP | 90 |  | 0 | 0 | 0(0) | 0(0) | 0(0) | 0(0) | 0(0) | 0(0) | 0(0) | 0(0) | 0(0) | 0(0) | 0 | 0 | 6 | 0.97 |
| 3TPI | 106 |  | 25 | 1 | 0(0) | 1(1) | 0(0) | 0(0) | 0(0) | 0(0) | 0(0) | 0(0) | 0(0) | 0(0) | 8 | 6 | 40 | 0.96 |
| 4HTC | 220 |  | 37 | 1 | 0(0) | 0(0) | 1(1) | 0(0) | 0(0) | 0(0) | 0(0) | 0(0) | 0(0) | 0(0) | 10 | 6 | 59 | 1.04 |
| 1A2K | 117 |  | 201 | 2 | 0 | 1 | 0 | 0 | 0 | 0 | 0 | 0 | 0 | 1 | 38 | 16 | 29 | 1.08 |
| 1AZZ | 206 |  | 58 | 1 | 0 | 0 | 0 | 0 | 1 | 0 | 0 | 0 | 0 | 0 | 15 | 8 | 83 | 1.03 |
| 1BTH | 145 |  | 128 | 2 | 1 | 0 | 0 | 0 | 0 | 0 | 0 | 0 | 0 | 1 | 24 | 15 | 37 | 0.95 |
| 1CGI | 218 |  | 46 | 2 | 1 | 1 | 0 | 0 | 0 | 0 | 0 | 0 | 0 | 0 | 13 | 10 | 112 | 1.01 |
| 1DFJ | 218 |  | 73 | 5 | 5 | 0 | 0 | 0 | 0 | 0 | 0 | 0 | 0 | 0 | 24 | 19 | 37 | 1.08 |
| 1DKD | 99 |  | 0 | 0 | 0 | 0 | 0 | 0 | 0 | 0 | 0 | 0 | 0 | 0 | 0 | 0 | 54 | 1.02 |
| 1E96 | 123 |  | 0 | 0 | 0 | 0 | 0 | 0 | 0 | 0 | 0 | 0 | 0 | 0 | 0 | 0 | 34 | 0.98 |
| 1EWY | 288 |  | 93 | 6 | 5 | 1 | 0 | 0 | 0 | 0 | 0 | 0 | 0 | 0 | 28 | 23 | 35 | 1.04 |
| 1F34 | 332 |  | 267 | 3 | 1 | 0 | 0 | 0 | 1 | 0 | 0 | 0 | 0 | 1 | 43 | 21 | 88 | 1.06 |
| 1IBR | 429 |  | 411 | 7 | 2 | 0 | 2 | 0 | 0 | 0 | 0 | 0 | 0 | 3 | 92 | 43 | 76 | 1 |
| 1IIL | 317 |  | 169 | 5 | 2 | 1 | 0 | 0 | 2 | 0 | 0 | 0 | 0 | 0 | 37 | 21 | 100 | 1.14 |
| 1IJE | 359 |  | 126 | 3 | 1 | 0 | 1 | 0 | 0 | 0 | 0 | 1 | 0 | 0 | 28 | 20 | 122 | 1.03 |
| 1JTH | 218 |  | 24 | 2 | 0 | 0 | 0 | 0 | 0 | 0 | 0 | 0 | 0 | 0 | 5 | 4 | 88 | 1.02 |
| 1JZD | 226 |  | 79 | 2 | 1 | 0 | 0 | 0 | 0 | 1 | 0 | 0 | 0 | 0 | 19 | 15 | 74 | 1.02 |
| 1KI1 | 256 |  | 92 | 4 | 2 | 1 | 0 | 1 | 0 | 0 | 0 | 0 | 0 | 0 | 27 | 14 | 89 | 1.06 |
| 1M4U | 79 |  | 45 | 2 | 0 | 2 | 0 | 0 | 0 | 0 | 0 | 0 | 0 | 0 | 10 | 7 | 19 | 1.02 |
| 1MEL | 126 |  | 148 | 3 | 2 | 0 | 0 | 0 | 0 | 0 | 0 | 0 | 0 | 1 | 29 | 16 | 39 | 1.01 |
| 1NCA | 160 |  | 56 | 2 | 1 | 0 | 0 | 1 | 0 | 0 | 0 | 0 | 0 | 0 | 17 | 13 | 52 | 0.98 |
| 1NMB | 95 |  | 36 | 2 | 1 | 1 | 0 | 0 | 0 | 0 | 0 | 0 | 0 | 0 | 11 | 10 | 17 | 1.01 |
| 1NW9 | 243 |  | 129 | 3 | 2 | 0 | 0 | 0 | 0 | 0 | 0 | 0 | 0 | 1 | 32 | 17 | 99 | 1.01 |
| 1PDK | 327 |  | 137 | 5 | 2 | 1 | 1 | 1 | 0 | 0 | 0 | 0 | 0 | 0 | 40 | 23 | 113 | 1.06 |
| 1RP3 | 379 |  | 65 | 4 | 3 | 1 | 0 | 0 | 0 | 0 | 0 | 0 | 0 | 0 | 18 | 16 | 149 | 1.03 |
| 1SBB | 129 |  | 27 | 2 | 2 | 0 | 0 | 0 | 0 | 0 | 0 | 0 | 0 | 0 | 6 | 5 | 30 | 1.04 |
| 1SLW | 183 |  | 0 | 0 | 0 | 0 | 0 | 0 | 0 | 0 | 0 | 0 | 0 | 0 | 0 | 0 | 58 | 0.98 |
| 1TTW | 126 |  | 0 | 0 | 0 | 0 | 0 | 0 | 0 | 0 | 0 | 0 | 0 | 0 | 0 | 0 | 20 | 1.09 |
| 1TY4 | 212 |  | 50 | 2 | 0 | 2 | 0 | 0 | 0 | 0 | 0 | 0 | 0 | 0 | 17 | 10 | 63 | 1.07 |
| 1U6H | 223 |  | 90 | 3 | 2 | 0 | 0 | 0 | 0 | 1 | 0 | 0 | 0 | 0 | 22 | 16 | 97 | 1.05 |
| 1WMI | 423 |  | 388 | 6 | 2 | 3 | 0 | 0 | 0 | 0 | 0 | 0 | 0 | 1 | 65 | 30 | 132 | 1.03 |
| 1YDR | 135 |  | 57 | 2 | 0 | 1 | 1 | 0 | 0 | 0 | 0 | 0 | 0 | 0 | 12 | 8 | 38 | 1.02 |
| 2NGR | 209 |  | 221 | 3 | 2 | 0 | 0 | 0 | 0 | 0 | 0 | 0 | 0 | 1 | 39 | 17 | 54 | 1.04 |
