# Supplementary material for: Cavities and Atomic Packing in Protein Structures and Interfaces
Source: PLoS Comput Biol. 2008 Sep 26;4(9):e1000188. doi: 10.1371/journal.pcbi.1000188 (PMC2582456; doi:10.1371/journal.pcbi.1000188)
Supplement: Table S3 — The number of occurrences and the average Voronoi volume (Å3) of the thirteen atom types in (A) the NCNS region and (B) three types of cavities, and (C) considering NCNS and CL atoms together for the tertiary structure. (0.07 MB DOC) [file pcbi.1000188.s009.doc]

Table S3. The number of occurrences and the average Voronoi volume (Å^3^) of the thirteen atom types in (A) the NCNS region and (B) three types of cavities, and (C) considering NCNS and CL atoms together for the tertiary structure.

(A) For NCNS atoms

|  | **Ter_str** |  | **Inter_H** |  | **Inter_C** |  |
| --- | --- | --- | --- | --- | --- | --- |
|  | **Number** | **Volume** | **Number** | **Volume** | **Number** | **Volume** |
| **N** | 35609 | 13.32 | 1577 | 13.86 | 943 | 13.93 |
| **CA** | 26582 | 13.75 | 2337 | 14.36 | 1319 | 14.33 |
| **C** | 36309 | 9.18 | 2065 | 9.49 | 1214 | 9.52 |
| **O** | 24263 | 15.71 | 1632 | 16.55 | 914 | 16.41 |
| **CB** | 16756 | 21.07 | 2057 | 22.65 | 1297 | 22.00 |
| **S** | 893 | 32.48 | 141 | 33.57 | 119 | 30.48 |
| **Oh** | 1582 | 17.98 | 261 | 18.31 | 227 | 17.72 |
| **Oa** | 1688 | 16.59 | 345 | 17.09 | 274 | 17.18 |
| **Na** | 412 | 23.73 | 69 | 24.09 | 71 | 24.48 |
| **Nc** | 797 | 18.82 | 250 | 19.17 | 164 | 19.91 |
| **Nr** | 921 | 15.88 | 143 | 15.72 | 174 | 15.77 |
| **Cc** | 17362 | 16.79 | 2639 | 17.13 | 2116 | 16.75 |
| **Cr** | 21517 | 26.39 | 3285 | 25.50 | 2063 | 23.82 |

(B) For CL atoms

|  | **Ter_str** |  | **Inter_H** |  | **Inter_C** |  |
| --- | --- | --- | --- | --- | --- | --- |
|  | **Number** | **Volume** | **Number** | **Volume** | **Number** | **Volume** |
| **N** | 360 | 15.73 | 61 | 15.27 | 32 | 16.01 |
| **CA** | 468 | 15.61 | 90 | 16.31 | 68 | 16.14 |
| **C** | 104 | 10.28 | 17 | 10.46 | 9 | 10.39 |
| **O** | 476 | 18.48 | 67 | 18.57 | 49 | 19.15 |
| **CB** | 565 | 25.25 | 89 | 24.08 | 39 | 23.56 |
| **S** | 38 | 37.44 | 7 | 33.51 | 4 | 32.98 |
| **Oh** | 71 | 21.04 | 13 | 21.52 | 4 | 23.20 |
| **Oa** | 74 | 20.28 | 17 | 19.27 | 13 | 21.06 |
| **Na** | 14 | 26.75 | 7 | 26.63 | 5 | 26.75 |
| **Nc** | 37 | 24.51 | 14 | 23.60 | 10 | 22.35 |
| **Nr** | 30 | 19.37 | 10 | 18.64 | 5 | 18.99 |
| **Cc** | 374 | 22.60 | 82 | 22.37 | 56 | 22.00 |
| **Cr** | 893 | 33.25 | 124 | 32.07 | 78 | 30.79 |

(C) For NCNS + CL atoms in the tertiary structure

|  | **Ter_str** |  |
| --- | --- | --- |
|  | **Number** | **Volume** |
| **N** | 35969 | 13.34 |
| **CA** | 27050 | 13.78 |
| **C** | 36413 | 9.19 |
| **O** | 24739 | 15.76 |
| **CB** | 17321 | 21.21 |
| **S** | 931 | 32.68 |
| **Oh** | 1653 | 18.12 |
| **Oa** | 1762 | 16.75 |
| **Na** | 426 | 23.83 |
| **Nc** | 834 | 19.07 |
| **Nr** | 951 | 15.99 |
| **Cc** | 17736 | 16.92 |
| **Cr** | 22410 | 26.66 |
