# Supplementary material for: Cavities and Atomic Packing in Protein Structures and Interfaces
Source: PLoS Comput Biol. 2008 Sep 26;4(9):e1000188. doi: 10.1371/journal.pcbi.1000188 (PMC2582456; doi:10.1371/journal.pcbi.1000188)
Supplement: Table S4 — Details of the ligands present in cavities. (0.11 MB DOC) [file pcbi.1000188.s010.doc]

Table S4. Details of the ligands present in cavities

| **PDB Code** | **Ligands in cavities** | | | | **#water molecules in cavity** | **Cavity volume (Å^3^)** |
| --- | --- | --- | --- | --- | --- | --- |
|  | **Name** | **Formula** | **# non-H atoms** | **# atoms in cavity** |  |  |
| **(a) Tertiary structure** | | | | | | |
| 1A7V* | PROTOPORPHYRIN IX CONTAINING FE | C34 H32 Fe N4 O4 | 43 | 3 | 0 | 18.06 |
| 1ATL | CALCIUM ION | CA | 1 | 1 | 1 | 15.23 |
| 1B12* | (5S,6S)-6-[(R)ACETOXYETH-2-YL]-PENEM-3-CARBOXYLATEPROPANE | C13 H17 N1 O5 S1 | 20 | 1 | 1 | 13.21 |
| 1E02 | UNDECANAL | C11 H22 O | 12 | 12 | 0 | 501.02 |
| 1FEH | FE2/S2 (INORGANIC) CLUSTER | Fe2 S2 | 4 | 4 | 0 | 85.33 |
| 1FEH | IRON/SULFUR CLUSTER | Fe4 S4 | 8 | 8 | 0 | 165.17 |
| 1FEH | IRON/SULFUR CLUSTER | Fe4 S4 | 8 | 8 | 0 | 188.79 |
| 1FEH | IRON/SULFUR CLUSTER | Fe4 S4 | 8 | 8 | 0 | 190.94 |
| 1FEH | 2 IRON/2 SULFUR/5 CARBONYL/2 WATER INORGANIC CLUSTER & IRON/SULFUR CLUSTER | C5 H8 Fe2 O7 S2 & Fe4 S4 | 24 | 24 | 1 | 512.01 |
| 1FM1 | CALCIUM ION | CA | 1 | 1 | 0 | 17.21 |
| 1G2A | NICKEL (II) ION | NI1 | 1 | 1 | 3 | 72.19 |
| 1QPA | CALCIUM ION | CA | 1 | 1 | 2 | 17.37 |
| 1THZ | POTASSIUM ION | K1 | 1 | 1 | 0 | 19.97 |
| 2SCP | CALCIUM ION | CA | 1 | 1 | 1 | 17.18 |
| 1chm | CARBAMOYL SARCOSINE | C4 H8 N2 O3 | 9 | 9 | 4 | 289.93 |
| 1mor | ALPHA-D-GLUCOSE-6-PHOSPHATE | C6 H13 O9 P | 16 | 16 | 9 | 565.07 |
| 1a4i* | NADPH DIHYDRO-NICOTINAMIDE-ADENINE-DINUCLEOTIDE PHOSPHATE | C21 H30 N7 O17 P3 | 48 | 1 | 1 | 18.43 |
| 1alo | FE2/S2 (INORGANIC) CLUSTER | Fe2 S2 | 4 | 4 | 0 | 73.11 |
| 1alo | FE2/S2 (INORGANIC) CLUSTER | Fe2 S2 | 4 | 4 | 1 | 144.77 |
| 1alo* | CHLORIDE ION & ISOPROPYL ALCOHOL | Cl & C3 H8 O | 5 | 2 | 4 | 188.3 |
| 1alo | ISOPROPYL ALCOHOL | C3 H8 O | 4 | 4 | 6 | 351.26 |
| 1aq6 | FORMIC ACID | C H2 O2 | 3 | 3 | 3 | 132.25 |
| 1bbh* | PROTOPORPHYRIN IX CONTAINING FE | C34 H32 Fe N4 O4 | 43 | 1 | 0 | 11.94 |
| 1biq | MERCURY (II) ION | HG | 1 | 1 | 0 | 14.15 |
| 1biq | MERCURY (II) ION | HG | 1 | 1 | 0 | 27.25 |
| 1biq | MERCURY (II) ION | HG | 1 | 1 | 0 | 104.98 |
| 1biq | FE (II) ION & HYDROXIDE ION & 3-HYDROXYPHENYLALANINE | FE1 & H1 O1 & C9 H11 N1 O3 | 16 | 16 | 0 | 347.14 |
| 1bjw* | 2-LYSINE(3-HYDROXY-2-METHYL-5-PHOSPHONOOXYMETHYL- PYRIDIN-4-YLMETHANE) | C14 H24 N3 O7 P | 25 | 1 | 0 | 11.79 |
| 1bjw* | 2-LYSINE(3-HYDROXY-2-METHYL-5-PHOSPHONOOXYMETHYL- PYRIDIN-4-YLMETHANE) | C14 H24 N3 O7 P | 25 | 1 | 2 | 62.45 |
| 1bjw* | 2-LYSINE(3-HYDROXY-2-METHYL-5-PHOSPHONOOXYMETHYL- PYRIDIN-4-YLMETHANE) | C14 H24 N3 O7 P | 25 | 1 | 1 | 81.5 |
| 1bmd* | NICOTINAMIDE-ADENINE-DINUCLEOTIDE | C21 H27 N7 O14 P2 | 44 | 3 | 1 | 35.26 |
| 1brw | CALCIUM ION | CA | 1 | 1 | 0 | 21.32 |
| 1bxk* | NICOTINAMIDE-ADENINE-DINUCLEOTIDE | C21 H27 N7 O14 P2 | 44 | 1 | 1 | 20.21 |
| 1cvu* | ARACHIDONIC ACID | C20 H32 O2 | 22 | 1 | 1 | 35.85 |
| 1czj* | PROTOPORPHYRIN IX CONTAINING FE | C34 H32 Fe N4 O4 | 43 | 1 | 0 | 13.4 |
| 1kpf* | ADENOSINE MONOPHOSPHATE | C10 H14 N5 O7 P | 23 | 1 | 4 | 116 |
| 1oac* | 5-(2-CARBOXY-2-AMINOETHYL)-2-HYDROXY-1,4-BENZOQUINONE & CALCIUM ION | C9 H9 N O5 & CA | 16 | 15 | 3 | 460.76 |
| 1r2f | 2 FE (III) ION | Fe | 2 | 2 | 0 | 18.47 |
| 1slt* | OXYGENS BOUND TO CYS SG | O3 | 3 | 1 | 0 | 38.3 |
| 1sox* | DIMERCAPTO- 4-OXO-3,7,8A,9,10,10A-HEXAHYDRO-4H-8-OXA- 1,3,9,10-TETRAAZA-ANTHRACEN-7-YLMETHYL)ESTER | C10 H14 N5 O6 P S2 | 24 | 2 | 1 | 21.2 |
| 1sox* | MOLYBDENUM ATOM & DIMERCAPTO- 4-OXO-3,7,8A,9,10,10A-HEXAHYDRO-4H-8-OXA- 1,3,9,10-TETRAAZA-ANTHRACEN-7-YLMETHYL)ESTER | Mo & C10 H14 N5 O6 P S2 | 25 | 23 | 2 | 464.26 |
| 1uby | MAGNESIUM ION | Mg | 1 | 1 | 0 | 15.98 |
| 8prk* | MANGANESE (II) ION & PHOSPHATE ION | Mn & O4 P | 6 | 2 | 3 | 44.75 |
| **(b) Homodimer interface** | | | | | | |
| 1ctt | 3,4-DIHYDRO-1H-PYRIMIDIN-2-ONE NUCLEOSIDE | C9 H14 N2 O5  & Zn | 17 | 17 | 1 | 346.11 |
| 1ctt | 3,4-DIHYDRO-1H-PYRIMIDIN-2-ONE NUCLEOSIDE | C9 H14 N2 O5  & Zn | 17 | 17 | 1 | 346.1 |
| 1czj* | PROTOPORPHYRIN IX CONTAINING FE | C34 H32 Fe N4 O4 | 43 | 1 | 0 | 12.06 |
| 1czj* | PROTOPORPHYRIN IX CONTAINING FE | C34 H32 Fe N4 O4 | 43 | 1 | 0 | 12.11 |
| 1nse | ACETATE ION | C2 H3 O2 | 4 | 3 | 0 | 142.31 |
| **(c) Heterocomplex interface** | | | | | | |
| 1E44 | 1,2-ETHANEDIOL | C2 H6 O2 | 4 | 4 | 8 | 355.24 |
| 1STF | CARBOXYMETHYL GROUP | C2 O2 | 4 | 4 | 1 | 47.24 |
